# A complete nicotinate degradation pathway in the microbial eukaryote *Aspergillus nidulans*

**DOI:** 10.1038/s42003-022-03684-3

**Published:** 2022-07-21

**Authors:** Eszter Bokor, Judit Ámon, Mónika Varga, András Szekeres, Zsófia Hegedűs, Tamás Jakusch, Zsolt Szakonyi, Michel Flipphi, Csaba Vágvölgyi, Attila Gácser, Claudio Scazzocchio, Zsuzsanna Hamari

**Affiliations:** 1 University of Szeged Faculty of Science and Informatics, Department of Microbiology, Szeged, Hungary; 2 University of Szeged Faculty of Science and Informatics, Department of Inorganic and Analytical Chemistry, Szeged, Hungary; 3 University of Szeged Faculty of Pharmacy, Institute of Pharmaceutical Chemistry, Szeged, Hungary; 4 Institute de Génétique et Microbiologie, Université Paris-Sud, Orsay, France; 5 HCEMM-USZ Fungal Pathogens Research Group, University of Szeged Faculty of Science and Informatics, Department of Microbiology, Szeged, Hungary; 6 MTA-SZTE “Lendület” Mycobiome Research Group, University of Szeged, Szeged, Hungary; 7 Section of Microbiology, Department of Infectious Diseases, Imperial College, London, United Kingdom; 8 Université Paris-Saclay, CEA, CNRS, Institute for Integrative Biology of the Cell (I2BC), 91198 Gif-sur-Yvette, France; 9 Present Address: Department of Biochemical Engineering, Faculty of Science and Technology, University of Debrecen, Debrecen, Hungary

**Keywords:** Microbiology, Metabolic pathways

## Abstract

Several strikingly different aerobic and anaerobic pathways of nicotinate breakdown are extant in bacteria. Here, through reverse genetics and analytical techniques we elucidated in *Aspergillus nidulans*, a complete eukaryotic nicotinate utilization pathway. The pathway extant in this fungus and other ascomycetes, is quite different from bacterial ones. All intermediate metabolites were identified. The cognate proteins, encoded by eleven genes (*hxn*) mapping in three clusters are co-regulated by a specific transcription factor. Several enzymatic steps have no prokaryotic equivalent and two metabolites, 3-hydroxypiperidine-2,6-dione and 5,6-dihydroxypiperidine-2-one, have not been identified previously in any organism, the latter being a novel chemical compound. Hydrolytic ring opening results in α-hydroxyglutaramate, a compound not detected in analogous prokaryotic pathways. Our earlier phylogenetic analysis of Hxn proteins together with this complete biochemical pathway illustrates convergent evolution of catabolic pathways between fungi and bacteria.

## Abstract (web summary)

A novel nicotinate degradation pathway is described in *Aspergillus nidulans*, with metabolic products identified that were not previously found in prokaryotic species. This is the first such pathway to be described in a eukaryote.

## Introduction

Nicotinic acid (niacin, vitamin B3), a precursor of NAD, can serve as a nitrogen and carbon source in bacteria. In prokaryotes nicotinic acid (NA) is first converted to 6-hydroxynicotinic acid (6-NA), a reaction catalyzed by MOCO (molybdenum cofactor)-containing nicotinate hydroxylase enzymes (reviewed in ref. ^1^), which evolved several times independently^2–4^. Four quite different pathways metabolizing 6-NA have been described in detail in bacteria^5^.

**Fig. 1:**
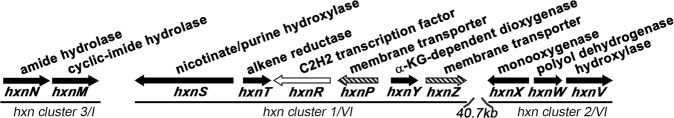
Summary of organization and function of HxnR-regulon composed of three gene clusters in *A. nidulans*^11^. Arrows indicate specific *hxn* genes and relative gene orientation. Lines below the genes indicate gene clusters. Names of clusters are indicated below the lines. Above the arrows, reported roles of genes (*hxnS* and *hxnR*^10^) or roles deduced from domain functions (*hxnN,M,T,P,Y,Z,X,W,V*^11^) are indicated. Black arrows indicate enzyme gene products, striped arrows indicate transporter gene products, and the white arrow denotes the pathway-specific transcription factor.

The only detailed study of nicotinate utilization in a eukaryotic microorganism was carried out by us in the ascomycete *Aspergillus nidulans*. A nicotinate hydroxylase was characterized, and mutants in a gene encoding this enzyme and a putative transcription factor necessary for its induction were described^6–10^. The genes encoding nicotinate hydroxylase (HxnS) and the HxnR transcription factor map in a six-gene co-regulated cluster (including also *hxnZ,Y,P* and *T*, cluster hxn1/VI)^10^. Recently, five additional *hxn* genes (*hxnX, W, V, N*, and *M*) were identified as members of the HxnR-regulon. In *A. nidulans*, these map in two additional gene clusters (hxn2/VI and hxn3/I clusters)^11^ (Fig. 1). All the *hxn* genes are induced by a hitherto non-determined derivative of nicotinic acid (further referred to as the physiological inducer)^10^. Induced levels of expression necessitate both the pathway-specific transcription factor HxnR and the GATA factor AreA, mediating nitrogen metabolite de-repression^10,11^. The *hxnR* gene is characterized by both loss-of-function (including deletions) and constitutive mutants^10^.

In *Aspergillus terreus*, an RNASeq study determined that growth in the presence of salicylate results in induction of *hxnS* and *hxnX* orthologues through 3-hydroxyanthranilate-coupled quinolinate degradation^12^. This suggests that in this organism, either a common inducer metabolite occurs in the nicotinate and salicylate degradation pathways, or that in the latter pathway an additional metabolite can act as a positive effector of HxnR.

In this work, we establish the complete nicotinate degradation pathway in the ascomycete filamentous fungus *A. nidulans* by using reverse genetics and by ultra-high-performance liquid chromatography—high-resolution mass spectrometry (UHPLC-HRMS) based analysis of pathway metabolites, followed by purification and NMR analysis of two novel compounds. This work illustrates the convergent evolution of metabolic pathways in phylogenetically very distant microorganisms.

## Results and discussion

**Fig. 2:**
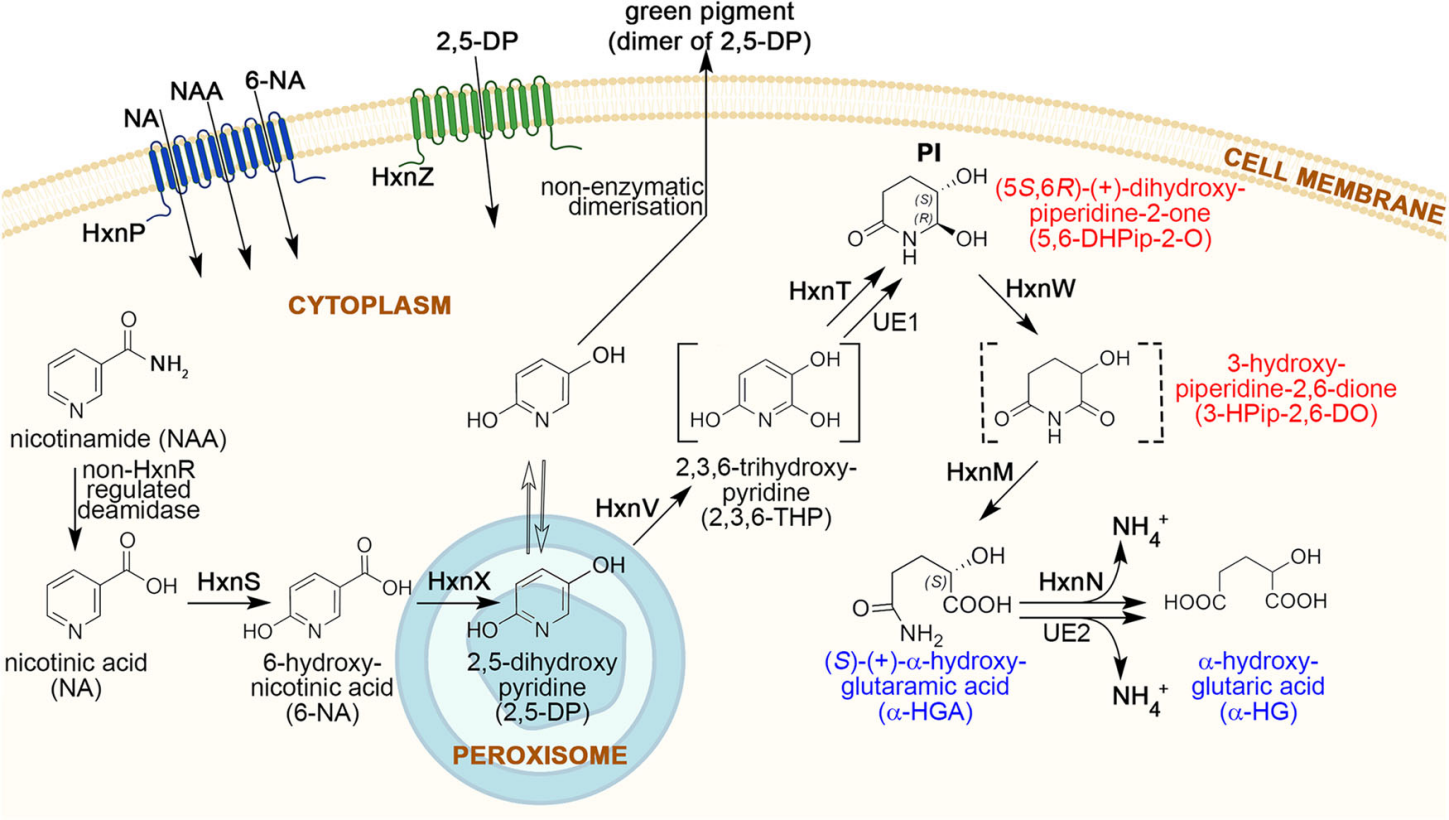
Nicotinate catabolic route in *A. nidulans*. HxnP and HxnZ are transporters (represented by blue and green transmembrane domains, respectively) that transport the indicated compounds. HxnS hydroxylates nicotinic acid (NA) to 6-hydroxynicotinic acid (6-NA). HxnX operates in peroxisomes and converts 6-NA to 2,5-dihydroxypyridine (2,5-DP), which is subsequently hydroxylated by HxnV to 2,3,6-trihydroxypyridine (2,3,6-THP). HxnT and a yet-unknown alkene reductase (UE1) partially saturate the pyridine ring of 2,3,6-THP to (5*S*,6R)-( + )-dihydroxypiperidine-2-one (5,6-DHPip-2-O), which is then converted to 3-hydroxypiperidine-2,6-dione (3-HPip-2,6-DO) by HxnW, a NAD-dependent polyol dehydrogenase type enzyme. The ring of 3-HPip-2,6-DO is opened by the cyclic imidase HxnM between N-C2 resulting in (*S*)-( + )-α-hydroxyglutaramate (α-HGA) formation. The nitrogen is salvaged by HxnN amide hydrolase and results in α-hydroxyglutarate (α-HG) formation. This reaction can also be catalyzed by other amide hydrolases (UE2). NA can be formed endogenously by the hydrolytic cleavage of amide group of nicotinamide (NAA) by a non-HxnR regulated deamidase. Cellular components such as cell membrane, cytoplasm, and peroxisome are shown and indicated by pictograms. Reaction in the peroxisome pictogram indicates the spatial separation of the referred catabolic step in the peroxisomes. The compound in square brackets denotes a predicted intermediate that was not detected by the UHPLC-HRMS method but deduced from the structure of the identified upstream and downstream metabolites. The structure of the compound in the dashed square brackets was deduced by the exact *m*/*z* value and MS/MS fragmentation pattern of the compound obtained by UHPLC-HRMS (Supplementary Table 1), the UHPLC-HRMS and NMR confirmed structures of the upstream and downstream metabolites (Supplementary Tables 1, 2) in line with the proposed ketoreductase activity of the HxnW. UE: unidentified enzyme. PI: physiological metabolite inducer of the pathway-related *hxn* genes; Compound names in red lettering denote metabolites, which have never been detected before neither in eukaryotic nor in prokaryotic organisms. Compound names in blue lettering denote metabolites not detected in prokaryotic NA catabolic pathways. (Created with BioRender.com).

Figure 2 shows the pathway of NA utilization in *Aspergillus nidulans*. The rationale for this pathway is detailed below.

**Fig. 3:**
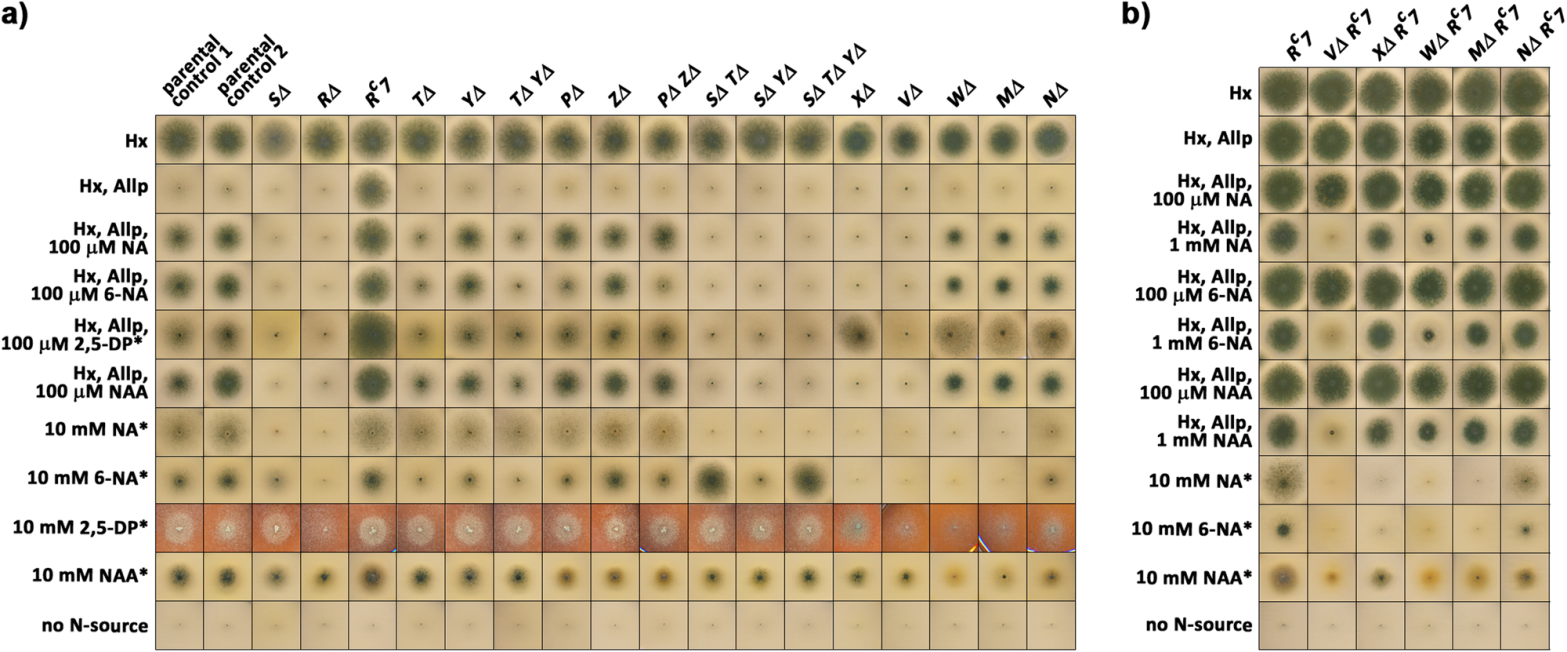
Utilization, inducer, and inhibition tests of *hxn* mutants. **a** Utilization of different nitrogen sources by mutants described in this article in an *hxnR*^*+*^ wild-type background (except for *hxnRΔ* and *hxnR*^*c*^*7* controls). **b** Utilization of different nitrogen sources by some *hxn* gene deletion mutants in an *hxnR*^*c*^*7* (constitutive) background. Above each column, we indicate the relevant mutation carried by each tested strain. Hx indicates 1 mM hypoxanthine as the sole nitrogen source. Hx, Allp, as above including 5.5 µM allopurinol, which fully inhibits HxA but not HxnS (therefore Hx utilization depends on the activation of HxnR-regulon-belonging HxnS (for details see^10^). NA and 6-NA indicate, respectively, nicotinic acid and 6-OH nicotinic acid added as the sodium salts (see Methods section). 2,5-DP and NAA indicate, 2,5-dihydroxypyridine and nicotinamide, respectively. Other relevant concentrations are indicated in the figure. Plates were incubated for 3 days at 37 °C except those marked by an asterisk (*), which were incubated for 4 days. The relevant *hxn* genes are symbolized by only the capital letter indicating the locus name. Strains used: parental control 1 (HZS.120, parent of *SΔ, TΔ, YΔ*), parental control 2 (TN02 A21, parent of *RΔ*, *PΔ, ZΔ, XΔ*, *VΔ, NΔ*) are wildtype for all *hxn* genes. Mutant strains: *SΔ* (HZS.599), *RΔ* (HZS.614), *R*^*c*^*7* (FGSC A872), *TΔ* (HZS.222), *YΔ* (HZS.223), *TΔ YΔ* (HZS.502), *PΔ* (HZS.221), *ZΔ* (HZS.226), *PΔ ZΔ* (HZS.480), *SΔ TΔ* (HZS.892), *SΔ YΔ* (HZS.558), *SΔTΔ YΔ* (HZS.569), *VΔ* (HZS.294), *XΔ* (HZS.726), *WΔ* (HZS.393), *MΔ* (HZS.293), *NΔ* (HZS.288), *VΔ R*^*c*^*7* (HZS.309), *XΔ R*^*c*^*7* (HZS.310), *WΔ R*^*c*^*7* (HZS.517), *MΔ R*^*c*^*7* (HZS.308), and *NΔ R*^*c*^*7* (HZS.306). The complete genotypes are given in Supplementary Table 3.

**Fig. 4:**
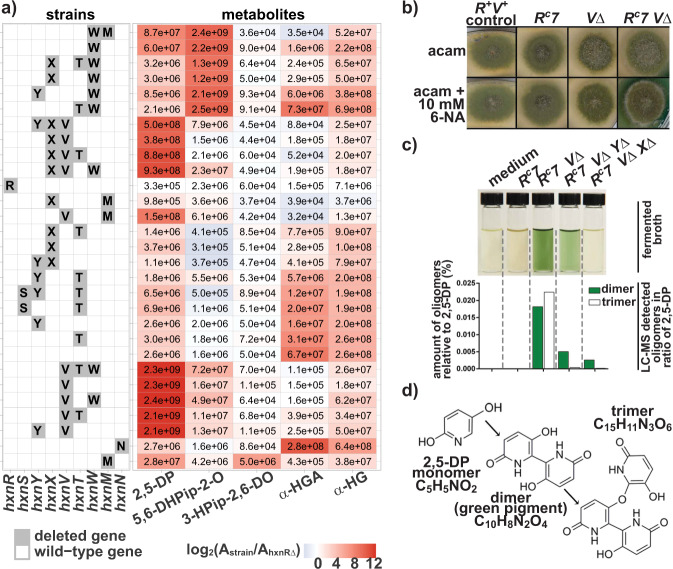
Accumulation of metabolites in various single and multiple *hxn* gene deletion mutants in a constitutive *hxnR*^*c*^*7* background. **a** Heat map of selected metabolites in control strains and NA-catabolism impaired single and multi-deletion strains. The table to the left of the heat map indicates the genotype of the used strains by a single letter code for each of the deleted *hxn* genes. The row, where no *hxn* gene deletion is indicated refers to the *hxnR*^*c*^*7* control strain. The heat map shows UHPLC-HRMS measured metabolites for each strain. Numbers within the cells correspond to raw peak area values (they are averages of three biological replicates; coefficient of variation was less than 10% for each cases), whereas the heat map colors correspond to log_2_ fold change of peak area values relative to that of the transcription factor-deleted strain (*hxnRΔ*) (Supplementary Table 1). The data shown was obtained from mycelial extracts, except that the 3-HPip-2,6-DO compound was detected and measured exclusively in the culture broth. Abbreviated compounds 2,5-DP: 2,5-dihydroxypyridine, 5,6-DHPip-2-O: (5*S*,6*R*)-( + )-dihydroxypiperidine-2-one, 3-HPip-2,6-DO: 3-hydroxypiperidine-2,6-dione, α-HGA: (*S*)-( + )-α-hydroxyglutaramate, α-HG: α-hydroxyglutarate. Strains used (the relevant *hxn* genes are symbolized by only the capital letter indicating the locus name): *WΔ MΔ* (HZS.588), *WΔ R*^*c*^*7* (HZS.517), *XΔ TΔ WΔ R*^*c*^*7* (HZS.904), *XΔ WΔ R*^*c*^*7* (HZS.751), *YΔ WΔ R*^*c*^*7* (HZS.898), *TΔ WΔ R*^*c*^*7* (HZS.894), *YΔ XΔ VΔ R*^*c*^*7* (HZS.901), *XΔ VΔ R*^*c*^*7* (HZS.783), *XΔ VΔ TΔ R*^*c*^*7* (HZS.899), *XΔ VΔ WΔ R*^*c*^*7* (HZS.750), *RΔ* (HZS.614), *XΔ MΔ* (HZS.582), *VΔ MΔ* (HZS.584), *XΔ TΔ R*^*c*^*7* (HZS.798), *XΔ R*^*c*^*7* (HZS.812), *YΔ XΔ R*^*c*^*7* (HZS.810), *YΔ TΔ R*^*c*^*7* (HZS.903), *STΔ YΔ R*^*c*^*7* (HZS.912), *STΔ R*^*c*^*7* (HZS.911), *YΔ R*^*c*^*7* (HZS.429), *TΔ R*^*c*^*7* (HZS.427), *R*^*c*^*7* (FGSC A872), *VΔ TΔ WΔ R*^*c*^*7* (HZS.902), *VΔ R*^*c*^*7* (HZS.309), *VΔ WΔ R*^*c*^*7* (HZS.749), *VΔ TΔ R*^*c*^*7* (HZS.748), *YΔ VΔ R*^*c*^*7* (HZS.747), *NΔ R*^*c*^*7* (HZS.306), and *MΔ R*^*c*^*7* (HZS.308). The complete genotypes are given in Supplementary Table 3. **b** Green pigment formation from 2,5-DP on solid medium in an *hxnR*^*c*^*7 hxnVΔ* strain. Strains were grown on MM with 10 mM acetamide (acam) as the sole N-source without or with the addition of 10 mM 6-NA (as the sodium salt). Strains used in this experiment: *R*^*+*^ *V*^*+*^: *hxnR*^*+*^ *hxnV*^*+*^ control (HZS.120); *R*^*c*^*7*: *hxnR*^*c*^*7* (FGSC A872); *VΔ*: *hxnVΔ* (HZS.294); *R*^*c*^*7 VΔ*: *hxnR*^*c*^*7 hxnVΔ* (HZS.309). The complete genotypes are given in Supplementary Table 3. **c** Green pigment formation in the culture broth of each tested strain, compared with sterile medium. Below, the UHPLC-HRMS measured amounts of the dimer (green pigment) and trimer forms of 2,5-DP in the corresponding broths relative to the amount of 2,5-DP (%) are shown. Strains were grown in MM with 10 mM acetamide as the sole N-source for 14 h and the mycelia were then transferred to MM without acetamide but supplemented with 10 mM 6-NA (as the sodium salt) substrate and further incubated for 24 h. The color of filtered ferment broths were photographed and subsequently analyzed by UHPLC-HRMS (see Methods section). Strains used in this experiment: *R*^*c*^*7* and *R*^*c*^*7 VΔ* are the same as in **b**; *R*^*c*^*7 VΔ YΔ*: *hxnR*^*c*^*7 hxnVΔ hxnYΔ* (HZS.747) and *R*^*c*^*7 VΔ XΔ*: *hxnR*^*c*^*7 hxnVΔ hxnXΔ* (HZS.783). **d** Structures of dimer and trimer forms of 2,5-DP. Deletion of *hxnV* results in the accumulation of 2,5-DP, which is non-enzymatically transformed into dimer and trimer forms. UHPLC-HRMS results for 2,5-DP are detailed in panel a. Retention times of dimer and trimer forms were 1.58 and 5.63 min, and the accurate masses of precursor ions [M + H]^+^ were 221.0556 and 330.0738, respectively.

We systematically deleted all *hxn* genes (*hxnS* and *hxnR* deletions were published previously^10^) in both *hxnR*^*+*^ (wildtype) and *hxnR*^*c*^*7* (where the HxnR transcription factor is constitutively active) backgrounds. The resulting strains were tested for the utilization of the commercially available NA derivatives as N-sources or as inducer precursors (Fig. 3a). Catabolism of 6-NA in these strains was tracked by UHPLC-HRMS followed by the identification of the chemical structure of two purified metabolites by NMR (Fig. 4a and Supplementary Tables 1, 2).

The growth tests indicate whether the tested metabolites are a nitrogen source for each strain, but also, whether in a given deletion strain the hitherto unidentified physiological inducer metabolite is synthesized or not (Fig. 3a). To this end we monitor the induction of *hxnS*. HxnS can catalyze the hydroxylation of hypoxanthine (Hx) to xanthine, which is further converted to uric acid by the XanA enzyme^7,13–15^, and differently from the canonical xanthine dehydrogenase (HxA), HxnS is resistant to allopurinol (Allp) inhibition^10,13^. Thus, if the physiological inducer metabolite is produced, a given strain would utilize Hx as a nitrogen source in the presence of Allp (Fig. 3a). This growth on Hx may be diminished or abolished if the accumulated pathway metabolite is toxic (Fig. 3a, b).

### Transporters

Two genes, *hxnP* and *hxnZ* map in cluster 1/VI and encode putative transporters of the Major Facilitator Superfamily with 12-transmembrane domains (Supplementary Fig. 1a, c)^11^. The nearest characterized homolog of HxnP is the high-affinity nicotinate transporter TNA1 of *S. cerevisiae* (27% identity), while there is no close characterized homolog of HxnZ. The most likely orthologue of TNA1 in *A. nidulans* (encoded by AN5650 and sharing 31% amino acid (AA) identity with TNA1) and also its apparent paralogue in the genome (AN11116) show higher similarity with TNA1 than HxnP. While expression of AN5650 is completely independent from HxnR and NA or 6-NA induction (Supplementary Fig. 1b), *hxnP* shows a pattern of regulation identical to that of *hxnS* and the other enzyme-encoding genes of the clusters^10^. This may signify a divergence in substrate specificity and/or redundancy of nicotinate transporters.

Deletion of *hxnZ* impairs, but not abolishes the growth on 2,5-dihydroxypyridine (2,5-DP) and nicotinamide (NAA) as a nitrogen source and did not result in any visible impairment of growth on either NA or 6-NA (Fig. 3a). Deletion of *hxnP* affects very slightly the utilization of NA as nitrogen source, but clearly that of 6-NA and NAA (Fig. 3a). The inducer test on Hx as N-source supplemented with Allp and an inducer precursor showed that a deletion of *hxnZ* affects growth slightly, while deletion of *hxnP* clearly affects the uptake of 6-NA compared to their parental control (control 2 on Fig. 3a). The phenotype of *hxnPΔ hxnZΔ* double mutants is identical to that of the *hxnP* single mutant (Fig. 3a). Deletion of either *hxnP* or *hxnZ* does not affect the nicotinate supplementation of *nicB8* auxotrophy, which can be achieved at much lower concentrations of NA (as low as 1 µM) than that those necessary for its utilization as a sole nitrogen source (10 mM) or as inducer precursor of *hxn* genes (100 µM). This is consistent with redundancy of NA transporters in the genome and with HxnP encoding a low-affinity transporter for 6-NA, NAA, and NA (Fig. 2).

### Nicotinamide utilization

One equivalent of N can be obtained by deamination of one molecule NAA through the action of a NAA deaminase, similar to Pnc1p in *S. cerevisiae*^16^, independently from further catabolism of NA (see the growth of *hxnRΔ* and *hxnSΔ* in Fig. 3a). The putative NAA deaminase of *A. nidulans* encoded on Chromosome II (AN3809) is well expressed under conditions where the genes of the HxnR-regulon are not expressed at all (RNAseq experiments by^17^), thus the expression of this gene must be independent of NA induction and HxnR function. The impaired utilization of NAA by *hxnWΔ*, *hxnMΔ*, and *hxnNΔ* strains compared to *hxnRΔ*, where no *hxn* gene is expressed, is a diagnostic test of the toxicity of accumulated metabolic intermediates (Fig. 3a, b).

### Conversion of 6-NA to 2,5-DP occurs in the peroxisome by the 6-NA monooxygenase HxnX

Previous work has shown that HxnS catalyzes the hydroxylation of NA to 6-NA (ref. ^10^ and references therein). Deletion of *hxnX* prevents the utilization of 6-NA but not 2,5-DP as a nitrogen source (Fig. 3a). Strains deleted for this gene are also defective in the induction of *hxnS* by 6-NA but not by 2,5-DP and an *hxnX* deletion blocks the 2,5-DP accumulation in *hxnR*^*c*^*7 hxnVΔ* mutant (Figs. 3a,4a, b).

**Fig. 5:**
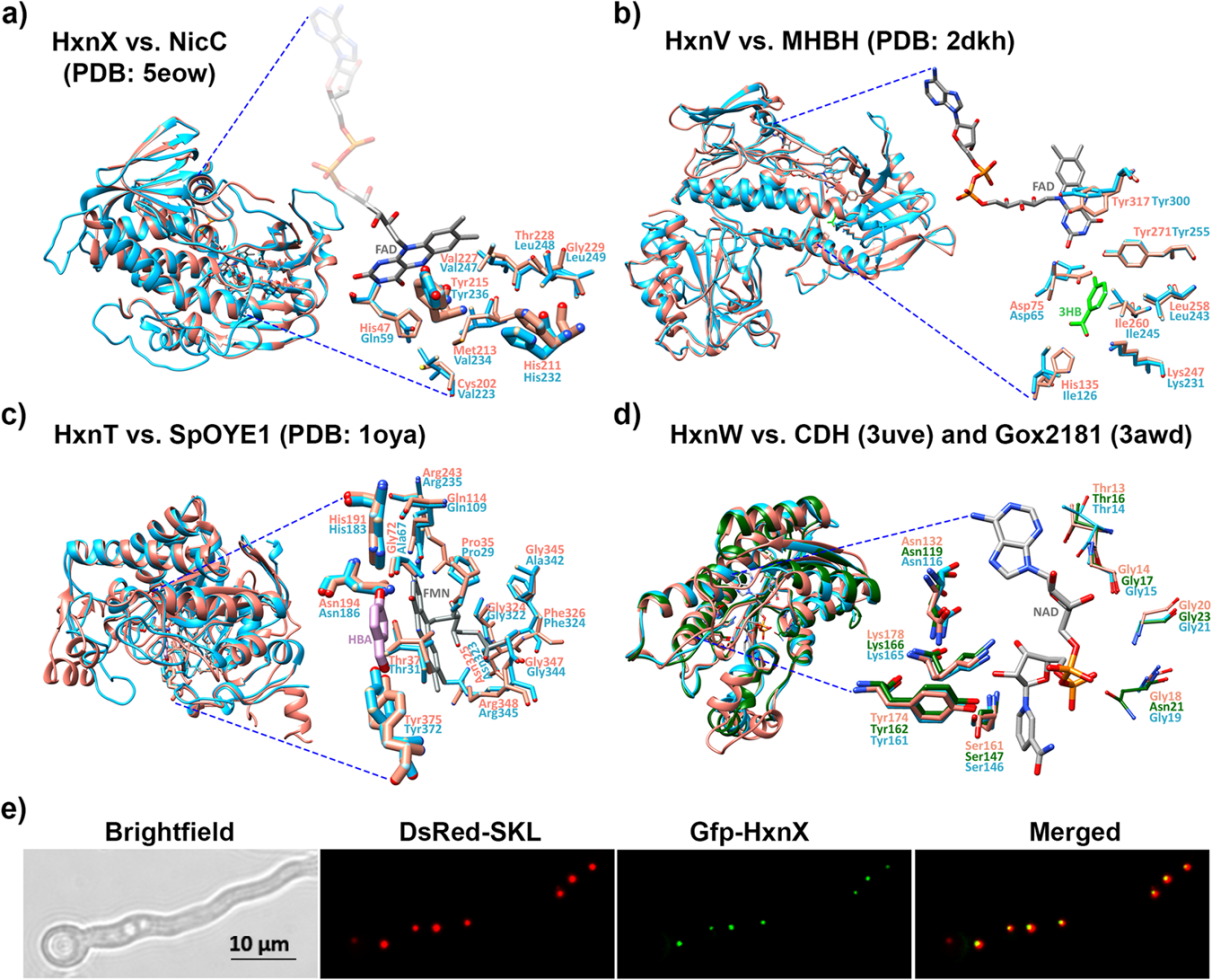
Superposition of the structural models of HxnX, HxnV, HxnT, and HxnW with their closest known structural homologs and cellular localization of HxnX. Hxn enzymes are shown in blue; the structural homologs are shown in salmon and green colors. For each model, the image to the left shows the superposition of the compared proteins in the ribbon view and includes the modeled substrate and/or cofactor. The substrate interacting side chains are shown as magnified insets in the stick view to the right of each model. FMN, FAD, and NAD cofactors are shown by gray sticks. **a** HxnX *versus* 6-NA 3-monooxygenase (NicC) from *Pseudomonas putida* (PDB code: 5eow)^18^. Thick sticks show 6-NA binding residues, while thin sticks show additional active site residues of NicC. **b** HxnV *versus* 3-hydroxybenzoate hydroxylase (MHBH) from *Comamonas testosteroni* (PDB code: 2dkh)^22^. 3HB: 3-hydroxybenzoic acid (3HB) substrate (green sticks). Sticks show the 3HB substrate-binding residues of MHBH. **c** HxnT *versus* old yellow enzyme 1 (OYE1) of *Saccharomyces pastorianus* (PDB code: 1oya)^23^. HBA: para-hydroxybenzaldehyde ligand of SpOYE1 (green sticks). Thin sticks: FMN binding residues; thick sticks: HBA binding residues of SpOYE1. **d** HxnW *versus* the polyol dehydrogenase enzyme Gox2181 from *Gluconobacter oxidans* (PDB code: 3awd) and carveol dehydrogenase CDH from *Mycobacterium avium* (PDB code: 3uve)^31,32^. Thick sticks: active site residues in Gox2181 and CDH^30–32^; thin sticks: residues of TG(X)_3_GXG NAD(P) binding motif in HxnW, characteristic of the fungal-type ketoreductases. Quality assessments of the Hxn models and superpositions with their closest known structural homologs are summarized in Supplementary Table 4. **e** Subcellular localization of the Gfp-HxnX fusion protein. Gfp-HxnX is co-expressed with DsRed-SKL (peroxisome targeted red fluorescent protein^54,55^) in strain HZS.579. Fluorescent microscopy was carried out by using Zeiss 09 and 15 filter sets for DsRed and Gfp, respectively. Conidia were germinated for 6.5 h at 37 °C on the surface coverslips submerged in MM prior to microscopy. Scale bar represents 10 μm.

HxnX is a monooxygenase (Fig. 5a). Its closest known structural homolog is the 6-NA 3-monooxygenase, NicC (PDB code: 5eow), from *Pseudomonas putida* KT2440^18^ (Fig. 5a and Supplementary Table 4). His232 and Tyr236 residues of HxnX and their spatial orientation correspond to the 6-NA substrate-binding His211 and Tyr215 residues of NicC from *P. putida* KT2440^18^ (Fig. 5a). The six additional AA residues, His47, Cys202, Met213, Val227, Thr228, and Gly229, which are involved in the formation of the active site^18^ are not conserved in HxnX (Gln59, Val223, Val234, Val247, Leu248, and Leu249, respectively) (Fig. 5a). Similarly to NicC^2,19^, HxnX is proposed to require NADH, FAD, and O_2_ to replace the carboxyl group with a hydroxyl group on the 6-NA substrate that results in 2,5-DP formation.

HxnX includes canonical PTS-1 peroxisome targeting signal (SRL) at its C-terminal end (Supplementary Fig. 2). An N-terminal Gfp-HxnX fusion fully complements the growth phenotype of *hxnXΔ* and co-localizes with a peroxisomal marker (Fig. 5e). The PTS-1 signal is conserved among the HxnX proteins present in other *Pezizomycotina*^11^. No other *hxn* encoded enzyme carries a subcellular localization signal, which however does not exclude the possibility that the corresponding pathway-step(s) may occur in an organelle.

While constructing double mutant strains, we were surprised that the *hxnSΔ hxnTΔ* double deletion strain utilizes 10 mM 6-NA more efficiently than the wild-type control or the single *hxnSΔ* or *hxnTΔ* deletion mutants (Fig. 3a). The ORFs of the two divergently transcribed genes were deleted in the double mutant, the intergenic region between the start codons was left intact, excluding any cis-acting regulatory effects on other genes of the cluster (see Methods section). The explanation of this phenotype may relate to the intracellular pool of NAD/NADH. NAD is the final electron acceptor of HxnS^8^, and the presumed electron donor of HxnT (Supplementary Fig. 3). Deletion of both cognate genes may increase the intracellular NAD/NADH pool, thus facilitating the activity of the peroxisomal HxnX, which as a monooxygenase necessitates NADH to reduce the second oxygen atom in O_2_. It seems paradoxical that the co-induction of *hxnS* and *hxnT* with *hxnX* may actually impair the utilization of 6-NA.

### Subsequent metabolism of 2,5-DP depends on the 2,5-DP monooxygenase, HxnV

N-source utilization tests showed that HxnV acts downstream of NA, 6-NA, and 2,5-DP (Fig. 3a). Induction tests (Hx Allp rows) are completely consistent with the above, in an *hxnV*∆ strain 2,5-DP does not act as an inducer. These results place the physiological inducer of the pathway downstream from 2,5-DP. In an *hxnR*^*c*^*7* background, where all other *hxn* genes are constitutively expressed^10,11^, an *hxnVΔ* strain accumulates 2,5-DP (Fig. 4a) in a medium supplemented with 10 mM 6-NA, indicating that 2,5-DP is its substrate. This strain also secretes a green pigment (detected both visually and by UHPLC-HRMS analysis), seen both in the solid medium around the colonies and in fermented broth (Fig. 4b, c). The green pigment was identified as the dimer form of 2,5-DP (Fig. 4d). A green pigment formation by a non-enzymatic transformation of 2,5-DP was reported in the *P. putida* NicX loss-of-function mutant, blocked in the catabolism of 2,5-DP^20^ and in a *P. fluorescens* strain grown on NA medium^21^. The formation of the pigment is almost completely blocked in an *hxnR*^*c*^*7 hxnX∆ hxnV∆* strain, consistent with the position of the HxnX protein in the pathway as the enzyme catalyzing the formation of 2,5-DP (see above) but also diminished in an *hxnR*^*c*^*7 hxnY∆ hxnV∆* strain. The fact that the deletion of *hxnY* diminishes the green pigment accumulation (Fig. 4c) may suggest, however, a role for HxnY in the detoxification of NA-catabolism-derived compounds.

HxnV includes a phenol 2-monooxygenase domain (PRK08294) and shows remarkable structural similarity to 3-hydroxybenzoate hydroxylase (MHBH), from *Comamonas testosteroni* (PDB code: 2dkh) (Fig. 5b) as well as to phenol 2-monooxygenase (PHOX) from *Trichosporon cutaneum* (PDB code: 1pn0) (Fig. 5b, Supplementary Fig. 4 and Supplementary Table 4). The phenol ring interacting residues of MHBH (Asp75, Leu258, Ile260, and Tyr271) together with their spatial orientation are fully conserved in HxnV (Asp65, Leu243, Ile245, and Tyr255), while the carboxyl group binding Lys247 and His135 residues of MHBH are partially conserved in HxnV (Lys231 and Ile126 in HxnV) (Fig. 5b)^22^. Thus, it is not unreasonable and in agreement with the data shown above that 2,5-DP is the substrate of HxnV, and by the analogy between HxnV and its known structural homologs, HxnV may hydroxylate the 6-carbon of 2,5-DP resulting in 2,3,6-trihydroxypyridine (2,3,6-THP) formation (Fig. 2). This metabolite was not detected in the metabolome of any of the mutants, however, the structurally identified upstream and downstream metabolites (2,5-DP and (5*S*,6*R*)-(+)-dihydroxypiperidine-2-one (see below), respectively) suggest that 2,3,6-THP is almost certainly the product of HxnV (Figs. 2,4a).

### The 2,3,6-THP alkene reductase HxnT catalyzes the reduction of the pyridine ring

Accumulation of a saturated derivative of 2,3,6-THP, (5*S*,6*R*)-(+)-dihydroxypiperidine-2-one (5,6-DHPip-2-O) was exclusively observed in the metabolome of an *hxnR*^*c*^*7 hxnWΔ* mutant (Fig. 4a and see Supplementary Table 2 for NMR results). This compound has not been detected previously in either eukaryotes or prokaryotes, and has not been synthesized chemically. 5,6-DHPip-2-O is an altogether novel compound. The accumulation pattern identifies 5,6-DHPip-2-O as the substrate of HxnW but also implies that an upstream alkene reductase enzyme (HxnT, see Fig. 2 and below) acts on the hitherto undetected product of HxnV. Logically the latter has to be 2,3,6-THP. The putative alkene reductase, which supposedly converts 2,3,6-THP to the 5,6-DHPip-2-O, is HxnT (a member of the “old yellow enzymes” group). Comparison of the structural model of HxnT with its closest known structural homolog, old yellow enzyme 1 (OYE1) of *Saccharomyces pastorianus* (PDB code: 1oya) showed that the para-hydroxybenzaldehyde binding residues of SpOYE1 (His191, Asn194, Tyr375) are remarkably conserved in HxnT (His183, Asn186, and Tyr372) and that the FMN binding residues are almost completely conserved in HxnT^23^ (Fig. 5c, Supplementary Fig. 5, and Supplementary Table 4 for further details). An *hxnT∆* strain shows a leaky growth phenotype, most noticeably on 2,5-DP and NA (Fig. 3a). The utilization of Hx in the inducer-test media is reduced but still clearly visible. Both results imply that while HxnT is responsible for the metabolism of the putative 2,3,6-THP metabolite to 5,6-DHPip-2-O, an additional unidentified enzyme must be catalyzing the same step. The deletion of *hxnW* identifies 5,6-DHPip-2-O as the physiological inducer of the pathway (NA and 6-NA serve as inducer precursors in the Hx Allp test in *hxnW∆*, but not in *hxnX∆* and to a reduced extent in *hxnT∆*). 2,5-DP serves as an inducer precursor in *hxnX∆* but not in *hxnV∆* and to a reduced extent in *hxnT∆*, which is in line with a redundantly functioning additional enzyme. While induction of a whole pathway by a metabolite such as the product of the first metabolic step has been described long ago (e.g., refs. ^24–27^ and most recent^28^ with references therein), the pathway described in this article reports the unprecedented occurrence of concerted induction by an almost terminal metabolite in a degradative, catabolic pathway (as opposed to repression by end products in biosynthetic pathways). This result implies that non-induced levels of upstream enzymes are sufficient to result in intracellular concentrations of 5,6-DHPip-2-O sufficient to act as a ligand of HxnR, resulting in positive induction feedback, similarly to what has been established for induction by uric acid of the upstream enzymes of the purine degradation pathway of *A. nidulans*^28,29^. We have purified and tested 5,6-DHPip-2-O both as a nitrogen source and as an inducer, with negative results (Supplementary Fig. 6). However, it must be stressed that only a positive result would be significant, in the absence of any independent evidence that this compound can be taken up by the cell.

### The 5,6-DHPip-2-O ketoreductase HxnW converts the 6-hydroxy group to a 6-oxo group

5,6-DHPip-2-O, the product of HxnT, is the substrate of HxnW. HxnW is a short chain dehydrogenase/reductase and has a structurally conserved NADB_Rossmann fold domain^30^ with a TG(X)_3_GXG (14-21 AAs) motif that is characteristic of the fungal ketoreductases (Supplementary Fig. 7). Comparison of HxnW to its closest known structural homologs, NAD(H)-dependent polyol dehydrogenase Gox2181 from *Gluconobacter oxydans* (PDB code: 3awd) and carveol dehydrogenase CDH from *Mycobacterium avium* (PDB code: 3uve)^31,32^ showed the striking conformity of the active site residues (Asn119, Ser147, Tyr162, and Lys166) (Fig. 5d, Supplementary Table 4). HxnW, similarly to its structural homologs, dehydrogenates a hydroxyl group of 5,6-DHPip-2-O resulting in the formation of 3-hydroxypiperidine-2,6-dione (3-HPip-2,6-DO) (Figs. 2 and 4a). Similar to 5,6-DHPip-2-O, 3-HPip-2,6-DO is also a new natural metabolite that has not been detected previously in any organism.

The 5,6-DHPip-2-O metabolite was not accumulated in a double deleted *hxnR*^*c*^*7 hxnWΔ hxnVΔ* strain, however, it was accumulated in an *hxnR*^*c*^*7 hxnWΔ hxnXΔ* strain. This implies that besides HxnX a second enzyme may be capable of metabolizing 6-NA to 2,5-DP, however, according to the growth tests of *hxnXΔ* strain, this proposed alternative activity does not support growth on its own.

### The 3-HPip-2,6-DO cyclic imide hydrolase HxnM catalyzes the opening of the piperidine ring

**Fig. 6:**
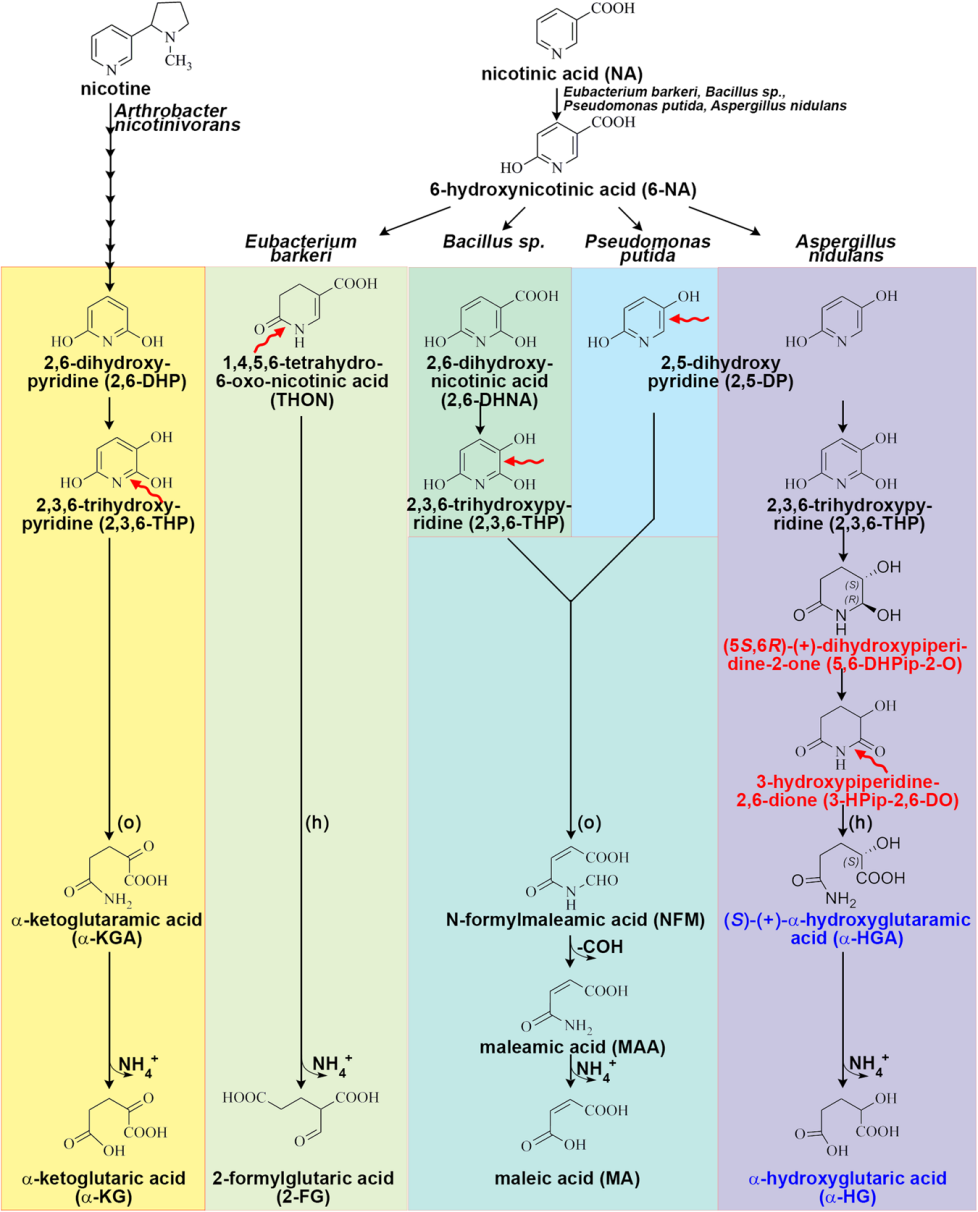
A comparison of the nicotinate catabolic pathway of the ascomycete *Aspergillus nidulans* with known prokaryotic pathways. The catabolism of nicotine by *A. nicotinovorans* involves the opening and release of the pyrrolidine ring, leading to 2,6-DP, which is further catabolized through 2,3,6-THP, an intermediate of pathways in *Bacillus* sp. as well as in *A. nidulans*. The nicotine pathway upstream to 2,6-DP (indicated by linked arrows) is not relevant to the present work. Red-colored text indicates completely novel metabolites, while blue-colored text indicates metabolites that have never been identified in prokaryotic NA catabolic pathways. While the eukaryotic NA catabolic pathway has only been studied experimentally in *A. nidulans*, genes encoding the whole or part of the pathway are present in many ascomycete fungi^11^. The site of ring-opening (either between two carbons or carbon and nitrogen) is indicated by red wavy arrows. (o): ring-opening is oxidative; (h): ring-opening is hydrolytic.

3-HPip-2,6-DO (generated by HxnW) was accumulated exclusively, albeit in small quantity, in the fermented broth of an *hxnMΔ* strain (Fig. 4a). The structure of 3-HPip-2,6-DO was deduced by the (i) exact *m*/*z* value; (ii) MS/MS fragmentation pattern of the compound obtained by UHPLC-HRMS analysis compared to the in silico fragmentation using Competitive Fragmentation Modeling-ID (CFM-ID 4.0) (Supplementary Table 1); and (iii) the UHPLC-HRMS and NMR confirmed structures of the upstream and downstream metabolites (Supplementary Tables 1, 2) in line with the proposed ketoreductase activity of the HxnW enzyme (see paragraph above). HxnM shares 74.3% identity (with 100% query coverage) with a *Candida boidinii* enzyme (OWB68015) belonging to the EC 3.5.99 enzyme class (GOterm: 0016810, hydrolase activity on non-peptide C-N bonds) and 64.2% identity (with 95.4% query coverage) with AAY98498, a cyclic imide hydrolase homolog, from *P. putida*^33^ (Supplementary Fig. 9). HxnM shows striking structural similarity with its closest known structural homolog, the so-called “peptidoglycan deacetylase” HpPgdA from *Helicobacter pylori* (PDB code: 3qbu) (its substrate specificity being unknown) that is related to cyclic imidases^34^ (Supplementary Fig. 9 and Supplementary Table 4). The closest characterized phylogenetic relative of HpPgdA is an allantoinase (PuuE) from *Pseudomonas aeruginosa*, whose natural substrate is a small cyclic imide^34,35^. We propose that HxnM opens the ring of 3-HPip-2,6-DO between a C2 carbon and nitrogen, generating (*S*)-(+)-α-hydroxyglutaramate (α-HGA), a compound which was detected exclusively in the metabolome of an *hxnR*^*c*^*7 hxnNΔ* strain (Figs. 2, 4a and Supplementary Table 2). A ring-opening is a necessary step to generate NH_4_^+^ which can serve as a nitrogen source. In *A. nidulans*, uniquely among studied NA-catabolizing organisms, the generation of a piperidine ring from a pyridine ring precedes the hydrolysis of a C-N bond. In the different pathways described in bacteria, the ring-opening may take place by an oxidative process in an aromatic ring (such as in *P. putida*) or in a hydrolytic process on saturated or partially saturated rings (*Eubacterium barkeri* and *Azorhizobium caulinodans*)^2,3,36^ (Fig. 6).

### The α-HGA amide hydrolase HxnN is involved in nitrogen salvage from NA

HxnN is a putative amide hydrolase, its closest structural homolog is the fatty acid amide hydrolase 1 (FAAH1) from *Rattus norvegicus* (Supplementary Table 4). Deletion of *hxnN* diminishes but not abolishes the utilization of NA, 6-NA and 2,5-DP as sole nitrogen sources. While *hxnN* encodes the last enzyme of the *hxn* regulon, the growth tests demonstrate that (a) yet-unidentified hydrolase(s) contribute(s) to the deamidation of α-HGA (Fig. 3a). Several genes encoding putative paralogues of HxnN are extant in the genome of *A. nidulans* with identities to HxnN up to 39%. Superposition of the structural model of HxnN with its closest known structural homolog, FAAH1 (PDB code: 2vya), shows that the catalytic triad residues from FAAH1 involved in the hydrolysis of the amide bond, the “oxyanion hole” forming residues and the Ser residue that interacts with the catalytic triad residues^37,38^ are fully conserved in HxnN (Supplementary Fig. 10 and Supplementary Table 4). None of the prokaryotic amide hydrolases operating in the bacterial NA catabolic routes (ω-amidases)^2,39,40^ show considerable similarity to HxnN. Amide hydrolysis of α-HGA generates α-hydroxyglutarate (α-HG) (Figs. 2, 4a), which has not been detected as an intermediate in any of the elucidated prokaryotic NA catabolic routes.

### Toxicity of intermediate catabolic compounds

In an *hxnR*^*c*^*7* background, all *hxn* genes are constitutively transcribed. We can thus investigate the accumulation of NA metabolites bypassing the physiological induction of the pathway. The accumulated 2,5-DP in *hxnVΔ* is a strong inhibitor of growth, while 5,6-DHPip-2-O in *hxnWΔ* mildly, and 6-NA, 3-HPip-2,6-DO, and α-HGA in *hxnXΔ*, *hxnMΔ*, and *hxnNΔ*, respectively, slightly inhibit growth (Fig. 3b). Growth inhibition by pathway metabolites was also detected when acetamide was the main N-source.

### HxnY is an α-ketoglutarate-dependent dioxygenase

Among enzymes of this class, its closest structural homolog is the thymine-7-hydroxylase (T7H) of *Neurospora crassa* (PDB code: 5c3q)^41^, which catalyzes the sequential conversion of the methyl group of thymine to a carboxyl group^41,42^. The conservation of the α-ketoglutarate and Fe^2+^ binding residues and those involved in π-π stacking and hydrophobic interactions with the pyrimidine ring of T7H are consistent with the putative activity of HxnY on a pyridine derivative related to the pathway (Supplementary Fig. 8 and Supplementary Table 4). On the basis of the *hxnYΔ*-related phenotypes, we could not propose a function for HxnY that directly relates to nicotinate catabolism. The deletion of *hxnY* diminishes both the utilization of 6-NA (Fig. 3a) and the accumulation of 2,5-DP derived green pigment (Fig. 4c). The fact that the *hxnXΔ* phenotype is not leaky (Fig. 3a), makes unlikely that HxnY contributes significantly to the NA-derived nitrogen pool by conversion of NA/6-NA to 2,5-DP. The fact that the deletion of *hxnY* diminishes the green pigment formation, however, may suggest a role in the detoxification of NA-catabolism-derived compounds.

## Conclusions

The eukaryotic NA catabolic pathway described above shows clear differences from previously described prokaryotic pathways in steps that precede (compounds 5,6-DHPip-2-O and 3-HPip-2,6-DO) and follow (compounds α-HGA and α-HG) ring-opening. Conversion of NA to 2,3,6-THP through 2,5-DP was not detected in prokaryotes, albeit each of the 2,3,6-THP and 2,5-DP intermediates appear in several pathways (2,3,6-THP appears in *Bacillus sp*. and *Arthrobacter nicotinovorans*; 2,5-DP appears in *P. putida*) (Fig. 6). 2,5-DP is formed from 6-NA in *Pseudomonas* sp., which is not hydroxylated further but the pyridine ring is cleaved between C5-C6﻿^2^ and 2,3,6-THP in *Bacillus sp*. is formed from 2,6-dihydroxynicotinic acid^43^. Notably, the formation of 2,3,6-THP occurs in the nicotine catabolism of *Arthrobacter sp*. through as a product of 2,6-dihydroxypyridine metabolism (reviewed in ref. ^44^). Steps of the saturation of the pyridine ring of 2,3,6-THP to 5,6-DHPip-2-O by the OYE-related alkene reductase HxnT (and a yet-unidentified enzyme) and oxidation of 5,6-DHPip-2-O to 3-HPip-2,6-DO by the ketoreductase/polyol dehydrogenase HxnW have hitherto only been detected in this pathway (Figs. 2, 6). Moreover, 5,6-DHPip-2-O is a completely new chemical compound. The ring-opening of the piperidine ring occurs between C-N (by the cyclic imidase HxnM) generating α-HGA, which has not been found previously in NA catabolic pathways (Fig. 6). In aerobic prokaryotic pathways the ring-opening occurs either between C-C of 2,5-DP (by extradiol dioxygenase) or 2,3,6-THP (in *Pseudomonas sp*. and *Bacillus sp*., respectively) or between C-N of 2,3,6-THP (in *Rhodococcus sp*. and *Arthrobacter sp*. by polyketide cyclase) generating N-formyl maleamic acid or α-ketoglutaramate^2,39,40,43^ (Fig. 6). In the following steps in prokaryotes, the amide is hydrolyzed by an ω-amidase^2,39,40^ not related to the HxnN amidase. The anaerobic pathway described in *E. barkeri* and *Azorhizobium caulinodans* involves the partial saturation of the pyridine ring of 6-NA that results in 1,4,5,6-tetrahydro-6-oxonicotinic acid (THON), followed by hydrolytic ring-opening of THON between C-N and the simultaneous deamination (by a bifunctional enamidase in *E. barkeri*) resulting in (*S*)-2-formylglutarate formation^3,36^ (Fig. 6). While no redundantly functioning enzymes are involved in the prokaryotic routes, two steps of the fungal catabolism involve alternative enzymes (two unidentified enzymes, one functioning redundantly with HxnT, the other with HxnN) (Fig. 2). Catabolic steps downstream to 2,3,6-THP differ from those in prokaryotes and lead to the newly identified intermediate metabolites 5,6-DHPip-2-O and 3-HPip-2,6-DO (Fig. 6). The identification of these new metabolites may be of industrial or agricultural importance. The complete description of this eukaryotic pathway further illustrates convergent evolution, both at the level of individual enzymes and at the level of a whole pathway.

## Methods

### Strains and growth conditions

The *A. nidulans* strains used in this study are listed in Supplementary Table 3. Standard genetic markers are described in http://www.fgsc.net/Aspergillus/gene_list/. Minimal media (MMs) with glucose as the sole carbon source and different sole nitrogen sources were used^45,46^. The media were supplemented with vitamins (http://www.fgsc.net) according to the requirements of each auxotrophic strain. Nitrogen sources, inducers, repressors, and inhibitors were used at the following concentrations: 10 mM NA or 10 mM 6-NA (1:100 dilution from 1 M NA or 6-NA dissolved in 1 M sodium hydroxide), 10 mM 2,5-DP added as a powder, 10 mM NAA added as a powder, 10 mM 5,6-DHPip-2-O added as a powder, 1 mM Hx added as a powder, 10 mM acetamide as sole N-sources; NA sodium salt, 6-NA sodium salt, 2,5-DP, NAA, 5,6-DHPip-2-O in 1 mM or 100 µM final concentration as inducers; 5.5 µM Allp as an inhibitor of purine hydroxylase I (HxA) enzyme activity. Strains were grown at 37 °C for the indicated times.

For metabolite extraction, the mycelia of *hxnR*^*c*^*7* strains with different *hxn* gene deletion(s) were grown for 16 h on MM with 10 mM acetamide as the sole N-source at 37 °C with 150 rpm agitation, which was followed by shifting the mycelia to MM with 10 mM 6-NA as substrate without additional utilizable N-source and incubated for further 24 h.

### Gene deletions

Deletion of *hxnT/R/Y/Z/P/X/W/V/M/N* genes were constructed as described previously^47^. The gene targeting substitution cassette was constructed by double-joint PCR^48^, where the *riboB*^*+*^, *pabaA*^*+*^, or *pyroA*^*+*^ genes were used as transformation markers. Construction of double and triple deletion mutants or changing the *hxnR*^*+*^ genetic background of mutants to *hxnR*^*c*^*7* was carried out by standard genetic crosses or transformation followed by checking via PCR and Southern blots. DNA was prepared from *A. nidulans* as described by ref. ^49^. Hybond-N membranes (Amersham/GE Healthcare) were used for Southern blots^50^. Southern hybridizations were done by DIG DNA Labeling and Detection Kit (Roche) according to the manufacturer’s instructions. Transformations of *A. nidulans* protoplasts were performed as described by ref. ^51^. The protoplasts were prepared from mycelia grown on cellophane^52,53^ using a 4% solution of Glucanex (Novozymes, Switzerland) in 0.7 M KCl. Transformation of 5 × 10^7^ protoplasts was carried out with 100–500 ng of fusion PCR products. Primers used in the manipulations described above are listed in Supplementary Table 5. For a detailed description of single and multiple gene deletions see Supplementary Methods 1,2.

### Construction and microscopy of Gfp-HxnX (N-terminal fusion) expressing strains

Construction of the *gfp*-*hxnX* expressing strain is described in detail in Supplementary Methods 3. Briefly, a bipartite cassette of the *gfp*-*hxnX* fusion was constructed by double-joint PCR (DJ-PCR)^48^, and cloned into the pAN-HZS-1 vector^47^ yielding the *gfp*-*hxnX* expression vector pAN-HZS-13, which was used to transform an *hxnXΔ* strain (HZS.534), which carries a peroxisome marker (expresses DsRed-SKL)^54,55^ (Supplementary Methods 3). Transformants carrying the *gfp-hxnX* transgene from one to ten copies were isolated. Gfp-HxnX localization was studied in HZS.579 that carried the transgene in seven copies. Conidiospores of HZS.579 was germinated for 6.5 h on the surface of coverslips submerged in MM at 37 °C. Young hyphae were examined by fluorescence microscopy using Zeiss 09 and 15 filter sets for DsRed and GFP, respectively.

### Metabolite analysis

For metabolite extraction, 1 ml of methanol/water (8/2) was added to both 25 mg of freeze-dried mycelium and 2 ml freeze-dried fermentation broth from each cultivation followed by vortexing for 1 min and sonication at 50 W for 3 × 5 min on ice in between vortexing the samples for 30 s. After centrifugation (20,000×*g*, 10 min, 4 °C) the supernatants were subjected to UHPLC-HRMS analysis. UHPLC-HRMS measurements were performed using a DionexUltimate 3000 UHPLC system (Thermo Scientific) coupled to a Q Exactive Plus hybrid quadrupole-Orbitrap mass spectrometer (Thermo Scientific) operating with a heated electrospray interface (HESI). Metabolites were separated on an Acquity UPLC BEH Amide (2.1 × 100 mm, 1.7 μm) column (Waters, Hungary) thermostated at 40 °C. Acetonitrile (A) and water (B) both supplemented with 0.1% formic acid served as mobile phases. A gradient elution program was applied as follows: 0–0.5 min: 97% A, 0.5–4 min: 97–88% A, 4–10 min: 88–40% A, 10–13 min: 40% A, 13–13.5 min: 40–97% A, 13.5–27.5 min: 97% A. The flow rate was kept at 0.3 ml/min, and the injection volume was 3 µl.

All samples were analyzed in both positive and negative ionization mode using the following ion source settings: the temperature of the probe heater and ion transfer capillary, spray voltage, sheath gas flow rate, auxiliary gas flow rate, and S-lens RF level were set to 300 °C, 350 °C, 3.5 kV, 40 arbitrary unit, 10 arbitrary unit, and 50 arbitrary unit, respectively. For data acquisition full-scan/data-dependent MS/MS method (Full MS/ddMS2) was applied, where the full scan MS spectra were acquired at a resolution of 70,000 from *m/z* 50 to 500 with a maximum injection time of 100 ms. For every full scan, five ddMS2-scans were carried out with a resolution of 17,500 and a minimum automatic gain control target of 1.00 × 10^5^. The isolation window was 0.4 *m/z*. Instrument control and data collection were carried out using Trace Finder 4.0 (Thermo Scientific) software. The raw data files were processed by Compound Discoverer 2.1 software for chromatographic alignment, compound detection, and accurate mass determination.

All NMR experiments were accomplished on a Bruker Ultrashield 500 Plus spectrometer, solvent residual signals (methanol, DMSO) adopted as internal standards. Optical rotations were measured with a Jasco P 2000 Polarimeter.

### Purification of 5,6-DHPip-2-O and α-HGA

About 4 and 14 g of freeze-dried mycelia of 5,6-DHPip-2-O and α-HGA accumulating strains were extracted in 160 and 560 ml of methanol, respectively. The extracts were then evaporated to dryness and were purified with dry sample loading injection on a CombiFlash EZPrep flash chromatograph (Teledyne Isco, USA) using 0.063–0.2 mm spherical silica (Molar Chemicals, Hungary) as solid phase. The metabolite detected at *m/z* 132.0656 was separated with ethyl acetate/methanol, 4/1 (V/V) supplemented with 5% aqueous ammonia as a mobile phase resulting in 5 mg material. For the metabolite detected at *m/z* 146.0461, the separation using ethyl acetate/methanol, 7/3 (V/V) supplemented with 5% aqueous ammonia was followed by an additional separation step, where a mixture of methanol/water (95/5, V/V) as mobile phase was applied to achieve 6 mg purified material. At each step of the purification, the purities of the metabolites were determined via the UHPLC-HRMS method described above.

To assign the stereochemistry of the isolated α-HGA, (*S*)-(+)- and (*R*)-(–)-α-HGA were prepared from commercially available (*S*)-(+)- and (*R*)-(–)-5-oxo-2-tetrahydrofurancarboxylic acid (Merck Co.) following literature^56^ and measuring optical rotations. The observed optical rotation of isolated α-HGA (${[{{{{{\rm{\alpha }}}}}}]}_{{{{{{\rm{D}}}}}}}^{20}$ + 7.8°, c = 0.077, MeOH) clearly proved the presence of (*S*)-(+)-α-HGA (^57^: ${[{{{{{\rm{\alpha }}}}}}]}_{{{{{{\rm{D}}}}}}}^{20}$ + 1.2°, c = 2.5, H_2_O; measured of the compound prepared from (*S*)-(+)-5-oxo-2-tetrahydrofurancarboxylic acid: ${[{{{{{\rm{\alpha }}}}}}]}_{{{{{{\rm{D}}}}}}}^{20}$ + 6.3°, c = 0.076, MeOH). Since the NOESY measurement and coupling constants of H-5 and H-6 in 5,6-DHPip-2-O clearly show trans relative configuration and because the configuration of H-5 is the same as in α-HGA, the stereochemistry of 5,6-DHPip-2-O was determined as 5*S*,6*R*. Optical rotation of 5*S*,6*R*-DHPip-2-O was: ${[{{{{{\rm{\alpha }}}}}}]}_{{{{{{\rm{D}}}}}}}^{20}$ + 51.6°, c = 0.133, MeOH.

### In silico structural analysis of Hxn proteins

Structural models of the Hxn enzymes were obtained with I-Tasser^58^ followed by refining the models using ModRefiner^59^ and Ramachandran plot quality assessment (results of the model- and superpositioning quality assessments are summarized in Supplementary Table 4). The result of I-Tasser analysis^58^ provided a list of structural homologs, those with the best C-score were chosen to superpose with the refined models.

### Reporting summary

Further information on research design is available in the Nature Research Reporting Summary linked to this article.

## Supplementary information

Supplementary Information

Reporting Summary

## Data Availability

All experimental data are shown in either the main text or in the Supplementary files (Supplementary Figures, Supplementary Tables, and Supplementary Methods).
