# Supplementary material for: A complete nicotinate degradation pathway in the microbial eukaryote Aspergillus nidulans
Source: Commun Biol. 2022 Jul 21;5:723. doi: 10.1038/s42003-022-03684-3 (PMC9304392; doi:10.1038/s42003-022-03684-3)
Supplement: Supplementary file 1 — Supplementary Information [file 42003_2022_3684_MOESM1_ESM.pdf]

SUPPLEMENTARY INFORMATION

for

A complete nicotinate degradation pathway in the microbial eukaryote *Aspergillus nidulans*

Eszter Bokor[1], Judit Ámon[1], Mónika Varga[1], András Szekeres[1], Zsófia Hegedűs[1], Tamás Jakusch[2], Zsolt Szakonyi[3], Michel Flipphi[4], Csaba Vágvölgyi[1], Attila Gácser[5,6], Claudio Scazzocchio[7,8]* and Zsuzsanna Hamari[1]*
[1]University of Szeged Faculty of Science and Informatics, Department of Microbiology, Szeged, Hungary
[2]University of Szeged Faculty of Science and Informatics, Department of Inorganic and Analytical Chemistry, Szeged, Hungary
[3]University of Szeged Faculty of Pharmacy, Institute of Pharmaceutical Chemistry, Szeged, Hungary
[4] Institute de Génétique et Microbiologie, Université Paris-Sud, Orsay, France
[5] HCEMM-USZ Fungal Pathogens Research Group, University of Szeged Faculty of Science and Informatics, Department of Microbiology, Szeged, Hungary
[6] MTA-SZTE "Lendület" Mycobiome Research Group, University of Szeged, Szeged, Hungary
[7] Section of Microbiology, Department of Infectious Diseases, Imperial College, London, United Kingdom
[8] Université Paris-Saclay, CEA, CNRS, Institute for Integrative Biology of the Cell (I2BC), 91198, Gif-sur-Yvette, France
Present address of M.F.: Department of Biochemical Engineering, Faculty of Science and Technology, University of Debrecen, Debrecen, Hungary
* Corresponding authors:
hamari@bio.u-szeged.hu,
c.scazzocchio@imperial.ac.uk

Content:

Supplementary Figures (1-10)

Supplementary Tables (1-5)

Supplementary Methods (1-3)

SUPPELEMENTARY FIGURES
for

A complete nicotinate degradation pathway in the microbial eukaryote *Aspergillus nidulans*

Eszter Bokor[1], Judit Ámon[1], Mónika Varga[1], András Szekeres[1], Zsófia Hegedűs[1], Tamás Jakusch[2], Zsolt Szakonyi[3], Michel Flipphi[4], Csaba Vágvölgyi[1], Attila Gácser[5,6], Claudio Scazzocchio[7,8]* and Zsuzsanna Hamari[1]*

[1]University of Szeged Faculty of Science and Informatics, Department of Microbiology, Szeged, Hungary

[2]University of Szeged Faculty of Science and Informatics, Department of Inorganic and Analytical Chemistry, Szeged, Hungary

[3]University of Szeged Faculty of Pharmacy, Institute of Pharmaceutical Chemistry, Szeged, Hungary

[4] Institute de Génétique et Microbiologie, Université Paris-Sud, Orsay, France

[5] HCEMM-USZ Fungal Pathogens Research Group, University of Szeged Faculty of Science and Informatics, Department of Microbiology, Szeged, Hungary

[6] MTA-SZTE "Lendület" Mycobiome Research Group, University of Szeged, Szeged, Hungary

[7] Section of Microbiology, Department of Infectious Diseases, Imperial College, London, United Kingdom

[8] Université Paris-Saclay, CEA, CNRS, Institute for Integrative Biology of the Cell (I2BC), 91198, Gif-sur-Yvette, France

Present address of M.F.: Department of Biochemical Engineering, Faculty of Science and Technology, University of Debrecen, Debrecen, Hungary

* Corresponding authors:
hamari@bio.u-szeged.hu,
c.scazzocchio@imperial.ac.uk

Content:

Supplementary Fig. 1: Transmembrane topologies of the HxnP and HxnZ transporters and expression profile of AN5650, the closest homolog of the yeast nicotinate transporter TNA1 in *Aspergillus nidulans*.

Supplementary Fig. 2: HxnX shares structural similarities with the 6-NA 3-monooxygenase, NicC of *Pseudomonas putida*.

Supplementary Fig. 3: Enzyme staining of HxnT with NADH as an electron donor in polyacrylamide gels confirms that HxnT is a FMN flavin-oxidoreductase.

Supplementary Fig. 4: Structural comparison of HxnV and the phenol hydroxylase enzyme PHOX from *Trichosporon cutaneum*.

Supplementary Fig. 5: Comparison of HxnT to old yellow enzymes.

Supplementary Fig. 6: Utilization and inducer tests of the purified 5,6-DHPip-2-O on wild type and *hxnR^{c}7* strains.

Supplementary Fig. 7: Comparison of the predicted secondary structure of HxnW with the secondary structure of Gox2181 from *Gluconobacter oxydans*.

Supplementary Fig. 8: Comparative *in silico* analysis of HxnY.

Supplementary Fig. 9: Comparison of HxnM to its homologs.

Supplementary Fig. 10: HxnN shares structural similarities with the Fatty-acid amide hydrolase 1, FAAH, from *Rattus norvegicus*.

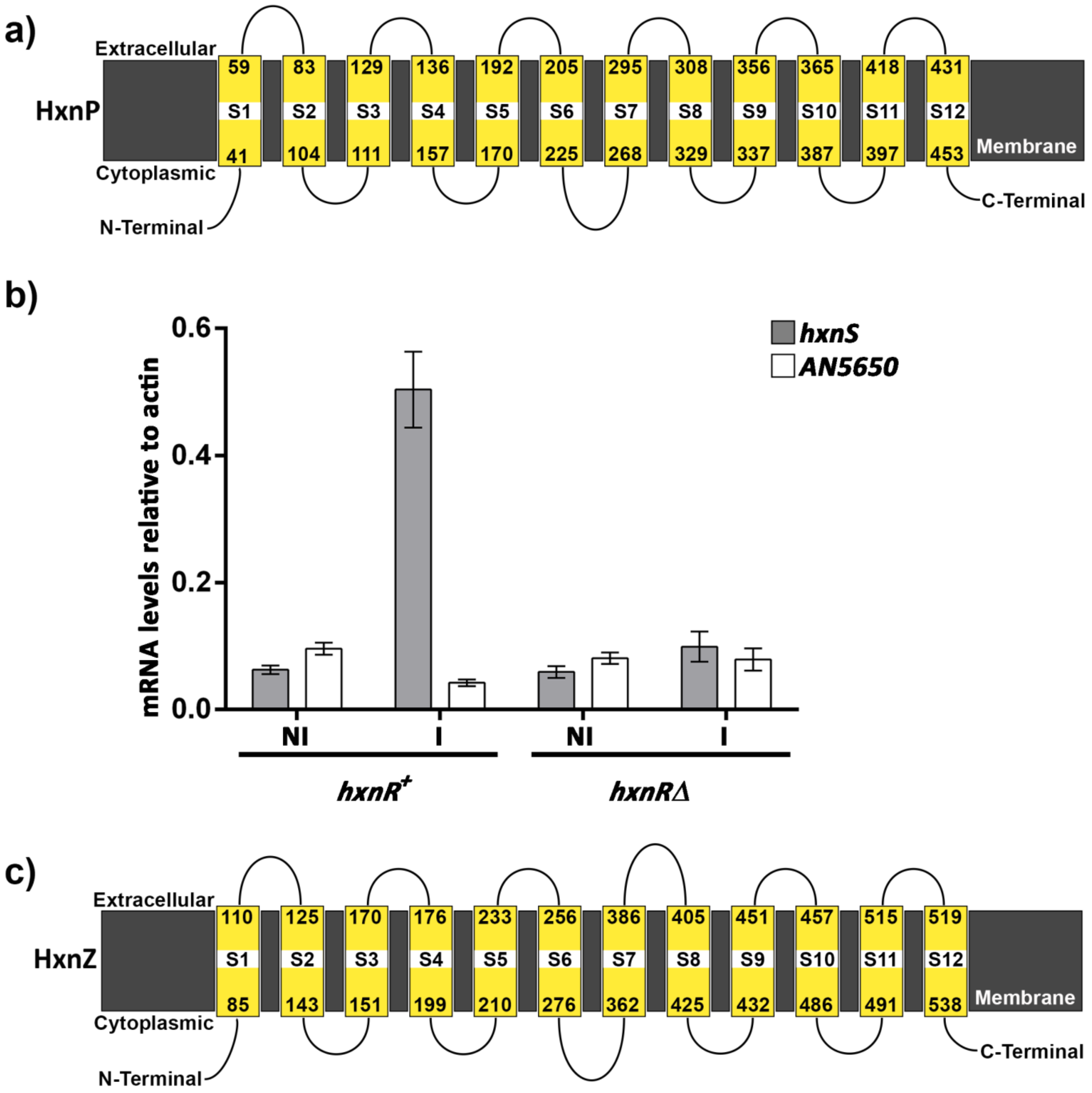

Supplementary Fig. 1. Transmembrane topologies of the HxnP and HxnZ transporters and expression profile of AN5650, the closest homolog of the yeast nicotinate transporter TNA1 in *Aspergillus nidulans*.

a) Secondary structure of HxnP obtained with Phyre2 [1]. HxnP is predicted to be a 12-segment transmembrane protein of the Major Facilitator Superfamily (PF07690.13). The nearest characterized homologue of HxnP is TNA1 (shares 24% identity with HxnP), the NA transporter of *S. cerevisiae* [2]. However, the most likely orthologue of TNA1 in *A. nidulans* (encoded by AN5650 and shares 31% identity with TNA1) and also its paralogues in the genome show higher similarity with TNA1 than HxnP. This may signify a divergence in substrate specificity and/or a redundancy of nicotinate transporters. While *hxnP* shows a

pattern of regulation identical to that of *hxnS* and the other enzyme-encoding genes of the clusters [3], expression of AN5650 is completely independent from HxnR and NA or 6-NA induction (see panel **b**). Additionally, RNAseq data [4,5] indicates that AN5650 is equally expressed on complete medium as in conditions of nitrogen starvation, which confirms that AN5650 is not related to NA utilization.

b) Gene expression analysis of AN5650. The mRNA levels were measured by RT-qPCR and data were processed according to the relative standard curve method [6] with γ-actin (*actA*) as reference mRNA. Mycelia were grown on 10 mM acetamide as sole N-source for 8 hours at 37 °C. They were either maintained on the same media for a further 2 hours (non-induced, NI) or induced with 1 mM NA (as the sodium salt, I). Used strains were *hxnR*$^{+}$ (FGSC A26) and *hxnR*Δ (HZS.614). Standard errors of three independent experiments are shown in all RT-qPCR. Primers 152-153 (for *actA*), 158-159 (for *hxnS*) and 160-161 (for AN5650) are listed in Supplementary Table 5.

c) Secondary structure of HxnZ obtained with Phyre2 [1]. HxnZ is a predicted transporter of the MFS_1 superfamily with 12-segment transmembrane domain. Its closest characterized homolog in *S. cerevisiae* (17% identity) is PHO84, a high-affinity phosphate transporter.

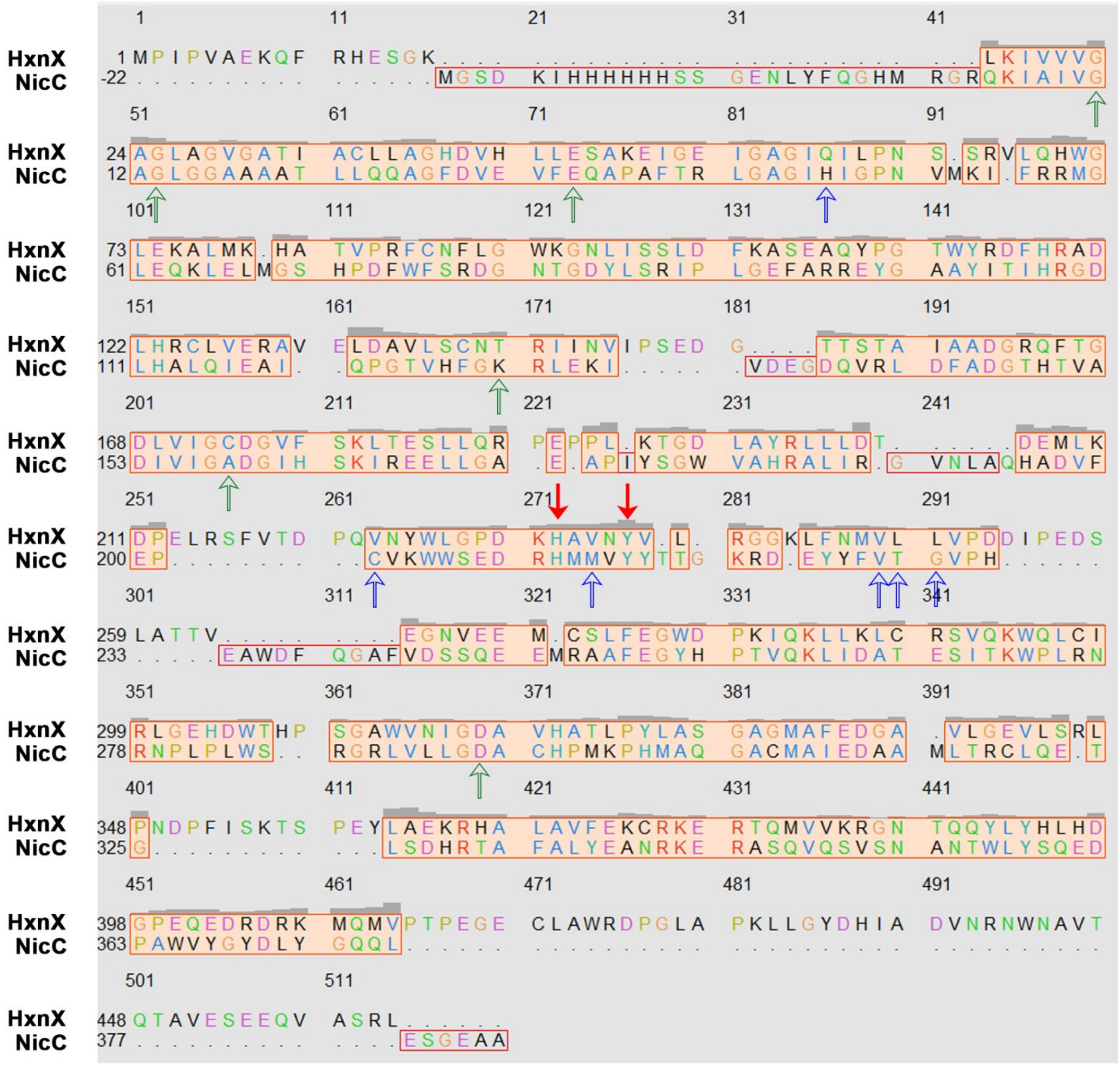

Supplementary Fig. 2. HxnX shares structural similarities with the 6-NA 3-monooxygenase, NicC of *Pseudomonas putida*.

Alignment of the superimposed proteins shown in Fig. 5a was conducted by Matchmaker Match Align within Chimera. Solid red arrows indicate the substrate binding residues in NicC; empty red arrows indicate additional AA residues that were mapped to the active centre in NicC; green empty arrows mark the FAD binding residues in NicC [7].

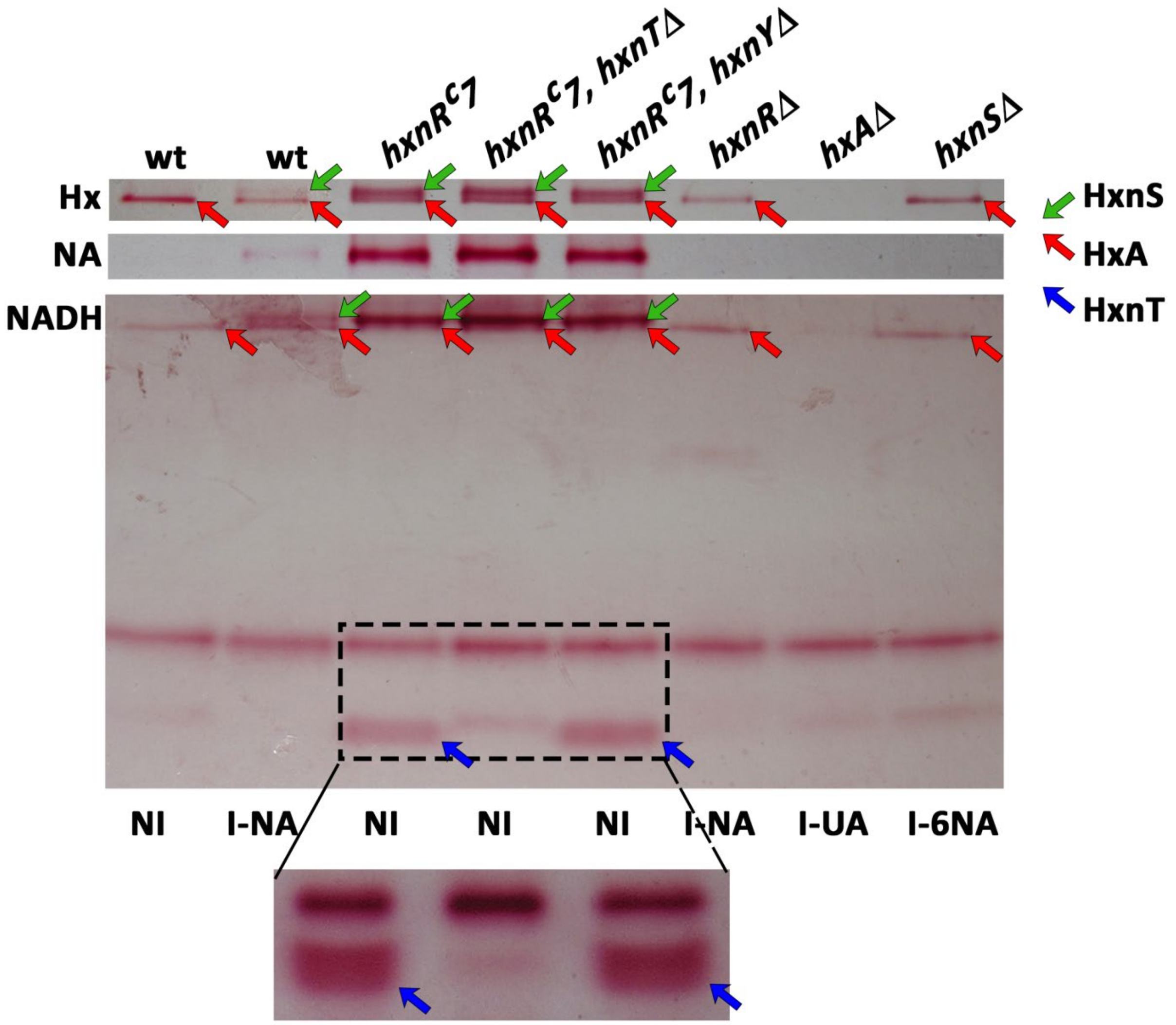

Supplementary Fig. 3. Enzyme staining of HxnT with NADH as an electron donor in polyacrylamide gels confirms that HxnT is a FMN flavin-oxidoreductase.

We have previously shown that both HxA [8](Scazzocchio, 1973) and HxnS [9] can transfer electrons from NADH to tetrazolium. This is shown in the NADH stained native 10% PAGE, where HxnS is shown by an NA inducible band (green arrows) in the wild type (wt) and a strong constitutive band in all *hxnR^c7* strains, while a basal level of HxA is shown in all non-induced conditions (without uric acid induction), except in *hxAΔ* strain, from which HxA is missing (red arrows). Two additional, constitutive NADH oxidoreductase bands are seen in all strains. Just below the higher mobility band, a faint staining band, a new band is seen in *hxnR^c7* strains, but not in the *hxnR^c7 hxnTΔ* strains (blue arrows). Samples in the boxed area were reloaded in 6-18 % gradient acrylamide gel (shown below), where the separation of the HxnY specific band from the constitutive band was clearer. NI, non-induced, I-NA, induced with nicotinic acid, I-UA induced with uric acid (which induces HxA but not HxnS), I-6NA, induced with 6-hydroxynicotinic acid. Strains: wt (HZS.145); *hxnR^c7* (FGSCA872); *hxnR^c7*

hxnTΔ (HZS.427); *hxnRΔ* (HZS.614); *hxAΔ* (HZS.245); *hxnSΔ* (HZS.254). Strains were grown on 10 mM acetamide N-source for 20 hours (NI) or 1 mM NA, 1 mM 6-NA or 0.6 mM UA was added to the media at 15 hours for induction. Green, red and blue arrows mark HxnS, HxA and HxnT, respectively.

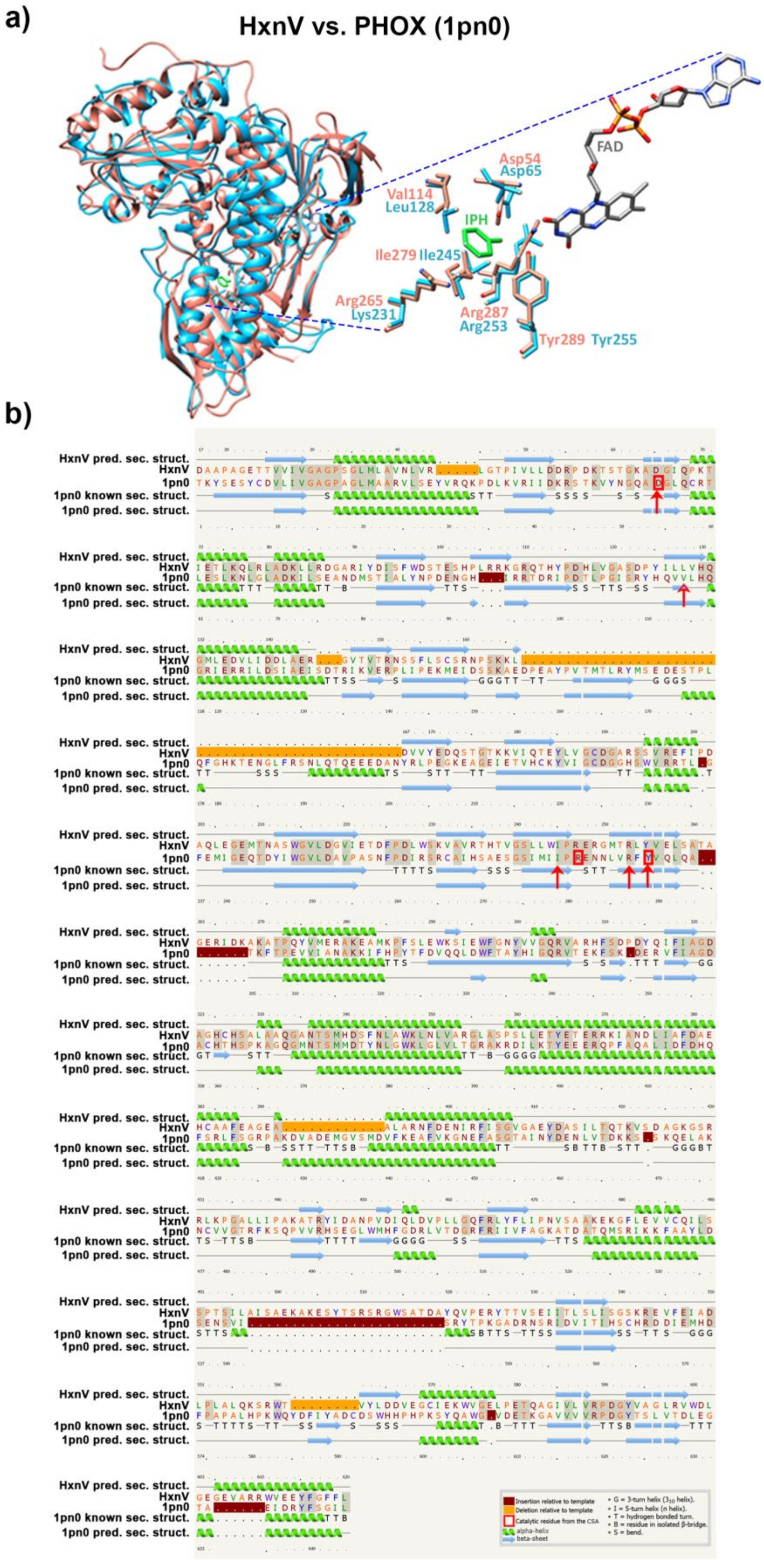

Supplementary Fig. 4. Structural comparison of HxnV and the phenol hydroxylase enzyme PHOX from *Trichosporon cutaneum*.

a) Superposition of HxnV with PHOX (PDB code: 1pn0) shows that functionally important residues are conserved in HxnV. Salmon color shows PHOX, blue color shows HxnV. The

Tyr289, Ile279 and Val114 residues of PHOX (Tyr255, Ile245 and Leu128 in HxnV, respectively) establish hydrophobic interaction with the 2- and 6-carbons of the phenol ring, while Tyr289 and Asp54 (Tyr255 and Asp65 in HxnV) form hydrogen bonds with the hydroxyl group of the phenol molecule (IPH, green sticks). Tyr289 (Tyr255 in HxnV) also directly interacts with FAD [10,11]. The Arg287 and Arg265 in PHOX (Arg253 and Lys231 in HxnV) are part of the active site through their interactions with Ile279 and Tyr289 (Ile245 and Tyr255 in HxnV). For quality assessment of the structural model of HxnV, see Supplementary Table 4.

b) The predicted secondary structure of HxnV is shown in comparison both to the known and the predicted secondary structures of PHOX (PDB code: 1pn0) from *T. cutaneum*. Figure was obtained with Phyre2 [1]. Red arrows indicate the Phenol-binding AA residues.

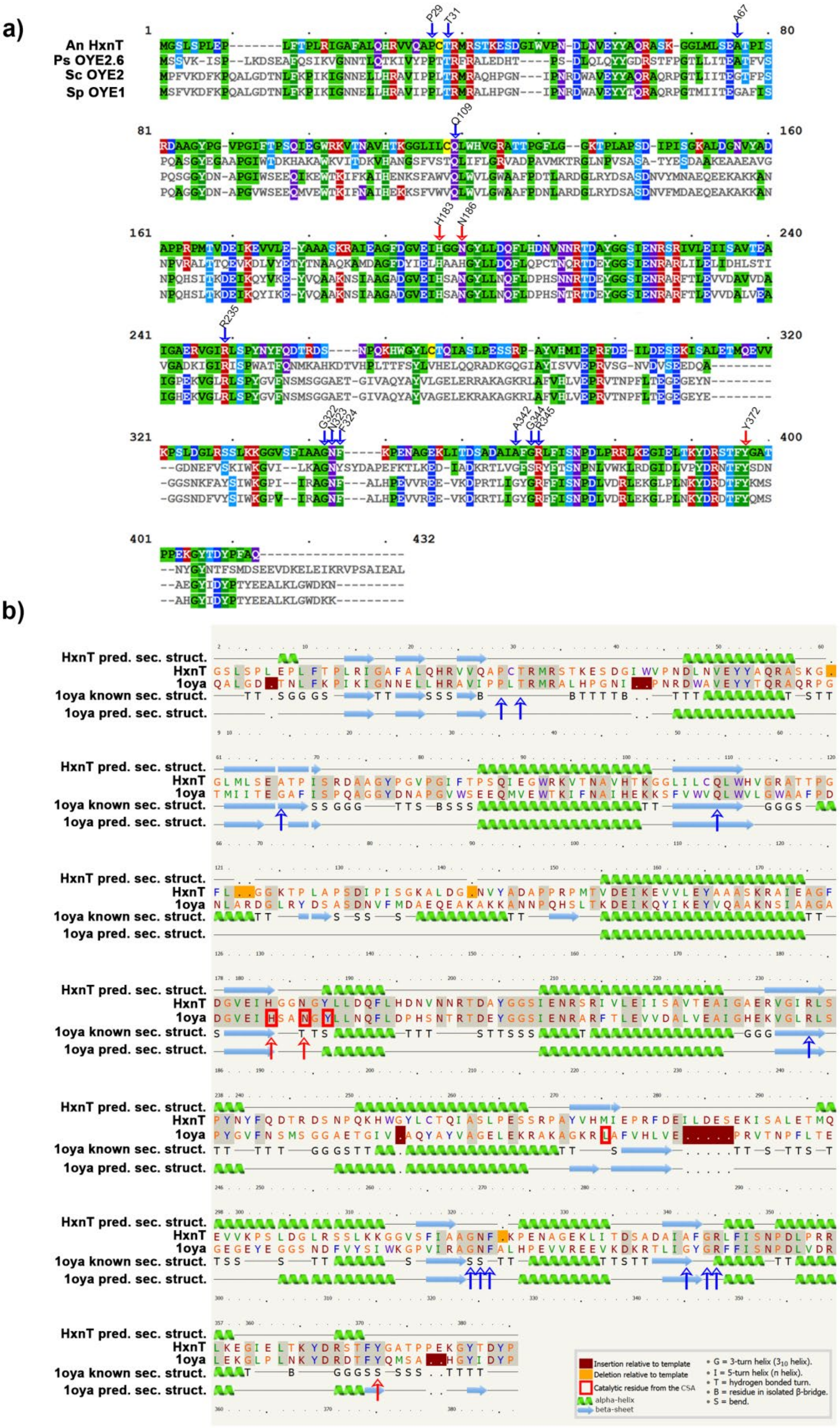

Supplementary Fig. 5. Comparison of HxnT to old yellow enzymes.

a) Alignment of the primary structure of HxnT with its nearest known homolog enzymes belonging to the OYE-like alkene reductases: PsOYE2.6 from *Pichia stipitis* (XP_001384055; 31.9% identity) [12], ScOYE2 from *Saccharomyces cerevisiae* (AAB68024; 36,8% identity) [13]

and SpOYE1 from *Saccharomyces pastorianus* (Q02899; 40.7% identity) [14]. Alignment was carried out with Muscle, and visualised with MView. Red and blue arrows indicate equivalent residues of the substrate- and FMN binding sites in HxnT, respectively. FMN binding sites are highly conserved in HxnT. Full conservation of the substrate binding residues of SpOYE1 and ScOYE2 to the corresponding AA residues of HxnT (His/Asn/Tyr AAs numbered as 191/194/375 in SpOYE1, 192/195/376 in ScOYE2 and 183/186/372 in HxnT, respectively) indicates functional similarities of HxnT and ScOYE2 [13] and SpOYE1 [14] rather than with PsOYE2.6 [12]. The His/Asn/Tyr triad was shown to establish hydrogen bonds with the nicotinamide moiety of NAD(P)H, which while is quite unusual for most NAD(P)H dependent enzymes is typical of the OYE family [12,14]. *In vitro* NADPH-tetrazolium enzyme assay on *hxnR^{c}7 hxnT$^+$* as control and *hxnR^{c}7 hxnTΔ* strains (see Supplementary Fig. 3) verified that HxnT can transfer electrons from NADH to tetrazolium, thus HxnT can acts as an FMN flavin-oxidoreductase.

b) The predicted secondary structure of HxnT is shown in comparison to both the known and predicted secondary structures of SpOYE1 from *S. pastorianus* (PDB: 1oya). The image was obtained with Phyre2 [1]. Red and blue arrows indicate the substrate binding and FMN binding AA residues in SpOYE1 as it is shown in panel a.

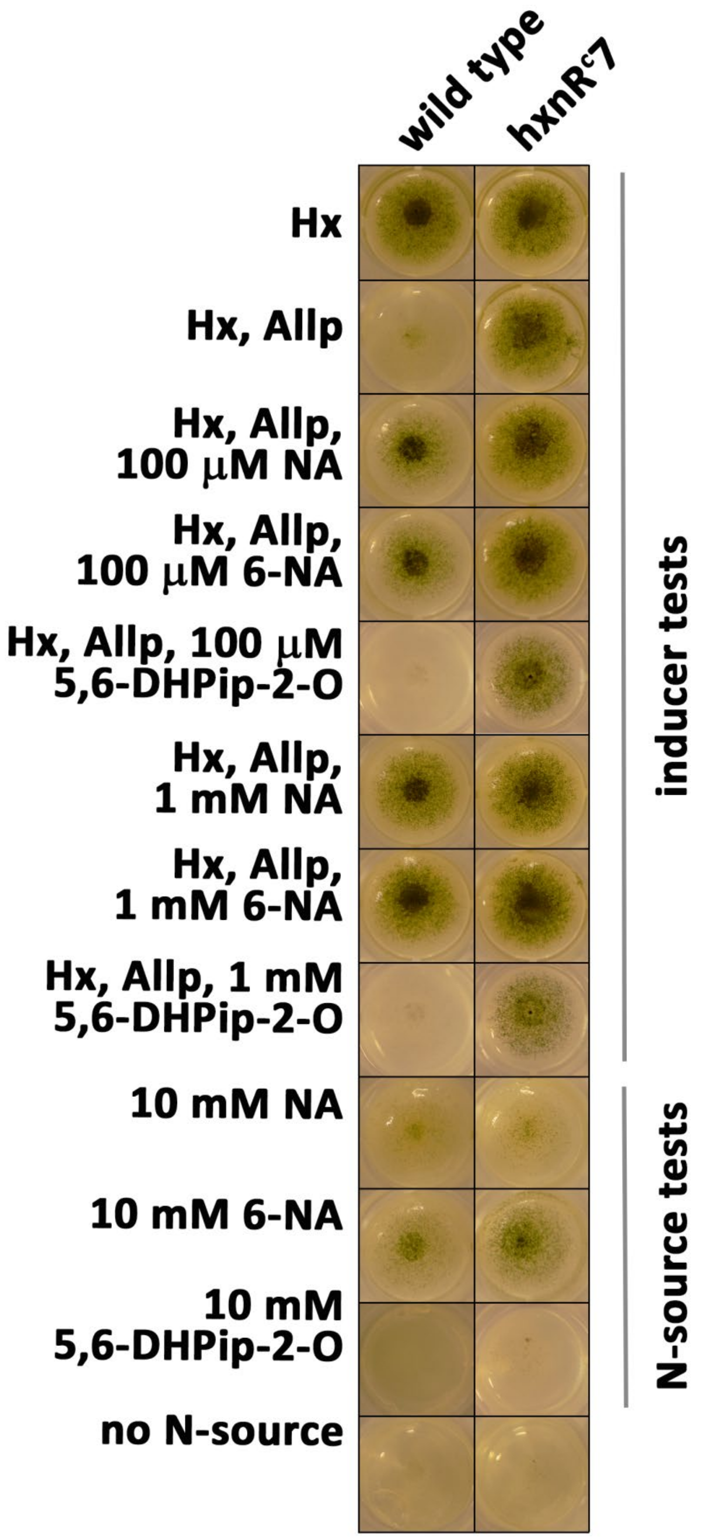

Supplementary Fig. 6. Utilization and inducer tests of the purified 5,6-DHPip-2-O on wild type and *hxnR^{c}7* strains.

Utilization of different nitrogen sources by *hxnR^{+}* wild type and *hxnR^{c}7* constitutive mutant strain. Above the columns we indicate the tested strains. Hx indicates 1 mM hypoxanthine as the sole nitrogen source. Hx, Allp, as above including 5.5 μM allopurinol, which fully inhibits

HxA but not HxnS (therefore Hx utilization depends on the activation of HxnR-regulon-belonging HxnS (for details see [3]). NA and 6-NA indicate, respectively, nicotinic acid and 6-OH nicotinic acid added as the sodium salts (see Methods section). 5,6-DHPip-2-O indicates (5*S*,6*R*)-(+)-dihydroxypiperidine-2-one purified from *hxnR^{c}7 hxnWΔ* mutant. Other relevant concentrations are indicated in the figure. Plates were incubated for 3 days at 37 °C. Strains used: wild type strain (HZS.120) and *hxnR^{c}7* (FGSC A872). The complete genotypes are given in Supplementary Table 3.

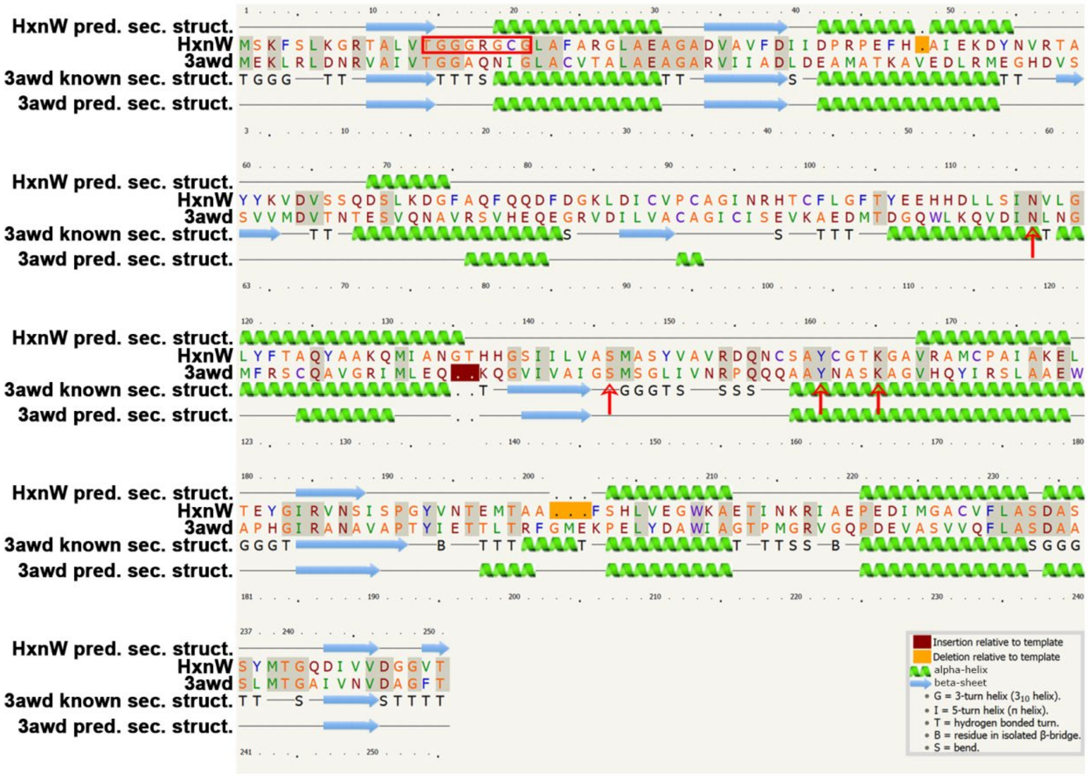

Supplementary Fig. 7. Comparison of the predicted secondary structure of HxnW with the secondary structure of Gox2181 from *Gluconobacter oxydans*.

The closest known structural homolog of HxnW is the polyol dehydrogenase enzyme Gox2181 from *G. oxydans* (PDB code: 3awd) [15]. The comparative analysis and the image was obtained with Phyre2 [1]. Boxed residues show the TG(X)$_3$GXG NAD(P)-binding motif characteristic to the fungal type ketoreductases. Red arrows indicate the conserved catalytic tetrad of the NADB_Rossmann fold domain.

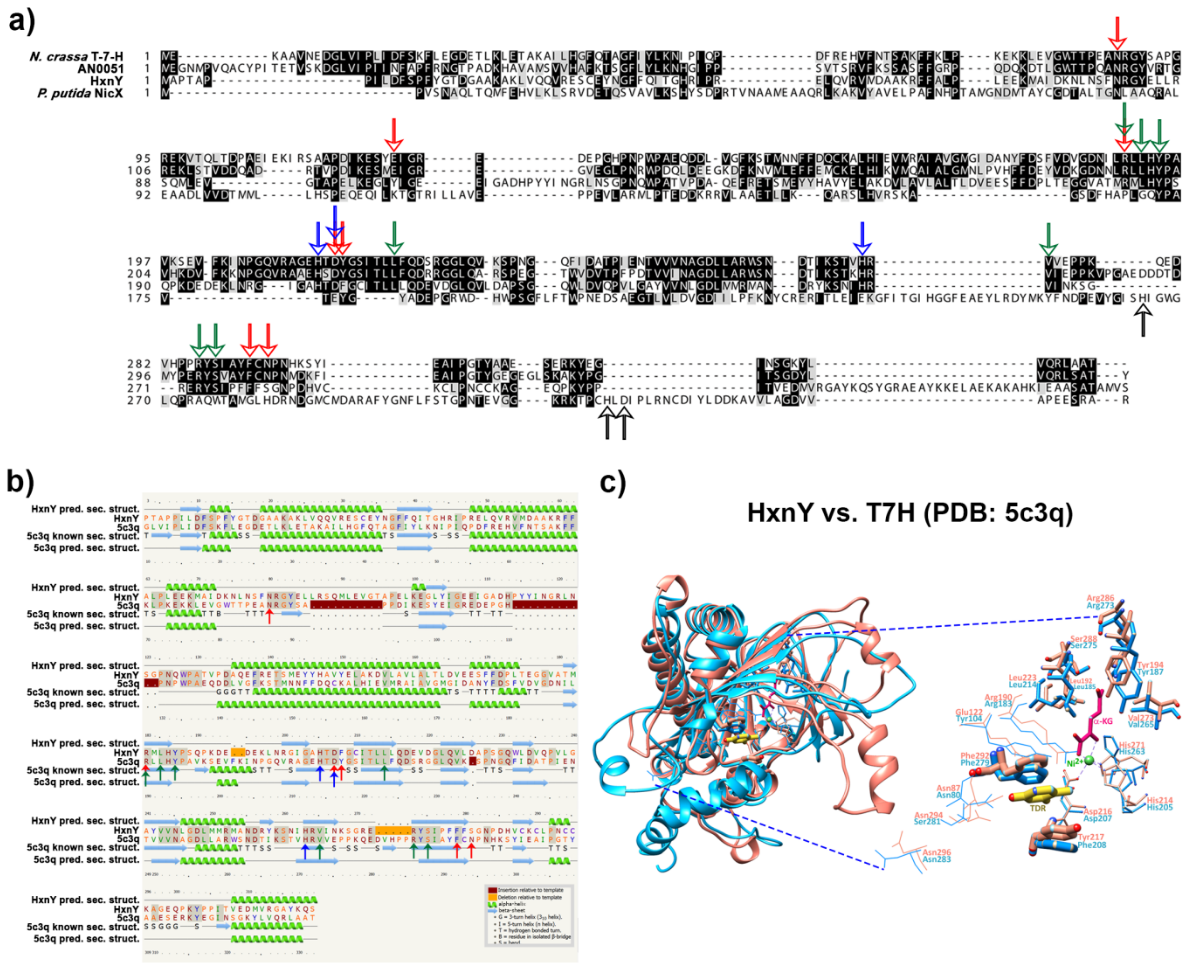

Supplementary Fig. 8. Comparative *in silico* analysis of HxnY.

HxnY is an α-ketoglutarate (α-KG) dependent dioxygenase that shares, 28.5% identity with the well-studied thymine-7-hydroxylase (T7H) of *Neurospora crassa* [16], 29.3% with its *A. nidulans* orthologue AN0051, but only 15% with the *A. nidulans* XanA and with the 2,5-dihydroxypyridine dioxygenase of *P. putida* KT2440 (NicX) [17], the latter catalysing the pyridine ring opening in the NA catabolism of this organism.

a) Sequence comparison of HxnY with Thymine-7-hydroxylase (T7H) from *N. crassa* (locus NCU06416) [16], its orthologue from *A. nidulans* (AN0051) and NicX, the 2,5-dihydroxypyridine hydroxylase (Q88FY1) from *P. putida* [17]. Alignment was carried out with Mafft G-INS-i, and visualised with Box shade. Red, blue and green arrows indicate the thymine binding, α-ketoglutarate binding and Fe(II) binding AA residues from T7H, respectively. Black arrows indicate the Fe(II) binding AA residues in NicX.

b) The predicted secondary structure of HxnY in comparison to both the known and the predicted secondary structures of Thymine-7-hydroxylase (T7H) from *N. crassa* (PDB: 5c3q). Image was obtained with Phyre2 [1]. Red, blue and green arrows as in Panel a.

c) Superposition of the structure of HxnY (for quality assessment of the model, see Supplementary Table 4) with T7H of *N. crassa* (PDB code: 5c3q) [16]. Salmon color shows T7H, blue color shows HxnY. TDR: thymine ligand of T7H (yellow thick sticks); $Ni2^{+}$: nickel ion (green sphere); α-KG: α-ketoglutarate ligand (magenta sticks). Thick sticks and medium-sized sticks are residues that establish π-π stacking interactions and direct or indirect hydrogen bonds with TDR ligand in T7H [16]. Residues with thin sticks coordinate the metal ion, while wires indicate residues that interact with the α-KG ligand.

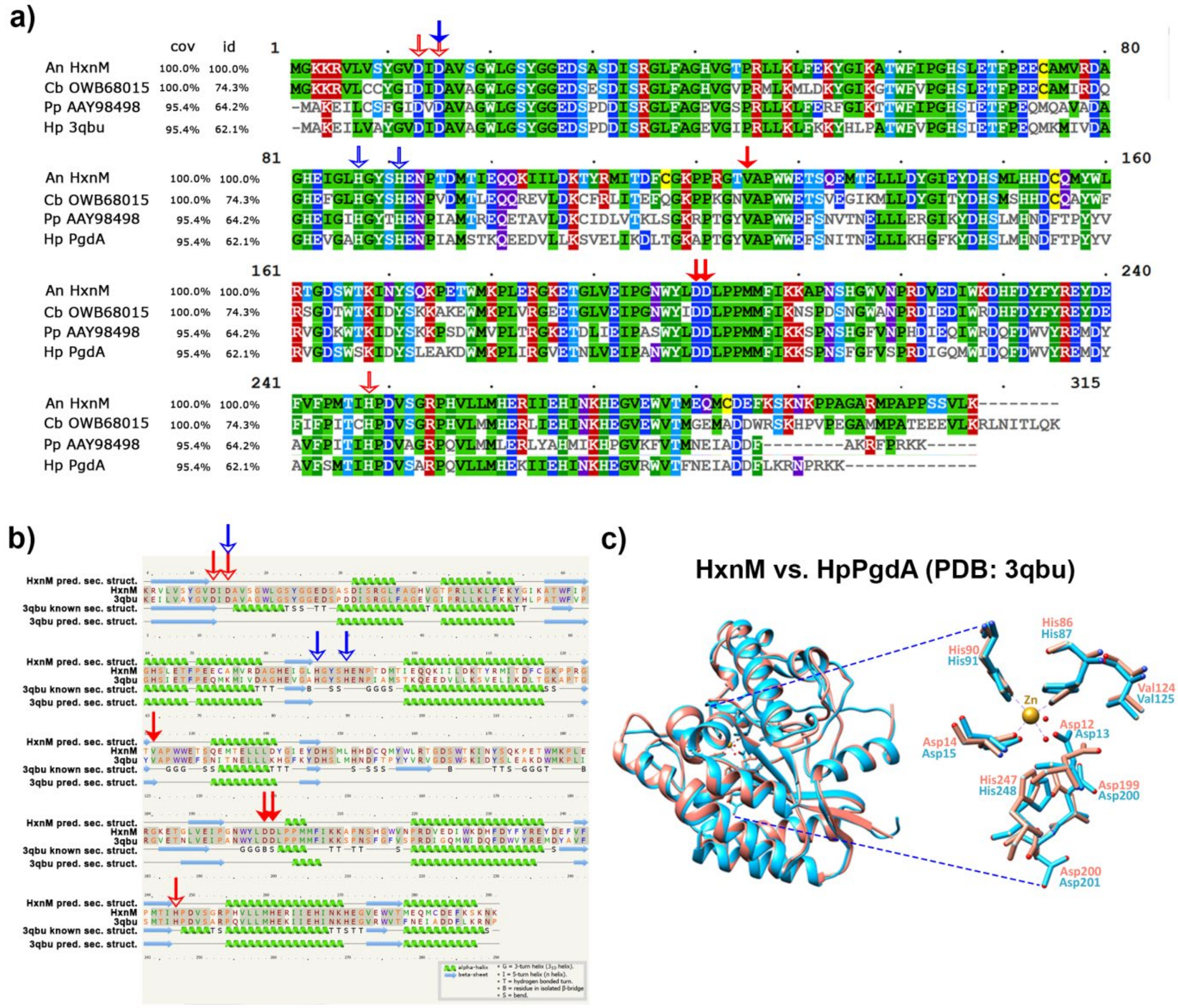

Supplementary Fig. 9. Comparison of HxnM to its homologs.

a) Primary sequence comparison of HxnM with the *Candida bondii* hydrolase (OWB68015), the cyclic imide hydrolase of *P. putida* (AAY98498) and HpPgdA of *H. pylori* [18]. Level of identity is indicated following protein names as % of identical amino acids and coverage of the aligned sequences. Alignment was carried out with Muscle, and visualised with MView. Hollow red arrows indicate the catalytic water binding AA residues, solid red arrows indicate other catalytic site residues and blue arrows mark the Zn ion coordinating residues in HpPgdA [18,19].

b) HxnM shares structural similarities with the HpPgdA of *H. pylori*. The predicted secondary structure of HxnM is shown in comparison to both the known and the predicted secondary structures of HpPgdA of *H. pylori* (PDB code: 3qbu). Image was obtained with Phyre2 [1]. Arrows mark residues as described for panel a.

c) Superposition of the structural model of HxnM (for quality assessment see Supplementary Table 4) with its closest known structural homologue, HpPgdA of *H. pylori* (PDI code: 3qbu).

Salmon color shows HpPgdA, blue color shows HxnM. The HpPgdA residues His86, His90 and Asp14 (His87, His91 and Asp15 in HxnM, respectively) accomodate the Zn ion (shown in yellow); His247, Asp12 and Asp14 (His248, Asp13 and Asp15 in HxnM, respectively) bind a catalytic water molecule and Asp199, Asp200 and Val124 (Asp200, Asp201 and Val125 in HxnM, respectively) serve other catalytic roles [18]. The striking conformity of the active site residues in HxnM indicates functional similarity to HpPgdA.

a)

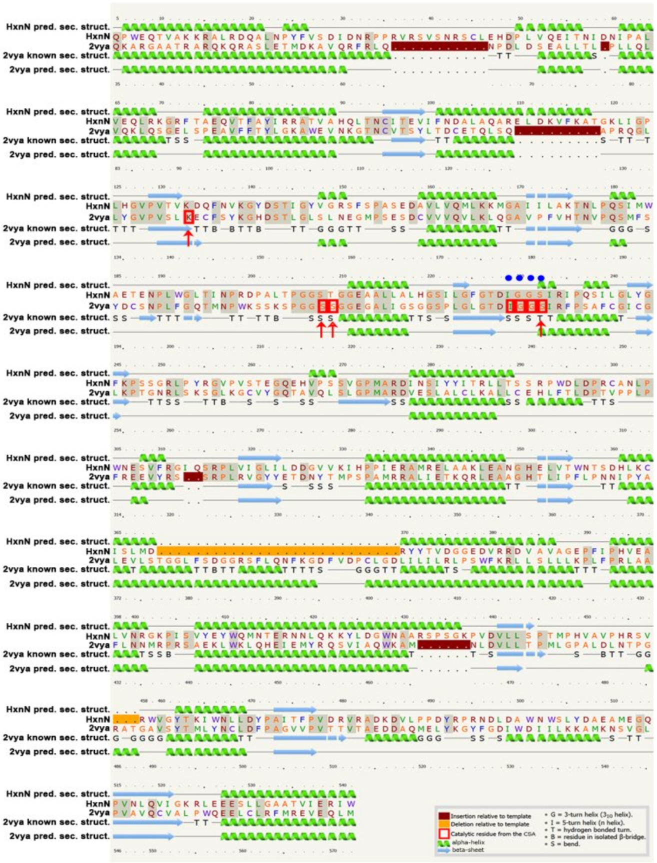

b)

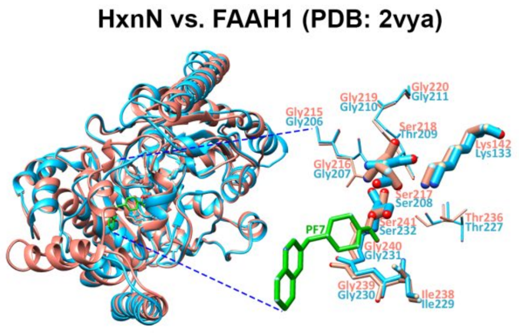

Supplementary Fig. 10. HxnN shares structural similarities with the Fatty-acid amide hydrolase 1, FAAH, from *Rattus norvegicus*.

a) The predicted secondary structure of HxnN is shown in comparison to both the known and the predicted secondary structures of FAAH of *R. norvegicus* (PDB code: 2vya). Image was obtained with Phyre2 [1]. Solid red arrows indicate the catalytic triad, the hollow red arrow indicates a Ser that interacts with the catalytic triad and the blue dots indicate the oxyanion hole forming AA residues in FAAH [20,21].

b) Superposition of the structural model of HxnN (for quality assessment see Supplementary Table 4) with its known structural homologue, fatty acid amide hydrolase 1 (FAAH1) of *R. norvegicus* (PDA code: 2vya). Salmon color shows FAAH1, blue color shows HxnN. The Ser217, Ser241 and Lys142 residues in FAAH1 (Ser208, Ser232 and Lys133 in HxnN, respectively) form the catalytic triad for amide bond hydrolysis (thick sticks) [21]. The Ile238, Gly239, Gly240 and Ser241 residues in FAAH1 (Ile229, Gly230, Gly231 and Ser232 in HxnN, respectively) form the oxyanion hole (medium-sized sticks). The catalytic triad in FAAH1 is supported by Gly215, Gly216, Ser218, Gly219, Gly220 and Thr236 residues (thin

sticks) (Gly206, Gly207, Thr209, Gly210, Gly211 and Thr227 in HxnN) [20]. The 4-(quinolin-3-ylmethyl)piperidine-1-carboxyl acid (PF7) ligand with FAAH1 is shown in green sticks. HxnN includes a GatA type amidase domain (Asp-tRNAAsn/Glu-tRNAGln amidotransferase A subunit or related amidase domain) (pfam01425) with a transmembrane segment (463-478 AAs) (identified by Phyre2 analysis [1]). Its putative orthologues from *S. cerevisiae* (Amd2p) and *Schizosaccharomyces pombe* (Fah1p) are putative amidases.

SUPPELEMENTARY TABLES
for

A complete nicotinate degradation pathway in the microbial eukaryote *Aspergillus nidulans*

Eszter Bokor[1], Judit Ámon[1], Mónika Varga[1], András Szekeres[1], Zsófia Hegedűs[1], Tamás Jakusch[2], Zsolt Szakonyi[3], Michel Flipphi[4], Csaba Vágvölgyi[1], Attila Gácser[5,6], Claudio Scazzocchio[7,8]* and Zsuzsanna Hamari[1]*

[1]University of Szeged Faculty of Science and Informatics, Department of Microbiology, Szeged, Hungary

[2]University of Szeged Faculty of Science and Informatics, Department of Inorganic and Analytical Chemistry, Szeged, Hungary

[3]University of Szeged Faculty of Pharmacy, Institute of Pharmaceutical Chemistry, Szeged, Hungary

[4] Institute de Génétique et Microbiologie, Université Paris-Sud, Orsay, France

[5] HCEMM-USZ Fungal Pathogens Research Group, University of Szeged Faculty of Science and Informatics, Department of Microbiology, Szeged, Hungary

[6] MTA-SZTE "Lendület" Mycobiome Research Group, University of Szeged, Szeged, Hungary

[7] Section of Microbiology, Department of Infectious Diseases, Imperial College, London, United Kingdom

[8] Université Paris-Saclay, CEA, CNRS, Institute for Integrative Biology of the Cell (I2BC), 91198, Gif-sur-Yvette, France

Present address of M.F.: Department of Biochemical Engineering, Faculty of Science and Technology, University of Debrecen, Debrecen, Hungary

* Corresponding authors:

hamari@bio.u-szeged.hu,

c.scazzocchio@imperial.ac.uk

Content:

Supplementary Table 1: **UHPLC-HRMS characteristics of the intermediates**

Supplementary Table 2: **NMR results**

Supplementary Table 3: **List of *A. nidulans* strains used in this work.**

Supplementary Table 4: **Results of protein modelling and superpositioning of these models with their closest known structural homologs**

Supplementary Table 5: **List of primers used in this study.**

Supplementary Table 1. UHPLC-HRMS characteristics of the intermediates

Compound	Elemental composition	RT (min)	Precursor ion: Form	Precursor ion: Accurate mass	MS/MS fragments
**6-NA***	$C_6H_5NO_3$	3.88	$[M+H]^+$	140.0339	122.0239 (54), 112.0401 (100), 96.0452 (49), 95.0133 (15), 94.0294 (31), 78.0342 (76), 66.0340 (6)

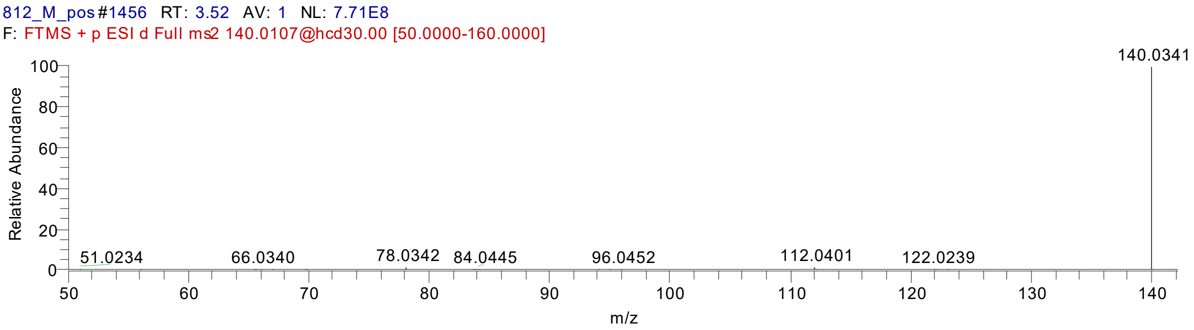

Compound	Elemental composition	RT (min)	Precursor ion: Form	Precursor ion: Accurate mass	MS/MS fragments
**2,5-DP***	$C_5H_5NO_2$	4.92	$[M+H]^+$	112.0400	94.0295 (100), 84.0450 (35), 76.0186 (14), 66.0341 (6), 56.0499 (4), 53.0026 (10)

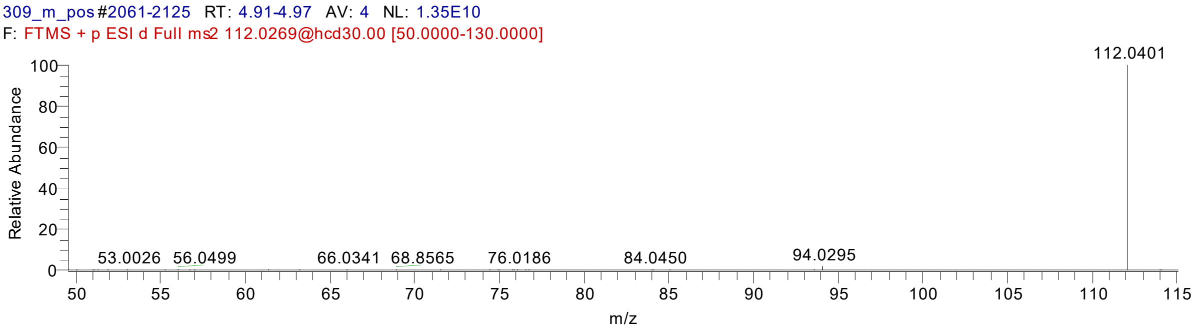

Compound	Elemental composition	RT (min)	Precursor ion: Form	Precursor ion: Accurate mass	MS/MS fragments
**5,6-DHPip-2-O***	$C_5H_9NO_3$	4.46	$[M+H]^+$	132.0656	115.0397 (100), 114.0556 (38), 97.0293 (25), 87.0447 (11), 86.0607 (9), 71.0494 (9), 69.0337 (17), 59.0495 (4), 55.0183 (13)

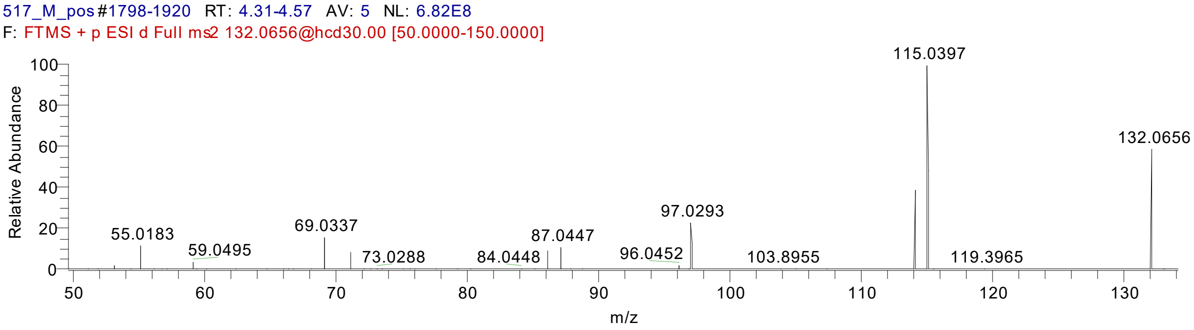

**3-HPip-2,6-DO***	$C_5H_7NO_3$	1.63	$[M+H]^+$	130.0507	102.0559 (15), 85.0290 (100), 84.0450 (10), 57.0339 (5)

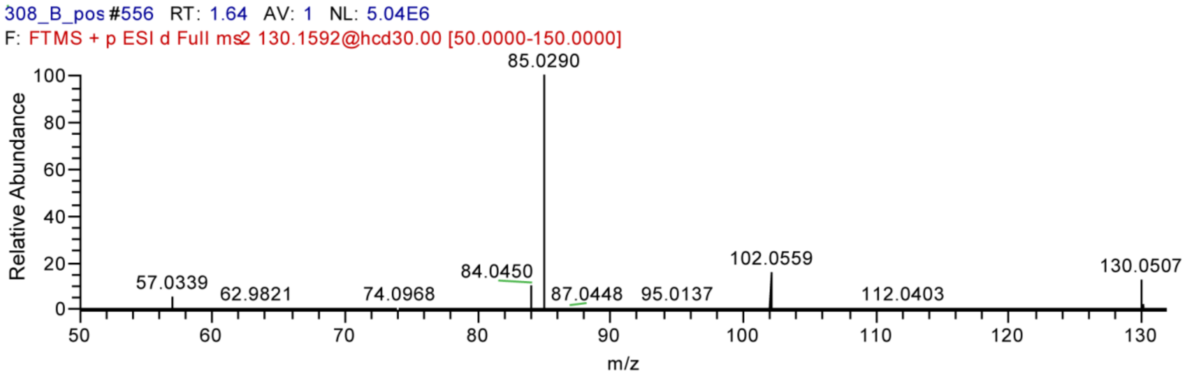

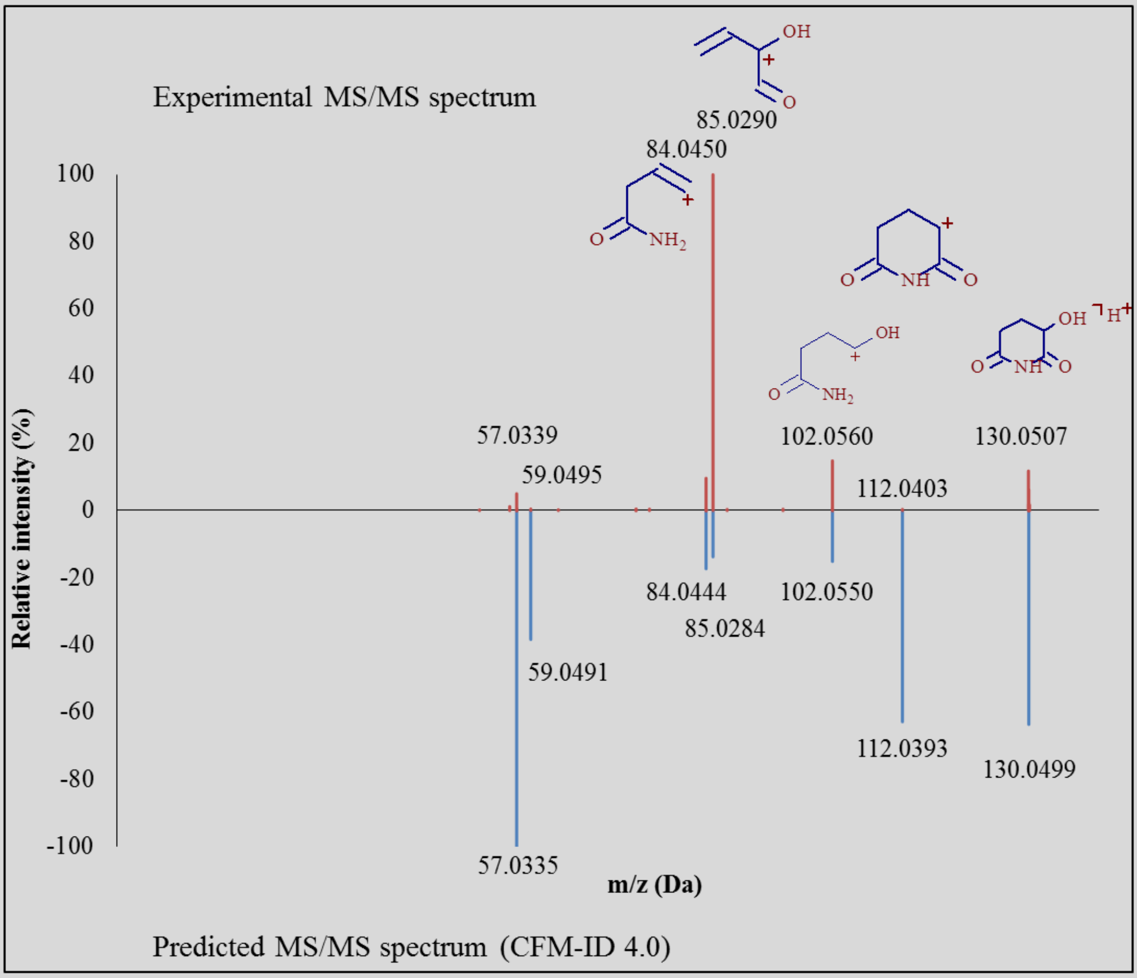

Comparative presentation of experimental and *in silico* predicted MS/MS fragments of 3-HPip-2,6-DO.
The head-to-tail plot compare the experimental MS/MS spectra of 3-HPip-2,6-DO collected at 30 HCD with the corresponding *in silico* spectra predicted by using CFM-ID 4.0. Structures in blue color were predicted by using HighChem Mass Frontier 7.0

**α-HGA***	$C_5H_9NO_4$	4.83	$[M+H]^-$	146.0461	129.0195 (18), 128.0354 (47), 101.0245 (8), 100.0404 (86), 85.0295 (16), 82.0297 (100), 72.0452 (7)

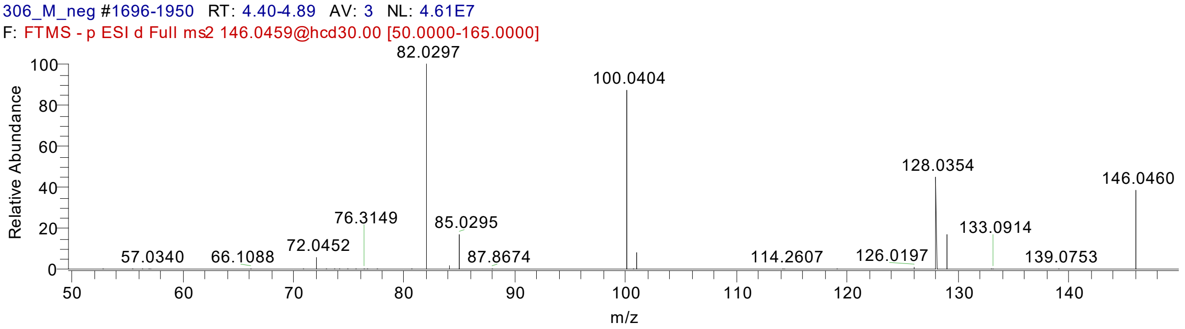

**α-HG***	$C_5H_8O_5$	3.88	$[M+H]^-$	147.0298	129.0194 (100), 103.0400 (26), 101.0244 (25), 85.0295 (35), 57.0341 (14)

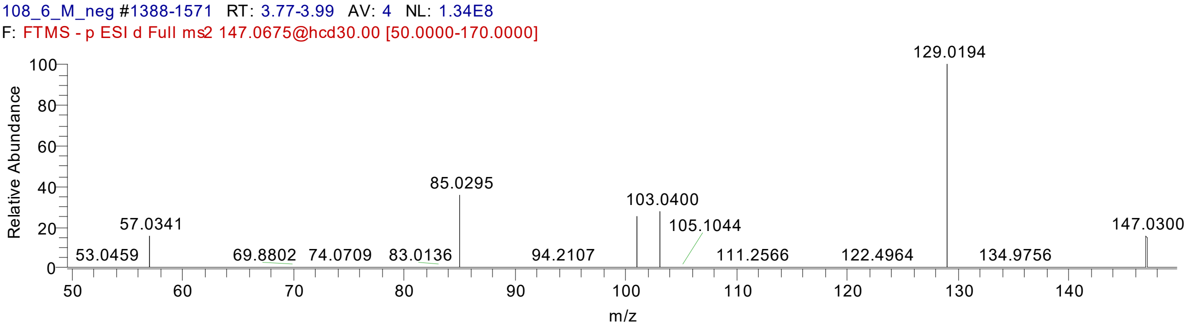

*6-NA: 6-hydroxynicotinic acid; 2,5-DP: 2,5-dihydroxypyridine, 5,6-DHPip-2-O: (5*S*,6*R*)-(+)-dihydroxypiperidine-2-one, 3-HPip-2,6-DO: 3-hydroxypiperidine-2,6-dione, α-HGA: (*S*)-(+)-α-hydroxyglutaramate, α-HG: α-hydroxyglutarate.

Supplementary Table 2. NMR results

a)

^{1}H, ^{13}C (jmod) and 2D NMR spectral assignment for (5*S*,6*R*)-(+)-dihydroxypiperidine-2-one (500 MHz, CD_3OD)
(In ^{1}H-^{1}H TOCSY spectra all protons have cross-peaks with each other.)

δC(jmod)			δH	HMBC
1	174.7(+)		-	
2	27.6(+)	a	2.26(ddd)	C1, C3, C4
		b	2.50 (ddd)	C1, C3, C4
3	23.7(+)	a	2.14(dddd)	C1, C2, C4, C5
		b	1.80(dddd)	C1, C2, C4, C5
4	68.1(–)		3.79(ddd)	C2, C3, C5
5	80.2(–)		4.74 (d)	C1, C3, C4

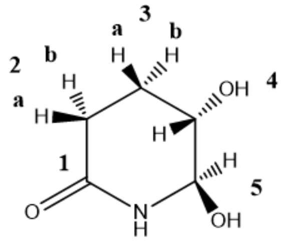

b)

$J_{H\text{-}H}$ couplings (^2J and ^3J) for (5*S*,6*R*)-(+)-dihydroxypiperidine-2-one (500 MHz, CD_3OH)

	2a	2b	3a	3b	4	5
2a	-	*18.0*[a]	6.5	3.5		
2b	*18.0*[a]	-	10.7	6.7		
3a	6.5	10.7	-	*13.7*[a]	2.6	
3b	3.5	6.7	*13.7*[a]	-	5.3	
4			2.6	5.3	-	3.0
5					3.0	-

[a] marks ^2J

c)

^{1}H, ^{13}C (jmod) and 2D NMR spectral assignment for (*S*)-(+)-α-hydroxyglutaramate (500 MHz, DMSO)
(Based on ^{1}H-^{1}H NOESY the amide protons (0a/0b) are closer to 2a/2b than 3a/3b and 4.)

	δC(jmod)		δH	^{1}H-^{1}H COSY	^{1}H-^{1}H TOCSY	HMBC
0		a	6.65(s)	0b	0b	
		b	7.22(s)	0a	0a	
1	174.9(+)		-			
2	31,7(+)	a	2.12(m)	2b, 3a, 3b	2b, 3a, 3b, 4	C1, C3, C4
		b	2.18(m)	2a, 3a, 3b	2a, 3a, 3b, 4	C1, C3, C4
3	30.8(+)	a	1.88(m)	2a, 2b, 3b, 4	2a, 2b, 3b, 4	C1, C2, C4, C5
		b	1.68(m)	2a, 2b, 3a, 4	2a, 2b, 3a, 4	C1, C2, C4, C5
4	70.3(–)		3.94(dd)(^3J = 4.6 / 7.6)	3a, 3b	2a, 2b, 3a, 3b	C2, C3, C5
5	176.4(+)		-			

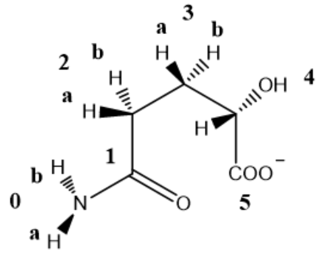

Optical rotation of (*S*)-(+)-α-HGA ($[\alpha]_D^{20}$ +7.8°, c = 0.077, MeOH) and (5*S*,6*R*)-(+)-DHPip-2-O ($[\alpha]_D^{20}$ +51.6°, c= 0.133, MeOH) were measured with a Jasco P 2000 Polarimeter.

d) **Raw NMR data are the following**

^{1}H NMR of (5*S*,6*R*)-dihydroxypiperidine-2-one (500 MHz, DMSO-d6)

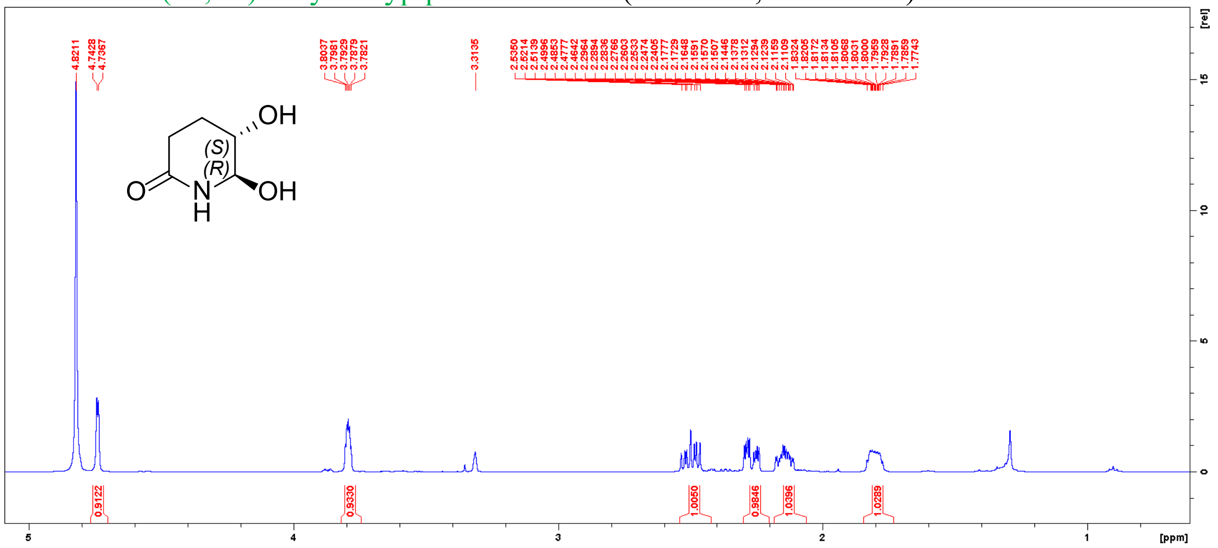

^{13}C NMR of (5*S*,6*R*)-dihydroxypiperidine-2-one (125 MHz, DMSO-d6)

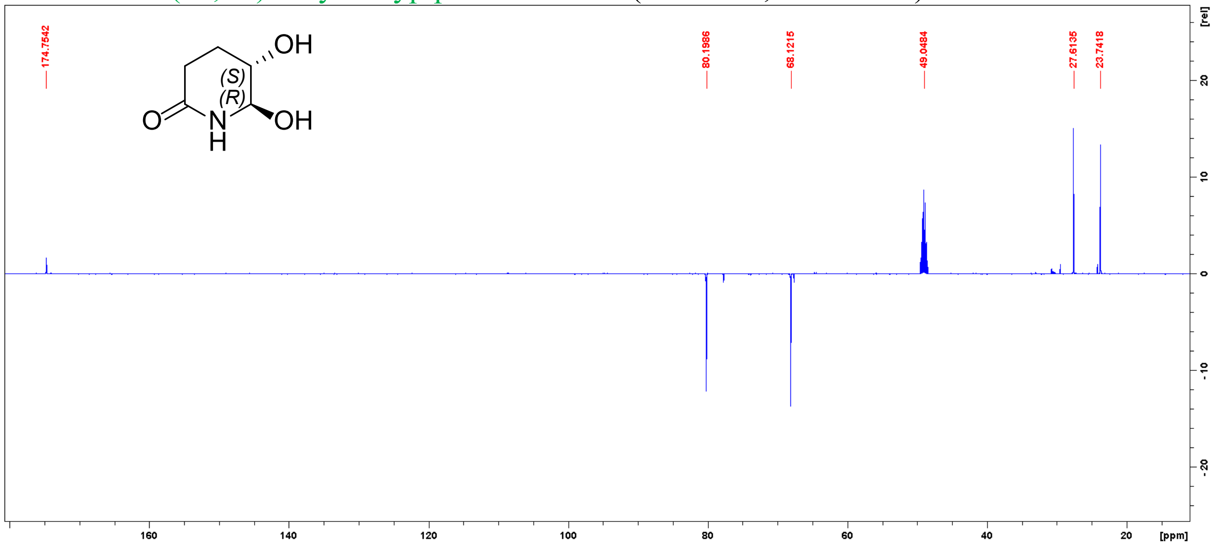

COSY spectrum of (5*S*,6*R*)-dihydroxypiperidine-2-one

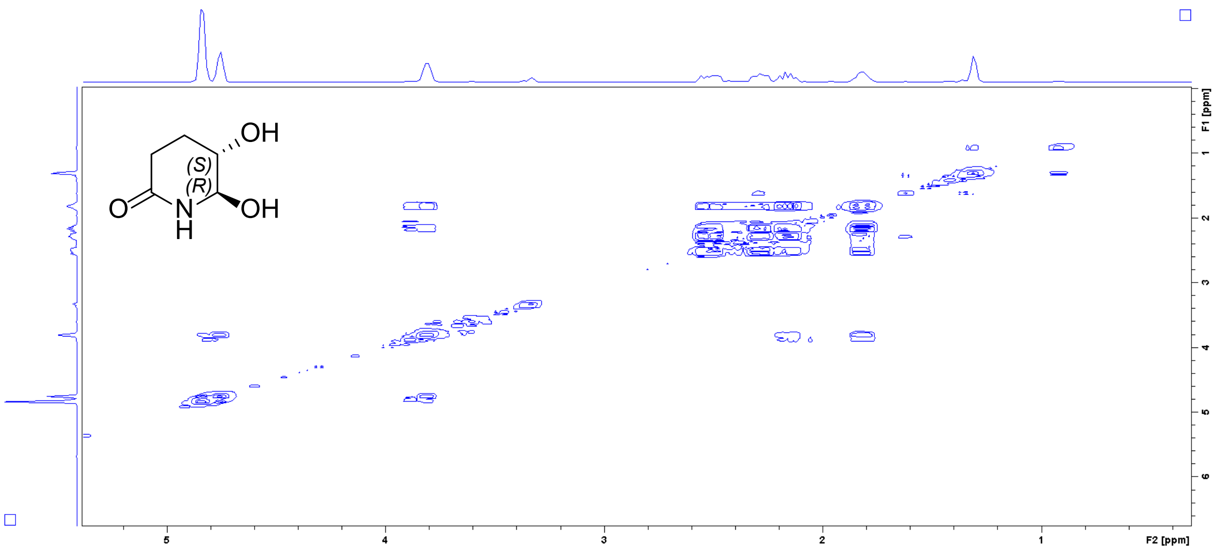

NOESY spectrum of (5*S*,6*R*)-dihydroxypiperidine-2-one

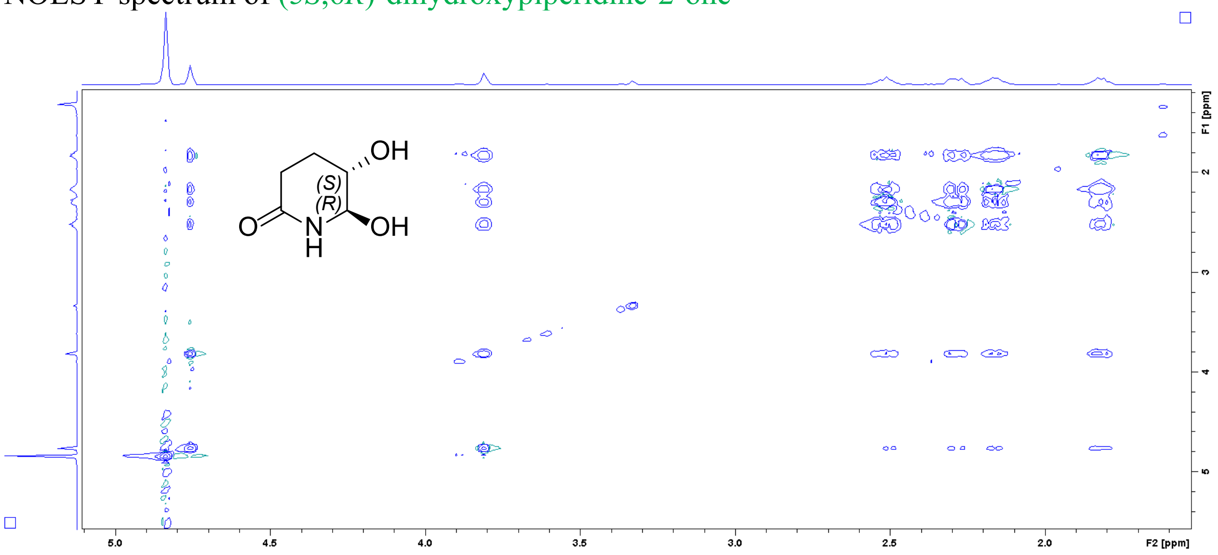

HMBC spectrum of (5*S*,6*R*)-dihydroxypiperidine-2-one

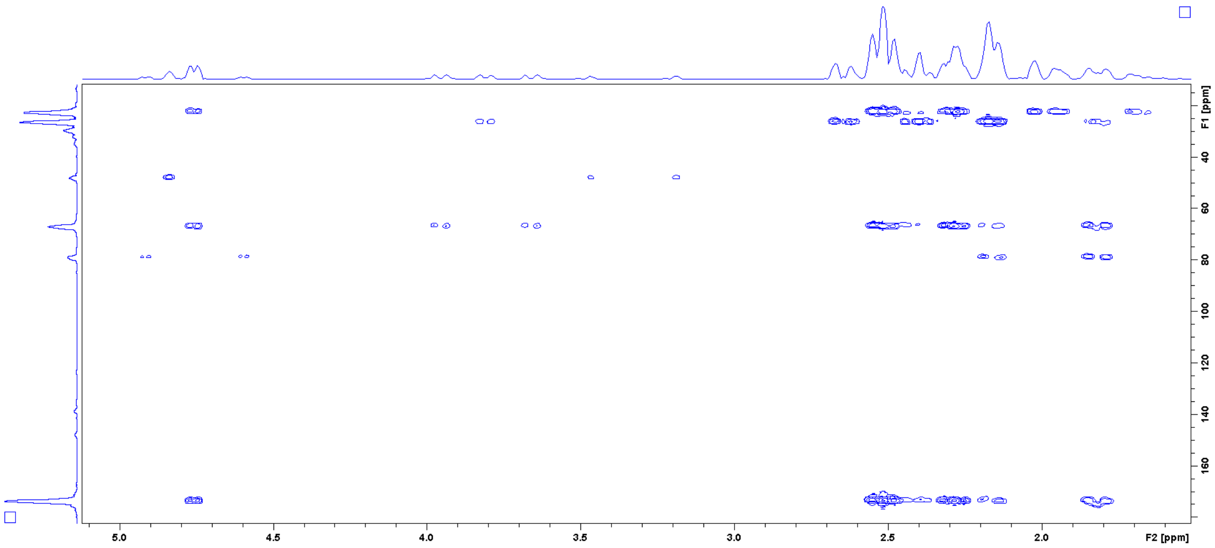
5.0
4.5
4.0
3.5
3.0
2.5
2.0
F2 [ppm]
F1 [ppm]
40
60
80
100
120
140
160

HSQC spectrum of (5*S*,6*R*)-dihydroxypiperidine-2-one

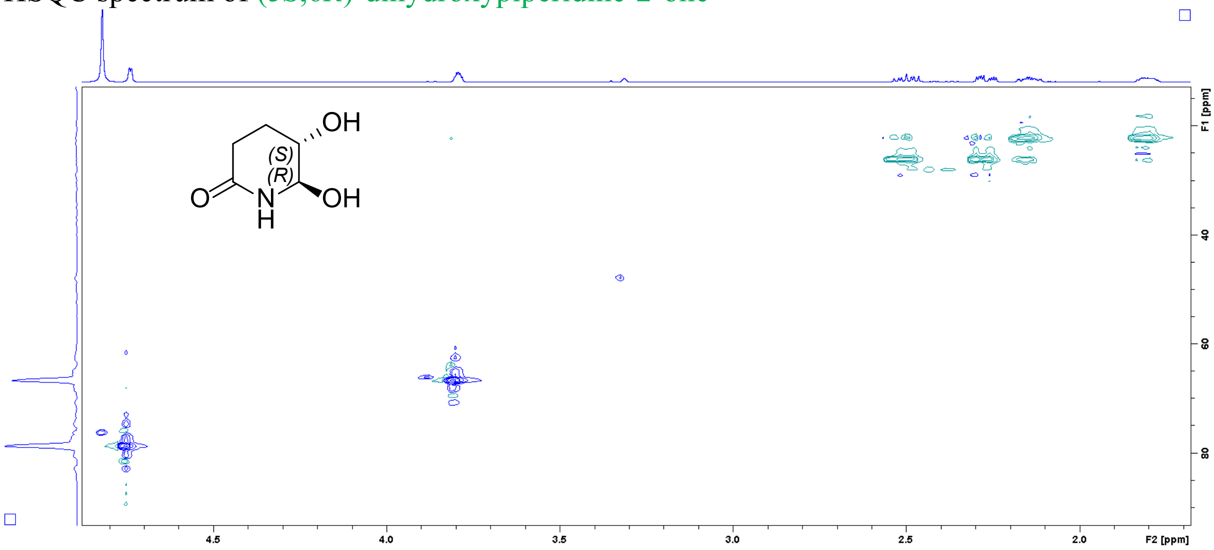

^{1}H NMR of (*S*)-α-hydroxyglutaramate (500 MHz, DMSO-d6)

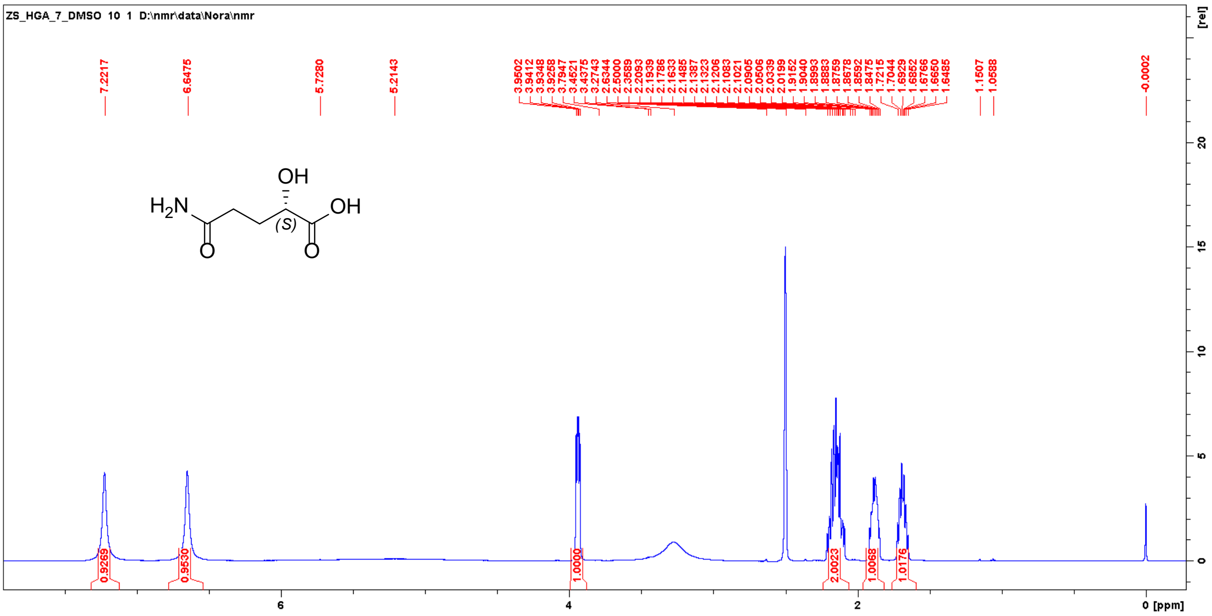

^{13}C NMR of (*S*)-α-hydroxyglutaramate (125 MHz, DMSO-d6)

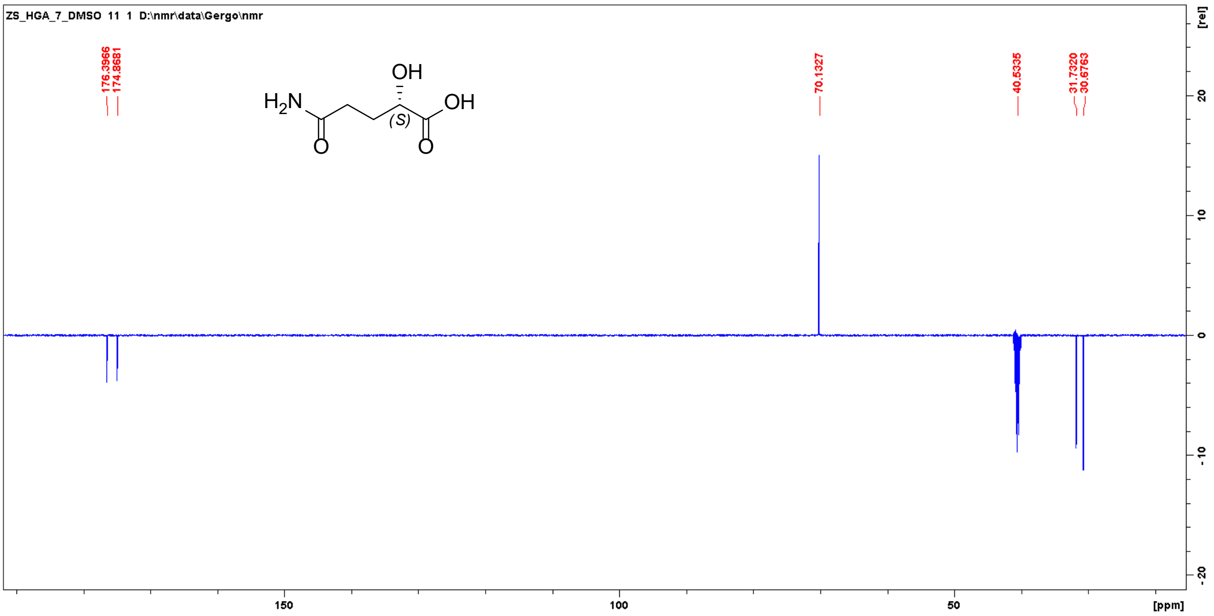

COSY spectrum of (*S*)-α-hydroxyglutaramate

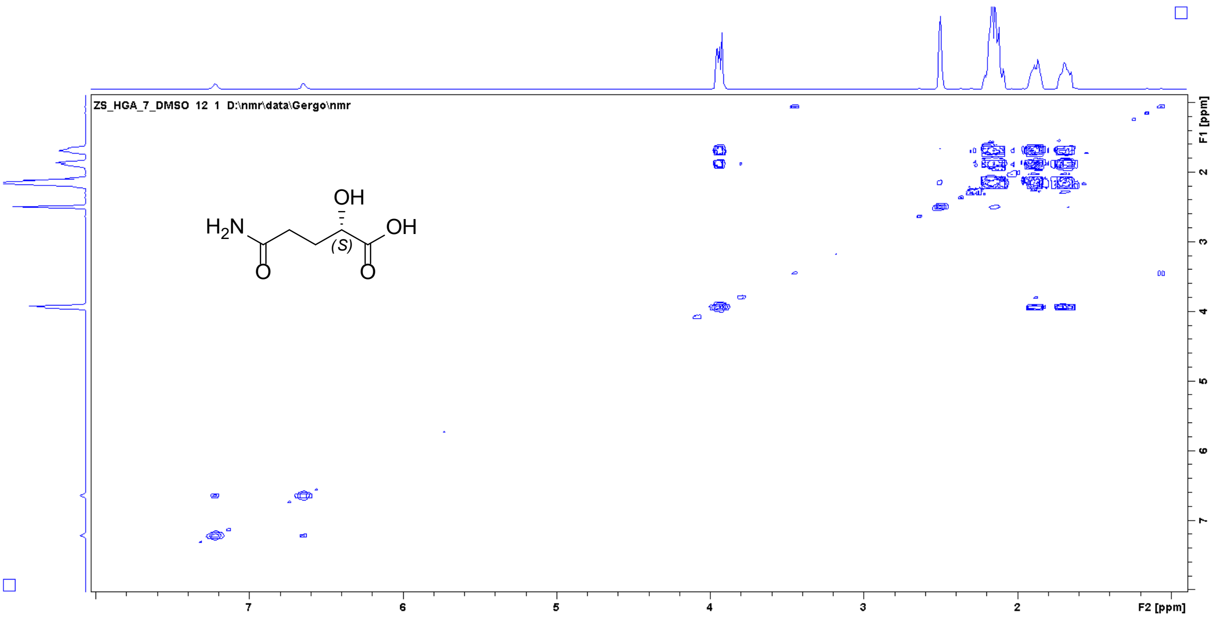

NOESY spectrum of (*S*)-α-hydroxyglutaramate

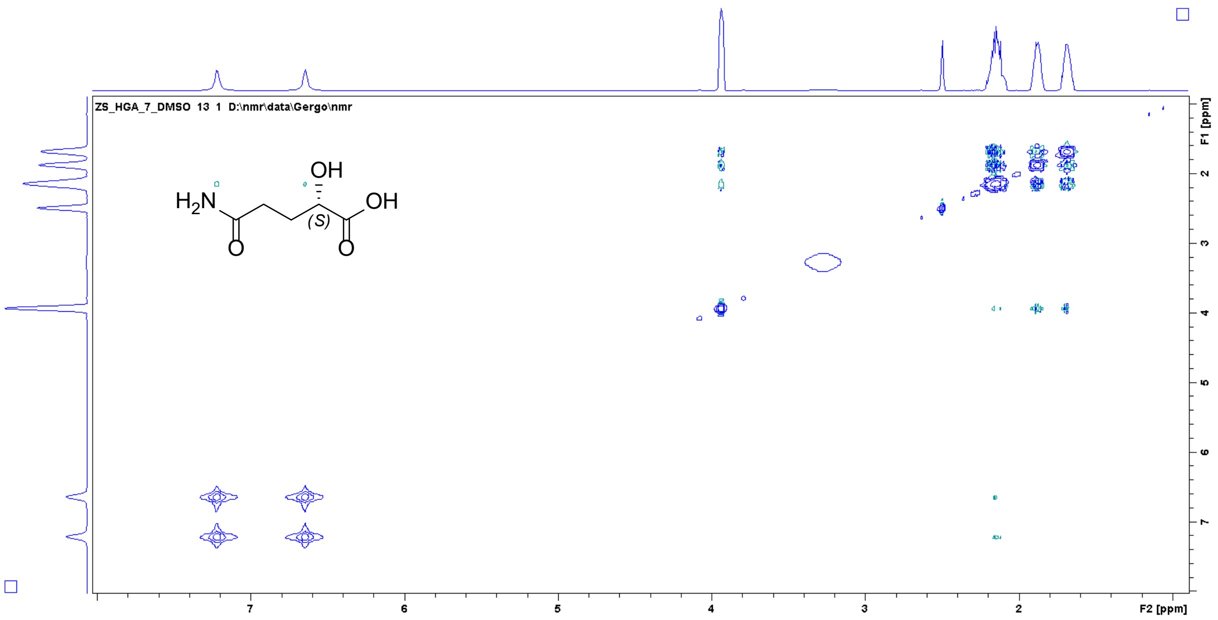

HSQC spectrum of (*S*)-α-hydroxyglutaramate

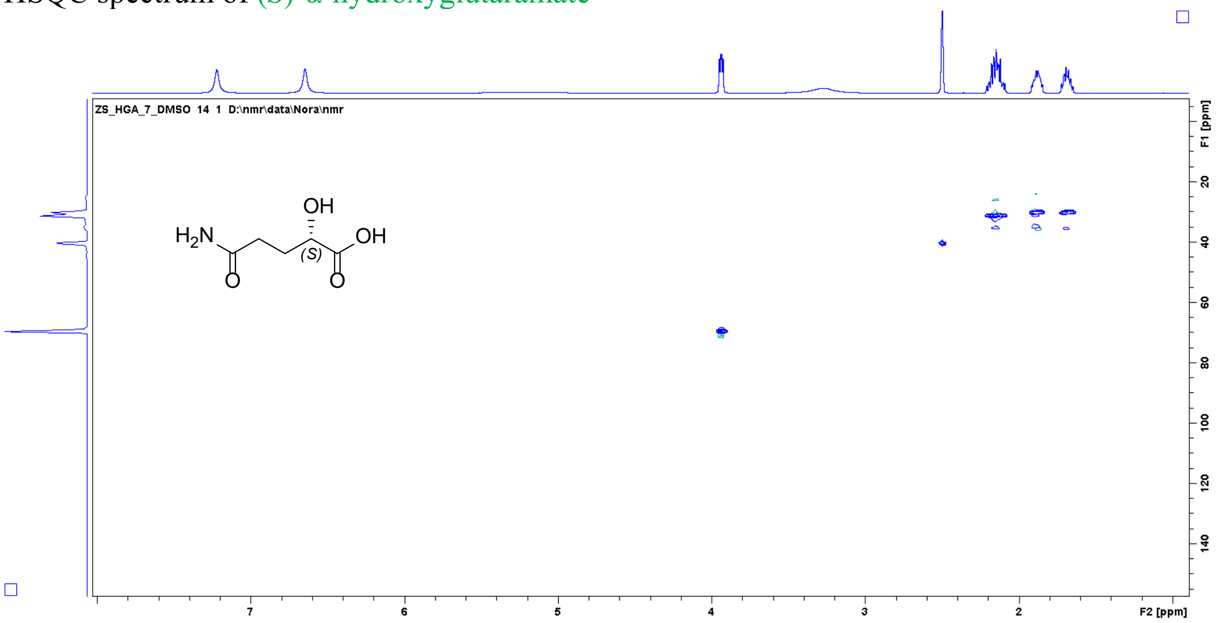

HMBC spectrum of (*S*)-α-hydroxyglutaramate

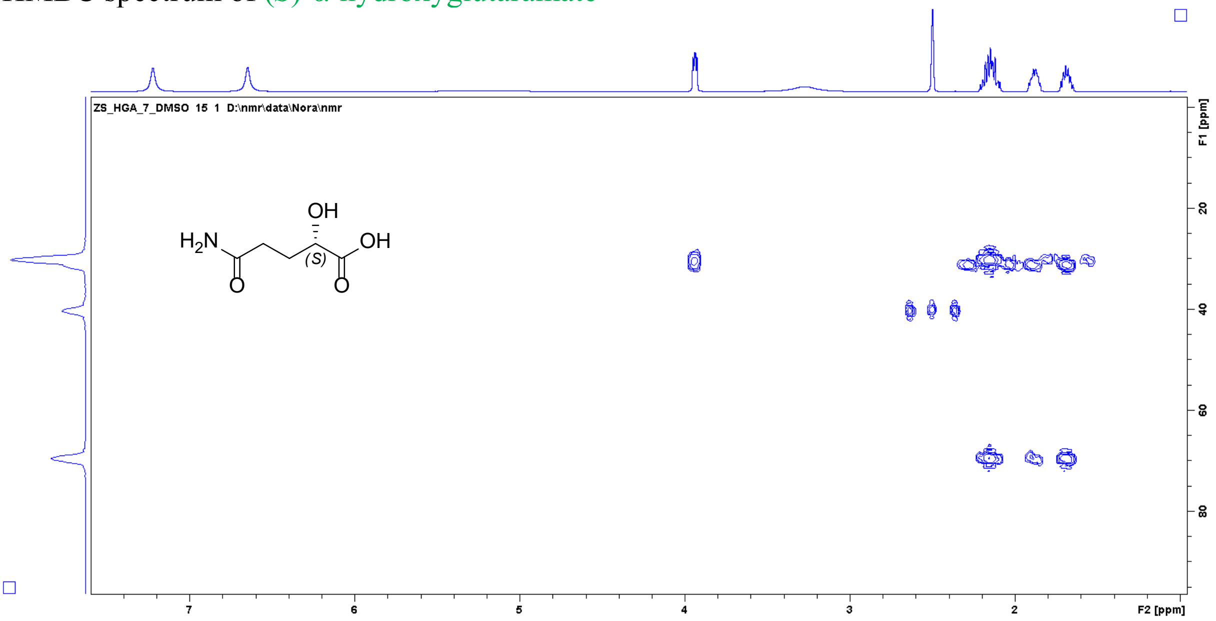

Supplementary Table 3. List of *A. nidulans* strains used in this work. All strains listed are *veA1* mutants.

Strain	Genotype	Purpose	Reference
A148	*pabaA1 wA3*	parental strain in genetic crosses with TN02 A21	provided by Herb Arst
CS2638	*yA2 pantoB100 riboB2 nicB8 fpaD43 acet^{-}*	parental strain in genetic crosses with HZS.614, HZS.221 and HZS.226	provided by S. Amillis
FGSCA26	*biA1*	mRNA expression analysis	[22]
FGSCA872/ CS51	*hxnR^{c}7 biA1*	growth test; enzyme assay; parental strain in genetic crosses with HZS.293, HZS.294 and HZS.288; metabolite analysis	[8]
TN02 A21	*riboB2 pyroA4 nkuAΔ::argB^{+}*	recipient strain in transformation experiment to obtain *hxnP, hxnV, hxnN, hxnR* and *hxnZ* deletions; growth test	[23]
NA1322	*acuL::GFP(at Locus acuL) pabaA1 biA1 argB2; in trans pDsRed-SKL-argB^{+}* plasmid in 1 copy	parental strain in genetic crosses with HZS.305	[24]
HZS.98	*pantoB100 pabaA1*	parental strain in genetic crosses with HZS.222	[3]
HZS.106	*hxnSΔ::zeo hxAΔ::zeo pyrG89 pantoB100 biA1 pyr4 in trans*	parental strain in genetic crosses with HZS.122	[3]
HZS.120	*riboB2 pabaA1*	growth test; recipient strain in transformation experiment to obtain *hxnT* and *hxnY* deletions	[25]
HZS.122	*riboB2 pabaA1 yA2*	parental strain in genetic crosses with HZS.106	[3]

HZS.123	*anA1 riboB2 pabaA1*	parental strain in genetic crosses with HZS.307 and HZS.393	this work
HZS.143	*pabaA1 yA2*	parental strain in genetic crosses with HZS.309	this work
HZS.145	*veA1*	enzyme assay	[3]
HZS.221	*hxnPΔ::riboB⁺ riboB2 pyroA4 nkuAΔ::argB⁺*	growth test; parental strain in genetic crosses with HZS.399 and CS2638; recipient strain in transformation experiment to obtain *hxnP* and *hxnZ* double deletion mutants	this work (obtained by transformation of the "uphxnP-riboB⁺-downhxnP" substitution cassette into TN02 A21)
HZS.222	*hxnTΔ::pabaA⁺ pabaA1 riboB2*	growth test; parental strain in genetic crosses with HZS.397, HZS.223, HZS.726	this work (obtained by transformation of the "uphxnT-pabaA⁺-downhxnT" substitution cassette into HZS.120)
HZS.223	*hxnYΔ::riboB⁺ riboB2 pabaA1*	growth test; parental strain in genetic crosses with HZS.395, HZS.222, HZS.548 and HZS.568	this work (obtained by transformation of the "uphxnY-riboB⁺-downhxnY" substitution cassette into HZS.120)
HZS.226	*hxnZΔ::riboB⁺ riboB2 pyroA4 nkuAΔ::argB⁺*	growth test; parental strain in genetic crosses with HZS.399 and CS2638	this work (obtained by transformation of the "uphxnZ-riboB⁺-downhxnZ" substitution cassette into TN02 A21)
HZS.227	*riboB2, pantoB100, yA2*	parental strain in genetic crosses with HZS.308	this work
HZS.245	*hxAΔ::zeo riboB2 pantoB100 biA1*	enzyme assay	[3]
HZS.251	*riboB2 biA1 pabaA1*	recipient strain in transformation experiment to obtain *hxnM* deletion	this work (obtained by genetic cross of HZS.106 with HZS.122)

HZS.254	*hxnSΔ::zeo biA1 pyr4 in trans*	enzyme assay	[3]
HZS.267	*riboB2 pantoB100*	recipient strain for transformation experiment to obtain *hxnM* and *hxnW* deletions	this work (obtained by genetic cross of HZS.308 with HZS.227)
HZS.288	*hxnNΔ::riboB*$^{+}$ *pyroA4 nkuΔ::argB*$^{+}$ *riboB2*	growth test; parental strain in genetic crosses with FGSC A872	this work (by transformation of the "uphxnN-riboB^{+}-downhxnN" substitution cassette into TN02 A21)
HZS.292	*hxnMΔ::riboB*$^{+}$ *biA1 pabaA1 riboB2*	parental strain in genetic crosses with HZS.294 and HZS.393	this work (by transformation of the "uphxnM-riboB^{+}-downhxnM" substitution cassette into HZS.251)
HZS.293	*hxnMΔ::riboB*$^{+}$ *pantoB100 riboB2*	growth test; parental strain in genetic crosses with FGSC A872 and HZS.297	this work (by transformation of the "uphxnM-riboB^{+}-downhxnM" substitution cassette into HZS.267)
HZS. 294	*hxnVΔ::riboB*$^{+}$ *pyroA4 nkuΔ::argB*$^{+}$ *riboB2*	parental strain in genetic crosses with HZS.292 and FGSC A872, growth test	this work (by transformation of the "uphxnV-riboB^{+}-downhxnV" substitution cassette into TN02 A21)
HZS.296	*hxnXΔ::riboB*$^{+}$ *biA1 pabaA1 riboB2*	parental strain is genetic crosses with HZS.316	this work (by transformation of the "uphxnX-riboB^{+}-downhxnX" substitution cassette into HZS.251)
HZS.297	*hxnXΔ::riboB*$^{+}$ *biA1 pabaA1 riboB2*	parental strain in genetic crosses with HZS.293	this work (by transformation of the "uphxnX-riboB^{+}-downhxnX" substitution cassette into HZS.251)
HZS.305	*hxnXΔ::riboB pantoB100 biA1 (riboB2)*	parental strain in genetic crosses with NA1322	this work (obtained by genetic cross of HZS.296 with HZS.227)
HZS.306	*hxnNΔ::riboB*$^{+}$ *pyroA4 hxnR*^{c}*7* (*nkuΔ::argB*$^{+}$ *riboB2*)	growth test; metabolite analysis	this work (obtained by genetic cross of HZS.288 with FGSC A872)
HZS.307	*hxnR*^{c}*7 pantoB100 biA1*	parental strain in genetic crosses with HZS.123 and HZS.393	this work (obtained by genetic cross of HZS.293 with FGSC A872)

HZS.308	*hxnMΔ::riboB*$^{+}$ *pantoB100 hxnR*^{c}*7* (*riboB2*)	growth test; metabolite analysis	this work (obtained by genetic cross of HZS.293 with FGSC A872)
HZS.309	*hxnVΔ::riboB*$^{+}$ *riboB2 pyroA4 hxnR*^{c}*7* (*nkuΔ::argB*$^{+}$)	growth test; metabolite analysis; parental strain in genetic crosses with HZS.143 and HZS.429	this work (obtained by genetic cross of HZS.294 with FGSC A872)
HZS.310	*hxnXΔ::riboB*$^{+}$ *hxnR*^{c}*7* (*riboB2 nkuΔ::argB*$^{+}$)	growth test	this work (obtained by genetic cross of HZS.296 and HZS.316)
HZS.316	*hxnR*^{c}*7 pyroA4* (*nkuΔ::argB*$^{+}$ *riboB2*)	parental strain is genetic crosses with HZS.296	this work (obtained by genetic cross of HZS.288 and FGSC A872)
HZS.393	*hxnWΔ::riboB*$^{+}$ *riboB2 pantoB100*	growth test; parental strain in genetic crosses with HZS.292 and HZS.123	this work (by transformation of the "uphxnW-riboB^{+}-downhxnW" substitution cassette into HZS.267
HZS.395	*hxnR*^{c}*7 riboB2 biA1*	parental strain in genetic crosses with HZS.223	this work (obtained by genetic cross of HZS.307 with HZS.123)
HZS. 397	*hxnR*^{c}*7 pabaA1 anA1*	parental strain in genetic crosses with HZS.222 and HZS.599	this work (obtained by genetic cross of HZS.307 with HZS.123)
HZS.398	*hxnR*^{c}*7 riboB2 pabaA1 biA1*	parental strain in genetic crosses with HZS.795	this work (obtained by genetic cross of HZS.307 with HZS.123)
HZS.399	*hxnR*^{c}*7 riboB2 pabaA1*	parental strain in genetic crosses with HZS.221 and HZS.226	this work (obtained by genetic cross of HZS.307 with HZS.123)
HZS.404	*hxnR*^{c}*7 riboB2 pantoB100*	recipient strain for transformation experiment to obtain *hxnV hxnW* double deletion, *hxnX hxnW* double deletion and *hxnV hxnW hxnX* triple deletion mutants; parental strain in genetic crosses with HZS.726 and HZS.727	this work (obtained by genetic cross of HZS.307 with HZS.123)

HZS.427	*hxnTΔ::pabaA*$^{+}$ *hxnR*^{c}*7 anA1 pabaA1*	enzyme assay; metabolite analysis; parental strain in genetic crosses with HZS.517, HZS.537, HZS.726, HZS.749, HZS.751 and HZS.783	this work (obtained by genetic cross of HZS.222 with HZS.397)
HZS.429	*hxnYΔ::riboB*$^{+}$ *hxnR*^{c}*7 pabaA1 biA1 riboB2*	parental strain in genetic crosses with HZS.309, HZS.517, HZS.726, HZS.727 and HZS.783; metabolite analysis	this work (obtained by genetic cross of HZS.223 with HZS.395)
HZS.480	*hxnPΔ::riboB*$^{+}$ *hxnZΔ::pyroA*$^{+}$ *riboB2 pyroA4 nkuAΔ::argB*$^{+}$	growth test	this work (by transformation of the "uphxnZ-pyroA^{+}-downhxnZ" substitution cassette into HZS.221)
HZS.502	*hxnTΔ::pabaA*$^{+}$ *hxnYΔ::riboB*$^{+}$ (*riboB2 pabaA1*)	growth test	this work (obtained by genetic cross of HZS.222 with HZS.223)
HZS.517	*hxnWΔ::riboB*$^{+}$ *pantoB100 hxnR*^{c}*7* (*riboB2*)	growth test; metabolite analysis; parental strain in genetic crosses with HZS.427 and HZS.429	this work (obtained by genetic cross of HZS.393 and HZS.307)
HZS.534	*hxnXΔ::riboB*$^{+}$ *pantoB100 biA1 pabaA1* (*riboB2*) *in trans pDsRed-SKL-argB*$^{+}$ plasmid in 1 copy	parental strain in genetic crosses with HZS.568, HZS.726 and HZS.727	this work (obtained by genetic cross of HZS.305 and NA1322)
HZS.537	*hxnVΔ::riboB*$^{+}$ *hxnR*^{c}*7 pabaA1 yA2* (*riboB2 nkuΔ::argB*$^{+}$)	parental strain in genetic crosses with HZS.427, HZS.568; HZS.726 and HZS.727	this work (obtained by genetic cross of HZS.309 with HZS.143)
HZS.548	*hxnSΔ::pabaA*$^{+}$ *pabaA1 anA1 riboB2*	parental strain in genetic crosses with HZS.223	this work (obtained by genetic cross of HZS.397 and HZS.599)
HZS.558	*hxnSΔ::pabaA*$^{+}$ *hxnYΔ::riboB*$^{+}$ *anA1 pabaA1 riboB2*	growth test	this work (obtained by genetic cross of HZS.223 and HZS.548)
HZS.563	*nkuAΔ::argB*$^{+}$ *pabaA1 riboB2 pyroA4*	recipient strain for transformation experiment to obtain *hxnX* deletion mutants	this work (obtained from genetic cross of TN02 A21 with A148)

HZS.564	*nkuAΔ::argB⁺ pabaA1 riboB2 pyroA4*	recipient strain for transformation experiment to obtain *hxnS hxnT* double deletion mutant	this work (obtained from genetic cross of TN02 A21 with A148)
HZS.568	*hxnSTΔ::pabaA⁺ pabaA1 pyroA4 riboB2 nkuAΔ::argB⁺*	parental strain in genetic crosses with HZS.223, HZS.537 and HZS.623; recipient strain for transformation experiment to obtain *hxnSΔ hxnTΔ hxnR^{c}7* double deletion mutant	this work (by transformation of "ruphxnS-pabaA⁺-downhxnT" substitution cassette into HZS.564)
HZS.569	*hxnSTΔ::pabaA⁺ hxnYΔ::riboB⁺ pyroA4 pabaA1 riboB2* (*nkuAΔ::argB⁺*)	growth test; recipient strain for transformation experiment to obtain *hxnSΔ hxnTΔ hxnYΔ hxnR^{c}7* triple deletion mutant	this work (obtained from genetic cross of HZS.568 with HZS.223)
HZS.579	*hxnXΔ::riboB⁺ biA1 pabaA1* (*riboB2*)*; in trans pDsRed-SKL-argB⁺* plasmid in 1 copy*; in trans* pAN-HZS-13 plasmid is 7 copy	fluorescent microscopy	this work (by transformation of the pAN-HZS-13 plasmid into HZS.534)
HZS.582	*hxnMΔ::riboB⁺ hxnXΔ::riboB⁺ pantoB100 (riboB2)*	metabolite analysis	this work (obtained from genetic cross of HZS.293 and HZS.297)
HZS.584	*hxnMΔ::riboB⁺ hxnVΔ::riboB⁺ pabaA1 biA1 (riboB2)*	metabolite analysis	this work (obtained from genetic cross of HZS.294 and HZS.292)
HZS.588	*hxnMΔ::riboB⁺ hxnWΔ::riboB⁺ pabaA1 pantoB100 (riboB2)*	metabolite analysis	this work (obtained from genetic cross of HZS.393 with HZS.292)
HZS.592	*hxnTΔ::pabaA⁺ riboB2 pantoB100 (pabaA1)*	parental strain in genetic crosses with HZS.223	this work (obtained from genetic cross of HZS.98 with HZS.222)

HZS.599	*hxnSΔ::pabaA*$^{+}$ *pabaA1 riboB2*	growth test; parental strain in genetic crosses with HZS.397; recipient strain for transformation experiment to obtain *hxnS hxnT* double deletion mutant	[3]
HZS.614	*hxnRΔ::AfriboB*$^{+}$ *riboB2 pyroA4 nkuAΔ::argB*$^{+}$	parental strain in genetic crosses with HZS.281 and CS2638; metabolite analysis; mRNA expression analysis	this work (by transformation of the "uphxnR-AfriboB^{+}-downhxnR" substitution cassette into TN02 A21)
HZS.623	*hxnWΔ::riboB*$^{+}$ *riboB2 pabaA1*	parental strain in genetic crosses with HZS.568	this work (obtained by genetic cross of HZS.393 and HZS.123)
HZS.726	*hxnXΔ::riboB*$^{+}$ *riboB2 pabaA1 pyroA4 nkuAΔ::argB*$^{+}$	growth test; parental strain in genetic crosses with HZS.222, HZS.404, HZS.429 and HZS.537	this work (by transformation of the "uphxnX-riboB^{+}-downhxnX" substitution cassette into HZS.563)
HZS.727	*hxnXΔ::pabaA*$^{+}$ *pabaA1 riboB2 pyroA4 nkuAΔ::argB*$^{+}$	parental strain in genetic crosses with HZS.222, HZS.404, HZS.429 and HZS.537	this work (by transformation of the "uphxnX-pabaA^{+}-downhxnX" substitution cassette into HZS.563)
HZS.747	*hxnYΔ::riboB*$^{+}$ *hxnVΔ::riboB*$^{+}$ *hxnR*^{c}*7 pyroA4* (*riboB2 nkuAΔ::argB*$^{+}$)	metabolite analysis	this work (obtained by genetic cross of HZS.429 and HZS.309)
HZS.748	*hxnTΔ::pabaA*$^{+}$ *hxnVΔ::riboB*$^{+}$ *hxnR*^{c}*7 anA1 pabaA1* (*riboB2 nkuΔ::argB*$^{+}$)	metabolite analysis	this work (obtained by genetic cross of HZS.427 and HZS.537)
HZS.749	*hxnVWΔ::riboB*$^{+}$ *riboB2 hxnR*^{c}*7 pantoB100*	metabolite analysis	this work (by transformation of the "uphxnV-riboB^{+}-downhxnW" substitution cassette into HZS.404)
HZS.750	*hxnXWVΔ::riboB*$^{+}$ *riboB2 hxnR*^{c}*7 pantoB100*	metabolite analysis	this work (by transformation of the "uphxnV-riboB^{+}-downhxnX" substitution cassette into HZS.404)

HZS.751	*hxnXWΔ::riboB*$^{+}$ *riboB2 hxnR*^{c}*7 pantoB100*	metabolite analysis	this work (by transformation of the "uphxnW-riboB^{+}-downhxnX" substitution cassette into HZS.404)
HZS.783	*hxnXΔ::pabaA*$^{+}$ *hxnVΔ::riboB*$^{+}$ *hxnR*^{c}*7 pyroA4 pabaA1 nkuAΔ::argB*$^{+}$(*riboB2*)	metabolite analysis	this work (obtained by genetic cross of HZS.727 and HZS.537)
HZS.795	*hxnTΔ::pabaA*$^{+}$ *hxnYΔ::riboB*$^{+}$ *pantoB100 pabaA1 riboB2*	parental strain in genetic crosses with HZS.398	this work (obtained by genetic cross of HZS.223 and HZS.592 (derived from subsequent crosses of HZS.98 and HZS.222))
HZS.798	*hxnXΔ::riboB*$^{+}$ *hxnTΔ::pabaA*$^{+}$ *hxnR*^{c}*7 anA1 pabaA1* (*nkuAΔ::argB*$^{+}$ *riboB2*)	metabolite analysis	this work (obtained by genetic cross of HZS.427 and HZS.726)
HZS.810	*hxnXΔ::pabaA*$^{+}$ *hxnYΔ::riboB*$^{+}$ *hxnR*^{c}*7 pyroA4 biA1 riboB2 pabaA1* (*nkuAΔ::argB*$^{+}$)	metabolite analysis	this work (obtained by genetic cross of HZS.429 and HZS.726)
HZS.812	*hxnXΔ::pabaA*$^{+}$ *hxnR*^{c}*7 pabaA1* (*riboB2 nkuAΔ::argB*$^{+}$)	metabolite analysis	this work (obtained by genetic cross of HZS.537 and HZS.727)
HZS.892	*hxnSΔ::pabaA*$^{+}$ *pabaA1 hxnTΔ::riboB*$^{+}$*riboB2*	growth test	this work (by transformation of the "uphxnT-riboB^{+}-downhxnT" substitution cassette into HZS.599)
HZS.894	*hxnWΔ::riboB*$^{+}$ *hxnTΔ::pabaA*$^{+}$ *hxnR*^{c}*7* (*riboB2 pabaA1*)	metabolite analysis	this work (obtained by genetic cross of HZS.427 and HZS.517)
HZS.898	*hxnYΔ::riboB*$^{+}$ *hxnWΔ::riboB*$^{+}$ *pantoB100 hxnR*^{c}*7* (*riboB2*)	metabolite analysis	this work (obtained by genetic cross of HZS.429 and HZS.517)
HZS.899	*hxnXΔ::pabaA*$^{+}$ *hxnVΔ::riboB*$^{+}$ *hxnTΔ::pabaA*$^{+}$ *pyroA4 hxnR*^{c}*7* (*pabaA1 riboB2*)	metabolite analysis	this work (obtained by genetic cross of HZS.427 and HZS.783)
HZS.901	*hxnXΔ::pabaA*$^{+}$ *hxnVΔ::riboB*$^{+}$ *hxnYΔ::riboB*$^{+}$ *hxnR*^{c}*7* (*pabaA1 riboB2*)	metabolite analysis	this work (obtained by genetic cross of HZS.429 and HZS.783)
HZS.902	*hxnVWΔ::riboB*$^{+}$ *hxnTΔ::pabaA*$^{+}$ *pantoB100 hxnR*^{c}*7* (*pabaA1*)	metabolite analysis	this work (obtained by genetic cross of HZS.427 and HZS.749)

HZS.903	*hxnTΔ::pabaA*$^+$ *hxnYΔ::riboB*$^+$ *pantoB100 hxnR*^{c}*7 pabaA1 riboB2*	metabolite analysis	this work (obtained by genetic cross of HZS.398 and HZS.795)
HZS.904	*hxnXWΔ::riboB*$^+$ *hxnTΔ::pabaA*$^+$ *pantoB100 hxnR*^{c}*7* (*pabaA1 riboB2*)	metabolite analysis	this work (obtained by genetic cross of HZS.427 and HZS.751)
HZS.911	*hxnSTΔ::pabaA*$^+$ *pabaA1 riboB2 pyroA4* (*nkuAΔ::argB*$^+$) + *hxnR*^{c}*7 - pyroA*$^+$ in *in trans* pAN-HZS-17 plasmid in 2 copy	metabolite analysis	this work (by transformation of the pAN-HZS-17 plasmid into HZS.568)
HZS.912	*hxnSTΔ::pabaA*$^+$ *pabaA1 hxnYΔ::riboB*$^+$ *riboB2 pyroA4* (*nkuAΔ::argB*$^+$) + *hxnR*^{c}*7 - pyroA*$^+$ in *in trans* pAN-HZS-17 plasmid in 1 copy	metabolite analysis	this work (by transformation of the pAN-HZS-17 plasmid into HZS.569)

Parenthetic loci indicate alleles that were present in one of the parents of a cross but have not been tested in the progeny.

Explanation of mutant alleles, which are not described in the text: *nkuAΔ* is the deletion of *nkuA* that is essential for non-homologous end joining of DNA in double-strand break repair [23], acet$^-$ is mutation resulting acetate requirement, *fpaD43* is a mutation in *fpaD* resulting p-fluorophenylalanine resistance [26], *veA1* is a mutation in the *veA* gene resulting profuse conidiation regardless of the presence or absence of light [22], *yA2* is mutation in *yA* resulting yellow conidia [27] and *pyr4* is gene for orotidine 5'-phosphate carboxylase in *N. crassa* [28], which complements *pyrG89* allele of *A. nidulans*. Other gene symbols refer to auxotrophies: *argB2*, arginine; *biA1*, biotin; *pabaA1*, p-aminobenzoic acid; *pantoB100*, pantothenic acid; *pyroA4*, pyridoxine; *pyrG89* uracil or uridine; *riboB2*, riboflavin and *anA1*, thiamine. *AfriboB*$^+$ is *riboB* selection marker gene from *A. fumigatus* used for gene replacement. *nicB8* is a mutation in a NA biosynthetic pathway gene (*nicB*) resulting NA auxotrophy. *hxnR*^{c}*7* is a mutation in the *hxnR* gene resulting constitutive expression of *hxnR* (and all *hxn* genes) without induction [3].

Supplementary Table 4. Results of protein modelling and superpositioning of these models with their closest known structural homologs

	HxnY	HxnT	HxnX	HxnW	HxnV	HxnM	HxnN
	**Quality assessment of the protein models**						
C-score of i.model[1]	0.94	1.03	-0.3	1.21	0.33	1.24	0.35
estim. TM-score of i.model[2]	0.84±0.08	0.85±0.08	0.67±0.12	0.88±0.07	0.76±0.1	0.88±0.07	0.76±0.10
estim. RMSD of i.model[3]	4.6±3.0 Å	4.6±3.0 Å	7.8±4.4 Å	3.4±2.4 Å	7.1±4.1 Å	3.7±2.5 Å	6.7±4.0 Å
TM-score of ref.model to i.model[4]	0.9893	0.9885	0.9834	0.9934	0.9943	0.9923	0.9902
RMSD of ref.model to i.model[5]	0.748 Å	0.941 Å	1.096 Å	0.488 Å	0.700 Å	0.581 Å	0.856 Å
RAMA[6], % of AAs in favoured region	89.3% (268 AAs)	83.5% (269 AAs)	76.6% (305 AAs)	92.3% (205 AAs)	79.1% (432 AAs)	89.1% (230 AAs)	85.1% (393 AAs)
RAMA[6], % of AAs in allowed region	9.6% (29 AAs)	15.8% (51 AAs)	19.6% (78 AAs)	6.8% (15 AAs)	18.1% (99 AAs)	10.1% (3 AAs)	14.7% (68 AAs)
RAMA[6], % of AAs in disallowed region	1.0% (3 AAs)	0.6% (2 AAs)	3.8% (15 AAs)	0.9% (2 AAs)	2.7% (15 AAs)	0.8% (2 AAs)	0.2% (1 AA)
number of non-Pro, non-Gly residues	300 AAs	322 AAs	398 AAs	222 AAs	546 AAs	258 AAs	462 AAs
	**Quality assessment of the superpositioning of the modelled Hxn proteins with their corresponding closest known structural homologs (by built-in Matchmaker of UCSF Chimera 1.14)**						
structural homologues used for superpositioning (PDB number; reference; host organism[7]; enzyme name[8])	5c3q; [16]; *N. crassa*; T-7-H	1oya; [14]; *S. pastorianus;* SpOYE1	5eow; [29]; *P. putida*; NicC	3awd; [15]; *G. oxydans*; Gox2181	1pn0; [10]; *T. cutaneum*; PHOX; and 2dkh; [30]; *C. testosteroni*; 3HBH	3qbu; [18]; *H. pylori*; HpPgdA	2vyA; [21]; *R. norvegicus*; FAAH1
Sequence alignment scores[9]	619.0	807.5	628.3	516.8	with 1pn0: 985.8<br>with 2dkh: 1040.2	1156.1	1027.7
Structure RMSDs between pruned atom pairs in superposition	1.056 Å (229 pruned atom pairs)	0.793 Å (276 pruned atom pairs)	0.733 Å (282 pruned atom pairs)	0.760 Å (217 pruned atom pairs)	with 1pn0: 1.100 Å (400 pruned atom pairs)<br>with 2dkh: 0.628 Å (469 pruned atom pairs)	0.517 Å (289 pruned atom pairs)	0.704 Å (421 pruned atom pairs)
Structure RMSDs between all atom pairs in superposition	4.082 Å (across all 305 pairs)	5.551 Å (across all 360 pairs)	1.993 Å (across all 350 pairs)	1.946 Å (across all 249 pairs)	with 1pn0: 3.011 Å (across all 552 pairs) and<br>with 2dkh: 3.342 Å (across all 565 pairs)	0.536 Å (across all 290 pairs)	2.602 Å (across all 509 pairs)
modelled interacting molecules in the PDB model[10]	Ni^{2+}; αKG[10]; TDR[10]	FMN[10]; HBA[10]	FAD[10]	-	FAD[10]; IPH[10] (for 1pn0); 3HB[10] (for 2dkh)	Zn^{2+}	PF7[10]
	**Putative enzyme activities and their substrate**						
proposed enzyme activity	hydroxylation	alkene reduction, saturation of pyridine ring	conversion of 6-NA to 2,5-DP	dehydrogenation of –OH group	conversion of 2,5-DP to tri-hydroxylated pyridine	opening of a saturated pyridine (piperidine) ring between C-N	deamination of the opened saturated pyridine (piperidine) ring
possible substrates of enzymes	6-NA-derivative with closed pyridine ring	6-NA derivative with closed pyridine ring	6-NA	polyhydroxylated pyridine ring	2,5-DP	saturated pyridine (piperidine) ring	aliphatic NA derived catabolite with amide group

[1,2,3,4 and 5] C-score of initial model, estimated TM-score of initial model, estimated RMSD of initial model, TM-score of refined model to initial model and RMSD of refined model to initial model, respectively. Initial models were obtained by I-Tasser [31]; Refined models were obtained by ModRefiner [32].

[6] RAMA: Ramachandran plot analysis results on non-Proline and non-Glycine regions derived from using Procheck server (https://servicesn.mbi.ucla.edu/PROCHECK/)

[7] Organisms: *N. crassa*: *Neurospora crassa, S. pastorianus*: *Saccharomyces pastorianus*; *P. putida*: *Pseudomonas putida*, *G. oxydans: Gluconobacter oxydans*, *T. cutaneum*: *Trichosporon cutaneum*; *C. testosteroni*: *Comamonas testosteroni*; *H. pylori*: *Helicobacter pylori*; *R. norvegicus*: *Rattus norvegicus*.

[8] Proteins: T-7-H: Thymine-7-hydroxylase, SpOYE1: Old Yellow Enzyme of *S. pastorianus*, NicC: 6-hydroxynicotinic acid 3-monooxygenase; Gox2181: polyol dehydrogenase of C4-C8 dihydroxy alcohols, PHOX: phenol hydroxylase (phenol 2-monooxygenase), 3HBH: 3-hydroxybenzoate hydroxylase; HpPgdA: proposed cyclic imidase of *H. pylori*; FAAH1: fatty acid amide hydrolase 1 of AS (amidase signature) amidases (with Ser-Ser-Lys catalytic triad).

[9] Computing secondary structure assignments of superimposed models used ksdssp (Kabsch and Sander Define Secondary Structure of Proteins) with the parameters: -0.5 energy cutoff; minimum helix length 3; minimum strand length 3. Sequence alignment scores were obtained by Matchmaker (built in UCSF Chimera 1.14) with the following parameter values: chain pairing: bb; alignment algorithm: Needleman-Wunsch using BLOSUM-62 matrix; ss (secondary structure) fraction: 0.3; gap opening penalties (HH/SS/other)(HH: intra Helix; SS: intra Strand) 18/18/6, gap extension penalty: 1; ss scoring matrix: (O, S): -6 (H, O): -6 (H, H): 6 (S, S): 6 (H, S): -9 (O, O): 4 (H is Helix, S is Strand, O is Other); iteration cutoff: 2.

[10] Modelled interacting molecules: Ni^{2+} (nickel ion), αKG (α-ketoglutarate), TDR (thymine), FMN (flavin mononucleotide), HBA: *p*-hydroxybenzaldehyde; FAD (flavin adenine dinucleotide), IPH (phenol), 3HB (3-hydroxybenzoic acid), Zn^{2+} (zinc ion), PF7 (4-(quinolin-3-ylmethyl)piperidine-1-carboxylic acid).

Supplementary Table 5. List of primers used in this study.

Primer names	Sequence	Primer number
**Primers used for gene deletions**		
***hxnP* deletion strain**		
hxnP upst frw	5'- caccgagctgtagctcacctgcttgatg -3'	**1.**
hxnP upst rev	5'- gtggagatttataaacggttctgtttgg -3'	**2.**
hxnP ribo chim frw	5'- *ccaaacagaaccgtttataaatctccac*cgtacgtagtgtagattcaggcacattgaagcg -3'	**3.**
hxnP ribo chim rev	5'- *gacccagtccctacattctgctgtctctg*ggaaaactgccatgactactaggtggtgctatc -3'	**4.**
hxnP downst frw	5'- cagagacagcagaatgtagggactgggtc -3'	**5.**
hxnP downst rev	5'- cgtaaaccgtctcgtctcgtctgctgacac -3'	**6.**
hxnP upst nest frw	5'- ctcacgcttgtcgagttcgatttccatgatg -3'	**7.**
hxnP downst nest rev	5'- aagcttgatcagcacacagtggaatagctg -3'	**8.**
***hxnY* deletion strain**		
hxnY upst frw	5'- catatcaaatcagagaggagtctatactg -3'	**9.**
hxnY upst rev	5'- ggatactcaacgattactgctgtttagg -3'	**10.**
hxnY ribo chim frw	5'- *cctaaacagcagtaatcgttgagtatcc*cgtacgtagtgtagattcaggcacattgaagcg -3'	**11.**
hxnY ribo chim rev	5'- *cattatgctagcttacatgacaacaagtac*ggaaaactgccatgactactaggtggtgctatc -3'	**12.**
hxnY downst frw	5'- gtacttgttgtcatgtaagctagcataatg -3'	**13.**
hxnY downst rev	5'- gttgtgtatctcggtgcgaggctctggtac -3'	**14.**
hxnY upst nest frw	5'- gcacgagacacgtcggaatgtatgcaccag -3'	**15.**
hxnY downst nest rev	5'- ctctgacacgactcctagatagcagcatg -3'	**16.**
***hxnZ* deletion strain**		
hxnZ upst frw	5'- ctactgagcgagagcataatccgtgccgag -3'	**17.**
hxnZ upst rev	5'- cctaataatgataagtgaggccagacgctg -3'	**18.**
hxnZ ribo chim frw	5'- *cagcgtctggcctcacttatcattattag*gcgtacgtagtgtagattcaggcacattgaagcg -3'	**19.**
hxnZ ribo chim rev	5'- *ccacttatcagcacaaactctctgacacg*ggaaaactgccatgactactaggtggtgctatc -3'	**20.**
hxnZ downst frw	5'- cgtgtcagagagtttgtgctgataagtgg -3'	**21.**
hxnZ downst rev	5'- gttccatcgtacagcatgctgactgcatac -3'	**22.**
hxnZ upst nest frw	5'- cattgatcatgagccgctcgatcaacatac -3'	**23.**
hxnZ downst nest rev	5'- agcagcaggttccaatgactcgaagtgac -3'	**24.**
hxnZ pyro chim frw	5'- *cagcgtctggcctcacttatcattattagg*cagttgagcctgagaccaatgaatac -3'	**25.**
hxnZ pyro chim rev	5'- *ccacttatcagcacaaactctctgacacg*gtcagtttagtagctgaagcgttcttattag -3'	**26.**
hxnZ upst nest frw2	5'- gaatggagaacggagaatggagactg -3'	**27.**
***hxnT* deletion and *hxnS-hxnT* double deletion**		
hxnT upst frw	5'- ctgtgcagtcattgcgtcatctgcatacac -3'	**28.**
hxnT upst rev	5'- cgactgtctcagtagactacgtcatgagc -3'	**29.**
hxnT paba chim frw	5'- *gctcatgacgtagtctactgagacagtcg*gcacatagctattacacgtatgtttgagac -3'	**30.**
hxnT paba chim rev	5'- *ctatctgtattctgtgtcgtagtattcatg*gtagtttgcttgaatggctaacgaggcattg -3'	**31.**
hxnT downst frw	5'- gaatactacgacacagaatacagatagac -3'	**32.**
hxnT downst rev	5'- catagtcttaaccacgagacgatcagtaac -3'	**33.**
hxnT upst nest frw	5'- cgtcgtcctttcgctttgcctgtttgtatg -3'	**34.**
hxnT downst nest rev	5'- tctgttctactacaggcagcgagttttgtc -3'	**35.**
hxnS r up frw	5'- gtgtactcgttcatcacgccaaag -3'	**36.**

hxnS r up rev	5'- cttgctgttggagacgacttgg -3'	**37.**
hxnS r paba chim frw	5'- *ccaagtcgtctccaacagcaag*gcacatagctattacacgtatgtttgagac -3'	**38.**
hxnS r up nest frw	5'- cagttgaggcatcttgatgtgag -3'	**39.**
hxnS irup frw	5'- gatgtccaggaatcggcagctatac -3'	**40.**
hxnT upst rev2	5'- gtgcatacatcacgactgattggagttg -3'	**41.**
hxnT ribochim frw	5'- *caactccaatcagtcgtgatgtatgcac*cgtacgtagtgtagattcaggcacattgaagcg -3'	**42.**
hxnT ribochim rev	5'- *ctccggtcatacttggtcaactcgatac*ggaaaactgccatgactactaggtggtgctatc -3'	**43.**
hxnT down frw2	5'- gtatcgagttgaccaagtatgaccggag -3'	**44.**
paba int frw2	5'- gagacatccatcactttaccatcgcccg -3'	**45.**
***hxnR* deletion**		
hxnR upst frw	5'- cagattcgaacgtacctggtttggtc -3'	**46.**
hxnR upst rev	5'- gtatgtgcaggcgtgtttcttcatc -3'	**47.**
hxnR Afuribo chim frw	5'- *gaagaaacacgcctgcacatac*caggagtctggctcgtttgatc -3'	**48.**
hxnR Afuribo chim rev	5'- *tcagcaagaccttcatag*cttgcgaccgtctgatctagattac -3'	**49.**
hxnR downst frw	5'- atgaaggtcttgctgaatcttggtg -3'	**50.**
hxnR downst rev	5'- ctcttgcactgaccttggtttcac -3'	**51.**
hxnR upst nest frw	5'- gtccggttggcaagacaaagactc -3'	**52.**
hxnR downst nest rev	5'- accaactctgactcgacaatgctcg -3'	**53.**
hxnR prom frw	5'- tttttttgatatccatctggtgcatacattccgacgtg -3'	**54.**
hxnR prom rev	5'- tttttttgatatcgcgtgaaggctttggagcag -3'	**55.**
***hxnX* deletion**		
hxnX upst frw	5'- cagcgtcaagtctcatatctatactg -3'	**56.**
hxnX upst rev	5'- ggaattaggaaggatctggataccgg -3'	**57.**
hxnX ribo chim frw	5'- *ccggtatccagatccttcctaattcc*cgtacgtagtgtagattcaggcacattgaagcg -3'	**58.**
hxnX ribo chim rev	5'- *gtgtatcatcttctcgctcacatctg*ggaaaactgccatgactactaggtggtgctatc -3'	**59.**
hxnX paba chim frw	5'- *ccggtatccagatccttcctaattcc*gcacatagctattacacgtatgtttgagac -3'	**60.**
hxnX paba chim rev	5'- *gtgtatcatcttctcgctcacatctg*gtagtttgcttgaatggctaacgaggcattg -3'	**61.**
hxnX downst frw	5'- cagatgtgagcgagaagatgatacac -3'	**62.**
hxnX downst rev	5'- gagacttgtactcttatcgttcgc -3'	**63.**
hxnX upst nest frw	5'- caccttctttgtacccgtgctctg -3'	**64.**
hxnX downst nest rev	5'- aagcttgatcagcacacagtggaatagctg -3'	**65.**
***hxnW* deletion**		
hxnW upst frw	5'- actcctccacccaccgtcttg -3'	**66.**
hxnW upst rev	5'- gtgttcttggcgagcgatgcc -3'	**67.**
hxnW ribo chim frw	5'- *ggcatcgctcgccaagaacac*cgtacgtagtgtagattcaggcacattgaagcg -3'	**68.**
hxnW ribo chim rev	5'- *ctcgcagtggtacagtgctg*ggaaaactgccatgactactaggtggtgctatc -3'	**69.**
hxnW downst frw	5'- cagcactgtaccactgcgag -3'	**70.**
hxnW downst rev	5'- gatcagactagtgctagtggttactaac -3'	**71.**
hxnW upst nest frw	5'- cagcgtcaagtctcatatctatactg -3'	**72.**
hxnW downst nest rev	5'- ccatgaaatggattgtaaacctcaag -3'	**73.**
***hxnV* deletion**		
hxnV upst frw	5'- acttctcaaactgctcgcgtcc -3'	**74.**
hxnV upst rev	5'- caagacggtgggtggaggagt -3'	**75.**
hxnV ribo chim frw	5'- *actcctccacccaccgtcttg*cgtacgtagtgtagattcaggcacattgaagcg -3'	**76.**
hxnV ribo chim rev	5'- *cacgaccaaggcttcgcaaac*ggaaaactgccatgactactaggtggtgctatc -3'	**77.**
hxnV downst frw	5'- gtttgcgaagccttggtcgtg -3'	**78.**

hxnV downst rev	5'- ctgagacgtcagtgaggacat -3'	**79.**
hxnV upst nest frw	5'- caagggtgaactgcttcgctagg -3'	**80.**
hxnV downst nest rev	5'- cgaaattgtttctctgcaactggg -3'	**81.**
***hxnM* deletion**		
hxnM upst frw	5'- ctcattgtagtagcattcattgtcgc -3'	**82.**
hxnM upst rev	5'- cgacaccgtaggatacgagaac -3'	**83.**
hxnM ribo chim frw	5'- *gttctcgtatcctacggtgtcg*cgtacgtagtgtagattcaggcacattgaagcg -3'	**84.**
hxnM ribo chim rev	5'- *gctcttaaactcatcgcacatctg*ggaaaactgccatgactactaggtggtgctatc -3'	**85.**
hxnM downst frw	5'- cagatgtgcgatgagtttaagagc -3'	**86.**
hxnM downst rev	5'- ggcgaaacttgagtacgagtg -3'	**87.**
hxnM upst nest frw	5'- cgaatcccgcaaagcattctg -3'	**88.**
hxnM downst nest rev	5'- gatgtccgggtattcggtgcag -3'	**89.**
***hxnN* deletion**		
hxnN upst frw	5'- cgtagaggctgttttcatgtcctg -3'	**90.**
hxnN upst rev	5'- cgtttctttgcgactgtctgctc -3'	**91.**
hxnN ribo chim frw	5'- *gagcagacagtcgcaaagaaacg*cgtacgtagtgtagattcaggcacattgaagcg -3'	**92.**
hxnN ribo chim rev	5'- *ctagccgtttcccaattacctgc*ggaaaactgccatgactactaggtggtgctatc -3'	**93.**
hxnN downst frw	5'- gcaggtaattgggaaacggctag -3'	**94.**
hxnN downst rev	5'- gatgcacagattgtgaaacgattg -3'	**95.**
hxnN upst nest frw	5'- cggagaatatgtggttcgagc 3'	**96.**
hxnN downst nest rev	5'- gctcttaaactcatcgcacatctg -3'	**97.**
**Gene specific primers**		
hxnR frw	5'- caagcgagactttccagacctagac -3'	**106.**
hxnR rev	5'- aatgctccgtatcccacatgaaacac -3'	**107.**
hxnP frw	5'- gttgatcgagcggctcatgatcaatgagtc -3'	**108.**
hxnP rev	5'- gcactcagaacaccagcgatattgcccag -3'	**109.**
hxnT frw	5'- ctttgcgctgcagcaccgtgttgtccaag -3'	**110.**
hxnT rev	5'- ctattgagcgaaagggtagtccgtatag -3'	**111.**
hxnY frw	5'- caagcttgtacagcaagtacgcgaatcctg -3'	**112.**
hxnY rev	5'- cagtttggcagacacttgcacacatggtctg -3'	**113.**
hxnZ frw	5'- cagatcatcatggaaatcgaactcgacaag -3'	**114.**
hxnZ rev	5'- ctgactggatgcagtagttgcggtaggtc -3'	**115.**
hxnX frw	5'- cgacagctattgctgcggac -3'	**116.**
hxnX rev	5'- ctgagacgtcagtgaggacat -3'	**117.**
hxnW frw	5'- gcagatcgtggtgttcttcgtag -3'	**118.**
hxnW rev	5'- ctcgcagtggtacagtgctg -3'	**119.**
hxnV frw	5'- cagcgtcaagtctcatatctatactg -3'	**120.**
hxnV rev	5'- cagagcacgggtacaaagaaggtg -3'	**121.**
hxnM frw	5'- caagacctaccgcatgattacagac -3'	**122.**
hxnM rev	5'- gtttattgatgtgttcgatgatcctaac -3'	**123.**
hxnN frw	5'- cgaatcccgcaaagcattctg -3'	**124.**
hxnN rev	5'- ctctgtccacagggaaagtgatggc -3'	**125.**
hxnT prom frw2	5'- gattctcggtcatgtagagcagg -3'	**126.**
hxnT prom rev2	5'- gcctgcatggacaagctatttag -3'	**127.**
hxnV AS frw	5'- cagcgtcaagtctcatatctatactg -3'	**128.**
hxnV AS rev	5'- cagagcacgggtacaaagaaggtg -3'	**129.**
hxnW AS frw	5'- gcagatcgtggtgttcttcgtag -3'	**130.**
hxnW AS rev	5'- ctcgcagtggtacagtgctg -3'	**131.**
**For cloning**		
pyroA HindIII frw	5'-tttttttaagcttcagttgagcctgagaccaatgaatac -3'	**132.**
pyroA HindIII rev	5'-tttttttaagcttgtcagtttagtagctgaagcgttcttattag -3'	**133.**
AN11197 prom	5'-tttttttgcggccgccatctggtgcatacattccgacgtg -3'	**134.**

NotI frw		
AN11197 term NheI rev	5'-ttttttt<u>gctagc</u>cgccggtgagaaacttataacagac -3'	**135.**
pgpd int2 frw	5'- catgaatctgaggactgcaatcgc -3'	**136.**
trpC term rev	5'- cgatcttatatccagattcgtcaagctg -3'	**137.**
1pGpd int frw	5'- cagtatattcatcttcccatccaagaac -3'	**138.**
10GFP linker hmgB rev	5'- *atcaagatcgactgtatcaataag*cttgtacagctcgtccatgccgtg -3'	**139.**
linker kim hxnX frw	5'- *acaagcttattgatacagtcgatcttgat*atgcccatcccagttgcagagaaac -3'	**140.**
5GFP NcoI start frw	5'- tttttttccatggtgagcaagggcgaggagc -3'	**141.**
hxnX NotI rev	5'- ttttttt<u>gcggccgc</u>tcataaccgcgatgctacctgttc -3'	**142.**
**Sequencing primers**		
AN11197 2F	5'- gcacgcttatcgtctccactg -3'	**148.**
AN11197 800F	5'- gtatgatgccaatacagtaaagctacc -3'	**149.**
AN11197 1375F	5'- cttctcccgcttcaatactacatacc -3'	**150.**
AN11197 1958 F	5'- agagatacagaacatgcatttcttccc -3'	**151.**
**RT-qPCR**		
actin ReTi frw	5'- ggtatcatgatcggtatggg -3'	**152.**
actin ReTi rev	5'- tatctgagtgtgaggatacca -3'	**153.**
hxnR ReTi frw	5'- cggcttctgttctactacagg -3'	**154.**
hxnR ReTi rev	5'- cagtctaggtctggaaagtctc -3'	**155.**
hxnX ReTi frw	5'- cttgtatcatctccacgacgg -3'	**156.**
hxnX ReTi rev	5'- ggctaaacactctccctctg -3'	**157.**
hxnS Reti frw	5'- gagcatttctatcttgagacga -3'	**158.**
hxnS Reti rev	5'- ccattgtgttctgggtactg -3'	**159.**
AN5650 ReTi frw	5'- atgtctgctgttatctatctgctc -3'	**160.**
AN5650 ReTi rev	5'- gccaatcctccttaccttcc -3'	**161.**
actin ReTi frw2	5'- accatgtaccctggtatctc -3'	**162.**
actin ReTi rev2	5'- ggaggagcaatgatcttgac -3'	**163.**

Underlined letters in the primer sequences or in the primer names refer to the restriction sites designed within.

Italic letters at the 5' end refer to the chimeric nature of the primer.

SUPPELEMENTARY METHODS
for

A complete nicotinate degradation pathway in the microbial eukaryote *Aspergillus nidulans*

Eszter Bokor[1], Judit Ámon[1], Mónika Varga[1], András Szekeres[1], Zsófia Hegedűs[1], Tamás Jakusch[2], Zsolt Szakonyi[3], Michel Flipphi[4], Csaba Vágvölgyi[1], Attila Gácser[5,6], Claudio Scazzocchio[7,8]* and Zsuzsanna Hamari[1]*
[1]University of Szeged Faculty of Science and Informatics, Department of Microbiology, Szeged, Hungary
[2]University of Szeged Faculty of Science and Informatics, Department of Inorganic and Analytical Chemistry, Szeged, Hungary
[3]University of Szeged Faculty of Pharmacy, Institute of Pharmaceutical Chemistry, Szeged, Hungary
[4] Institute de Génétique et Microbiologie, Université Paris-Sud, Orsay, France
[5] HCEMM-USZ Fungal Pathogens Research Group, University of Szeged Faculty of Science and Informatics, Department of Microbiology, Szeged, Hungary
[6] MTA-SZTE "Lendület" Mycobiome Research Group, University of Szeged, Szeged, Hungary
[7] Section of Microbiology, Department of Infectious Diseases, Imperial College, London, United Kingdom
[8] Université Paris-Saclay, CEA, CNRS, Institute for Integrative Biology of the Cell (I2BC), 91198, Gif-sur-Yvette, France
Present address of M.F.: Department of Biochemical Engineering, Faculty of Science and Technology, University of Debrecen, Debrecen, Hungary
* Corresponding authors:
hamari@bio.u-szeged.hu,
c.scazzocchio@imperial.ac.uk

Content:

Supplementary Methods 1. Deletion of the *hxnR/T/Y/Z/P/V/W/X/M/N* genes by the transformation of gene-substitution cassettes constructed with Double-Joint PCR. This paragraph includes:

Supplementary Table 6: Detailed summary of construction and checking of gene deletion strains.

Supplementary Fig. 11: Southern blot analysis of gene-deletions in transformed strains.

Supplementary Methods 2. Introduction of the dominant *hxnR^{c}7* allele *in trans* in the *hxnR$^+$ hxnSΔ-hxnTΔ* double deletion and *hxnR$^+$ hxnSΔ-hxnTΔ-hxnYΔ* triple deletion strains.

This paragraph includes:

Supplementary Fig. 12: Schematic presentation of the original and resulting transformation vectors used for the construction of the *hxnR^{c}7* expression strains.

Supplementary Methods 3. Construction of Gfp-HxnX expressing strains

This paragraph includes:

Supplementary Fig. 13: Schematic representation of the original and resulting transformation vectors used for the construction of the *gfp-hxnX* expression strains.

Supplementary Methods 1. Deletion of the *hxnR/T/Y/Z/P/V/W/X/M/N* genes by the transformation of gene-substitution cassettes constructed with Double-Joint PCR.

Deletion of the *hxn* cluster genes was carried out as described previously [33]. The tripartite transformation cassettes (composed from ("A", "B" and "C" components) were constructed with Double-Joint PCR (DJ-PCR) [34]. While the "A" and "C" components drive homologous recombination, the "B" component served as selection marker. The *riboB* and/or *pabaA* and/or *pyroA* from *A. nidulans* or the *riboB* orthologue from *A. fumigatus* (Afu1g13300) were used as selection marker genes for gene-replacements. For *hxnY/P/Z/V/W/X/M* and *hxnN* deletion the *riboB* gene from *A. nidulans* was used; for the *hxnR* deletion the *riboB* gene from *A. fumigatus* was used; for the *hxnX, hxnT* deletions and the *hxnS-hxnT* double deletion, the *pabaA* gene from *A. nidulans* was used; for the *hxnZ* deletion, the *pyroA* gene from *A. nidulans* was used as the selection marker gene. Construction of the gene-replacement cassettes involved the amplification of the upstream and downstream flanking regions ("A" and "C" components of the cassette, respectively) of the targeted genes by using specific primers and the amplification of the selection marker gene ("B" component) by using chimeric primers. The 5' moieties of the forward chimera primers were specific for the 3' end of the "A" components; the 5' moieties of the reverse chimera primers were specific for the 5' end of the "C" components. Assembly of the "A", "B" and "C" components were carried out in a PCR reaction where "A"-component-specific nested forward and "C"-component-specific nested reverse primers were used. All the primers used and the sizes of PCR products for the construction of each gene-replacement cassettes are described below, in Supplementary Table 6. The recipient strains used for transformation, the result of PCR-based pre-selection (by using targeted gene-specific primer pairs) of transformants, and the Southern hybridization strategy for the analysis of the transformants are also listed in Supplementary Table 6. The Southern blot hybridization was carried out on restriction-enzyme-digested total DNA extracts of transformant strains and wild-type or recipient control strains with "A" or "C" component derived DNA probes using DIG-DNA labeling and detection kit (Roche) (details are shown in Supplementary Table 6 and Supplementary Fig. 11). Those transformant strains, which underwent the expected gene-replacement and were free from ectopic integration of the gene-replacement cassette, were used for nicotinate utilization tests (selected transformants are marked on Supplementary Table 6 and Supplementary Fig. 11). The co-segregation of the selection marker genes with the single deletions were tested for each selected deletion strains by analysis of the progeny of genetic crosses. The genetic crosses carried out are listed in Supplementary Table 6.

SupplementaryTable 6. Detailed summary of construction and checking of gene deletion strains.

**Targeted gene**	**Construction of gene substitution casettes by DJ-PCR**						**Southern analysis strategy**					**Selected transformant strains**	**Co-segregation tests by genetic crosses**
	forward + reverse primers[1] and product size				Recipient strain[1]	Primers for PCR tests[2]	Restriction enzymes[3]	Primers[1] for DNA probes	Controll size	Gene replacement size	Corres-ponding image*	HZS collection number	Strains used for crossing
	"A" component	"B" component	"C" component	Assembly of "A", "B", "C"									
hxnP[4]	1+2 (2,435 bp)	3+4 (2,264 bp)	5+6 (2,438 bp)	7+8 (5,878 bp)	TN02 A21	108+109	EcoRI	1+2	5,752 bp	3,240 bp	*A*	221	HZS.399, CS2638
hxnY[4]	9+10 (2,517 bp)	11+12 (2,266 bp)	13+14 (2,566 bp)	15+16 (6,396 bp)	HZS.120	112+113	BamHI	9+10	13,837 bp	4,415 bp	*B*	223	HZS.395
hxnZ[4]	17+18 (2,532 bp)	19+20 (2,266 bp)	21+22 (2,530 bp)	23+24 (6,584 bp)	TN02 A21	114+115	BamHI	17+18	13,887 bp	5,904 bp	*C*	226	HZS.399, CS2638
hxnT[5]	28+29 (2,666 bp)	30+31 (3,905 bp)	32+33 (2,645 bp)	34+35 (7,535 bp)	HZS.120	110+111	EcoRI	32+33	16,703 bp	3,568 bp	*D*	222	HZS.397
hxnT[6]	40+41 (2,219 bp)	42+43 (2,208 bp)	44+33 (2,748 bp)	45+35 (6,260 bp)	HZS.599	110+111	EcoRI	44+33	5,340 bp	3,560 bp	*E*	892	-
hxnR[7]	46+47 (3,204 bp)	48+49 (2,607 bp)	50+51 (2,787 bp)	52+53 (7,611 bp)	TN02 A21	106+107	XbaI	54+55	8,557 bp	5,729 bp	*F*	614	HZS.120
hxnX[4]	56+57 (2,849 bp)	58+59 (2,208 bp)	62+63 (2,551 bp)	64+65 (6,861 bp)	HZS.563	116+117	SacI	56+57	6,195 bp	10,055 bp	*G*	726	HZS.222, HZS.404, HZS.429, HZS.537
hxnX[5]	56+57 (2,849 bp)	60+61 (3,846 bp)	62+63 (2,551 bp)	64+65 (8,499 bp)	HZS.563	116+117	SacI	56+57	7,379 bp	10,055 bp	*G*	727	HZS.222, HZS.404, HZS.429, HZS.537
hxnV[4]	74+75 (2,030 bp)	76+77 (2,208 bp)	78+79 (2,406 bp)	80+81 (5,202 bp)	TN02 A21	120+121	EcoRI	74+75	4,342 bp	2,432 bp	*H*	294	FGSCA872, HZS.292
hxnW[4]	66+67 (2,662 bp)	68+69 (2,208 bp)	70+71 (3,953 bp)	72+73 (5,948 bp)	HZS.267	118+119	XbaI	66+67	8,821 bp	4014 bp	*I*	393	HZS.123, HZS.292
hxnM[4]	82+83	84+85	86+87	88+89	HZS.251,	122+123	EcoRI-	82+83	2,744 bp	3,040 bp	*J*	292, 293	FGSCA872,

	(3,284 bp)	(2,208 bp)	(3,328 bp)	(6,018 bp)	HZS.267		HindIII						HZS.297
hxnN[4]	90+91 (3,202 bp)	92+93 (2,208 bp)	94+95 (2,666 bp)	96+97 (5,841 bp)	TN02 A21	124+125	EcoRI-XbaI	90+91	4,045 bp	3,437 bp	*K*	288	FGSCA872
hxnST[8]	36+37 (2,825 bp)	38+31 (3,898 bp)	32+33 (2,645 bp)	39+35 (7,939 bp)	HZS.564	126+127	KpnI-HindIII	39+37	5,336 bp	6,500 bp	*L*	568	HZS.223, HZS.537, HZS.623
hxnZ[9]	17+18 (2,532 bp)	25+26 (2,410 bp)	21+22 (2,530 bp)	27+24 (6,478 bp)	HZS.221	114+115	EcoRV	21+22	6,207 bp	6,910 bp	*M*	480	-
hxnWV[10]	74+75 (2,030 bp)	76+69 (2,249 bp)	70+71 (3,953 bp)	80+73 (5787 bp)	HZS.404	128+129 130+131	XhoI	118+119 120+121	5,288 bp	-	*N*	749	-
hxnXW[11]	66+67 (2,662 bp)	68+59 (2208 bp)	62+63 (2,551 bp)	128+71 (6,015 bp)	HZS.404	130+131 116+117	EcoRV	116+117 118+119	3,546 bp	-	*O*	751	-
hxnXWV[12]	74+75 (2,030 bp)	76+59 (2,255 bp)	62+63 (2,551 bp)	80+73 (10,347 bp)	HZS.404	128+129 130+131 116+117	XbaI	116+117 118+119 120+121	10,347 bp	-	*P*	750	-

[*] Indicates the corresponding Southern blot images (from panel *A-P*) on Supplementary Fig. 11

[1] Recipient strain used for transformation

[2] Primers used for PCR are listed in Supplementary Table 5.

[3] Restriction enzymes used for the digestion of total DNA

[4] The selection marker used for gene-replacement was *riboB*$^{+}$

[5] The selection marker used for gene-replacement was *pabaA*$^{+}$

[6]The *hxnT* gene was deleted in a *hxnSΔ::pabaA*$^{+}$ recipient strain. The selection marker gene was *riboB*$^{+}$ from *A. nidulans*. The shared promoter between *hxnS* and *hxnT* remained intact in the developed *hxnSΔ::pabaA*$^{+}$ *hxnTΔ::riboB*$^{+}$ double deletion strain.

[7]The selection marker used for gene-replacement was *riboB*$^{+}$ from *A. fumigatus.*

[8] The gene-replacement cassette targeted both of the neighboring genes, *hxnS* and *hxnT* and the shared promoter region between them. The selection marker gene was *pabaA*$^{+}$ from *A. nidulans*. The gene replacement deleted *hxnS* and *hxnT* simultaneously.

[9] The *hxnZ* gene was deleted in a *hxnPΔ::riboB*$^{+}$ recipient strain. The selection marker gene was *pyroA*$^{+}$ from *A. nidulans*.

[10] The gene-replacement cassette targeted both of the neighboring genes, *hxnV* and *hxnW*. The selection marker gene was *riboB*$^{+}$ from *A. nidulans*. The gene replacement deleted *hxnV* and *hxnW* simultaneously.

[11] The gene-replacement cassette targeted both of the neighboring genes, *hxnX* and *hxnW*. The selection marker gene was *riboB*$^{+}$ from *A. nidulans*. The gene replacement deleted *hxnX* and *hxnW* simultaneously.

[12] The gene-replacement cassette targeted the three neighboring genes, *hxnX, hxnW* and *hxnV*. The selection marker gene was *riboB*$^{+}$ from *A. nidulans*. The gene replacement deleted *hxnX*, *hxnW* and *hxnV* simultaneously.

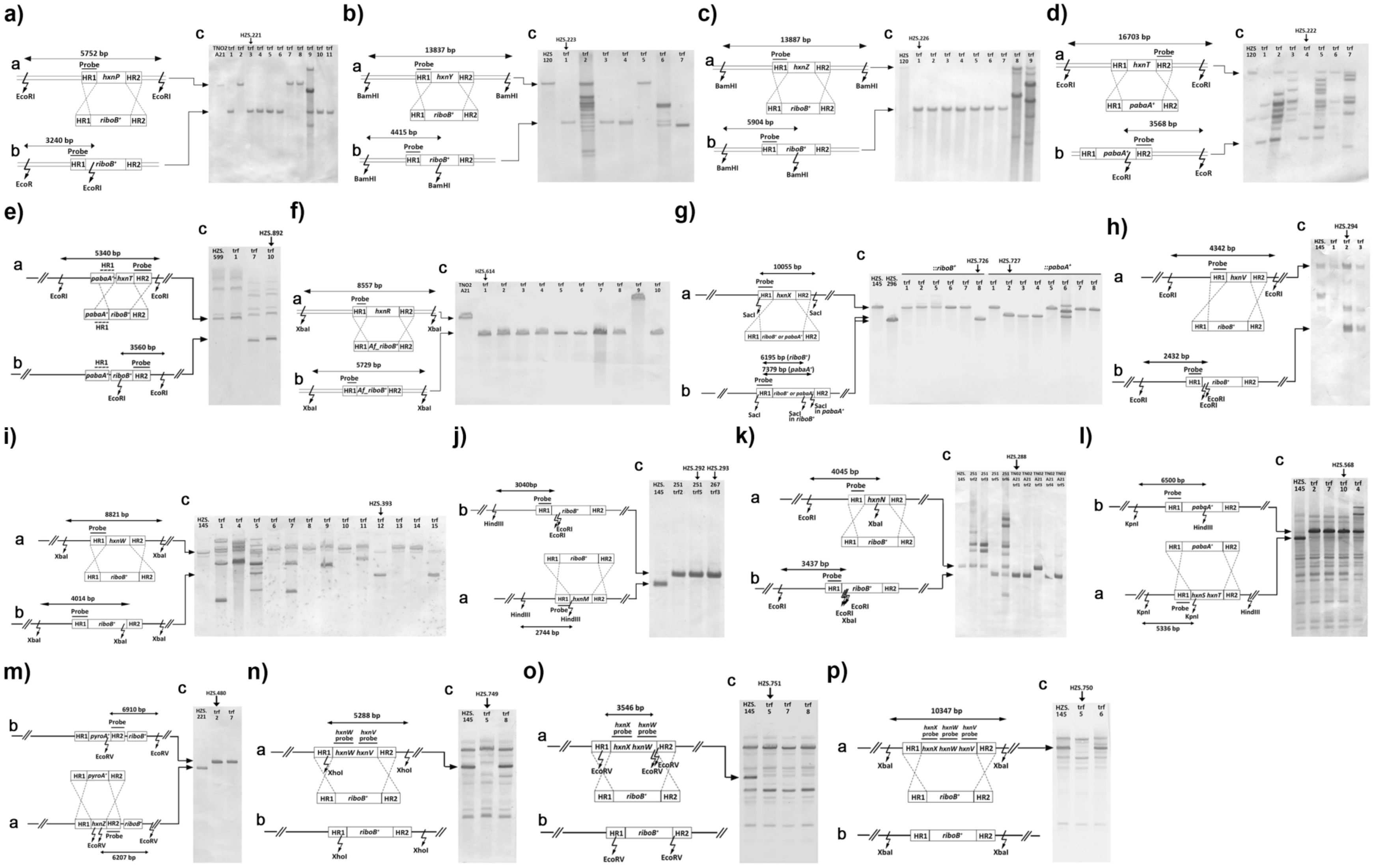

a)
5752 bp
Probe
HR1 hxnP HR2
EcoRI
HR1 riboB+ HR2
3240 bp
b)
13837 bp
HR1 hxnY HR2
BamHI
4415 bp
c)
13887 bp
HR1 hxnZ HR2
5904 bp
d)
16703 bp
HR1 hxnT HR2
HR1 pabaA+ HR2
3568 bp
e)
5340 bp
3560 bp
f)
8557 bp
HR1 hxnR HR2
XbaI
HR1 Af_riboB+ HR2
5729 bp
g)
10055 bp
HR1 hxnX HR2
SacI
6195 bp (riboB+)
7379 bp (pabaA+)
h)
4342 bp
HR1 hxnV HR2
2432 bp
i)
8821 bp
HR1 hxnW HR2
4014 bp
j)
3040bp
HindIII
HR1 hxnM HR2
2744 bp
k)
4045 bp
HR1 hxnN HR2
3437 bp
l)
6500 bp
KpnI
HR1 hxnS hxnT HR2
5336 bp
m)
6910 bp
HR1 pyroA+ HR2 riboB+
EcoRV
HR1 hxnZ HR2 riboB+
6207 bp
n)
5288 bp
hxnW probe
hxnV probe
HR1 hxnW hxnV HR2
XhoI
o)
3546 bp
hxnX probe
hxnW probe
HR1 hxnX hxnW HR2
EcoRV
p)
10347 bp
hxnX probe
hxnW probe
hxnV probe
HR1 hxnX hxnW hxnV HR2
XbaI

Supplementary Fig. 11. Southern blot analysis of gene-deletions in transformed strains. Panels **a)-p)** show the Southern blot results of transformant strains (images marked with *c*), the transformation cassettes constructed by using the DJ-PCR method together with the genomic layout of the targeted genes and their flanking regions in the recipient strain (drawings marked with *a*) and the layouts of the genomic areas after the gene-replacement events (drawings marked with *b*). Targeted gene(s) and the selection marker genes used for gene-replacements are marked in drawings by *a*. The crossing overs between the HR1 and HR2 regions (homologous recombination region 1 and 2) are indicated by crossing dashed lines. HR1 is the region that was used for homologous recombination upstream to the deletion site (corresponds to component "A" in the tripartite gene-replacement cassette); HR2 is the region that was used for homologous recombination downstream to the deletion site (corresponds to component "C" in the tripartite gene-replacement cassette). Zig-zag arrows show the positions of the cleavage sites of the restriction endonucleases used for the digestion of the total DNAs. The genomic regions used as DNA probes are shown above the corresponding genomic regions. The sizes of the hybridizing control and gene-replaced fragments are schematized above the corresponding regions by double-headed arrows. The *c* images show the Southern hybridization results (filter used:Hybond N from Amersham) where the first lanes show the control strains and the following lanes show the transformant strains (trf). Vertical arrows indicate the transformants, which were selected for further experiments. The cognate strain names are indicated above each vertical arrow, while their complete genotype is listed in Supplementary Table 3. Panels a)-p) refer to Southern blot data of gene replacements listed in Supplementary Table 6.

Supplementary Methods 2. Introduction of the dominant *hxnR^c7* allele *in trans* in the *hxnR⁺ hxnSΔ-hxnTΔ* double deletion and *hxnR⁺ hxnSΔ-hxnTΔ-hxnYΔ* triple deletion strains.

The plasmid pAN52-1 [35] was used to construct pAN-HZS-14 vector, from which the transformation vector, pAN-HZS-17 was constructed (Supplementary Fig. 12a, b, respectively). The pAN-HZS-14 vector was constructed by cloning the PCR product of the coding sequence of the *pyroA⁺* gene from wild-type *A. nidulans* (HZS.145) with its native promoter and termination sequence (by using the 132 and 133 primers) into the HindIII site of pAN52-1 [35] (Supplementary Fig. 12a). The *hxnR^c7* allele with its native promoter and termination sequence was amplified from FGSCA872 (by using the 134 and 135 primers) and cloned into the NheI-NotI sites of the pAN-HZS-14 vector. The NheI-NotI cleavage of the vector resulted in the complete elimination of the Gfp protein coding sequence from the vector and truncation of the P_{gpdA} promoter. The sequence of the cloned *hxnR^c7* allele was checked by sequencing the cloned region (with the primers from number 148 to 151). The resulting vector was named pAN-HZS-17 (Supplementary Fig. 12b). pAN-HZS-17 was transformed into the HZS.568 (*hxnSΔ-hxnTΔ*) and HZS.569 (*hxnSΔ-hxnTΔ-hxnYΔ*) recipient strains followed by the isolation of pyridoxine prototroph transformants. Integration of the vector was checked by PCR using primers 136 and 137 and the copy number of the integrated *hxnR^c7* construct was determined by qPCR using *actA* as a reference gene. The primer pairs used for qPCR were 162-163 and 154-155. The strains selected strains for further experiments were named HZS.911 (*hxnR^c7 hxnSΔ-hxnTΔ*) and HZS.912 (*hxnR^c7 hxnSΔ-hxnTΔ-hxnYΔ*) and they carried the integrated *hxnR^c7* vector in two and one copies, respectively.

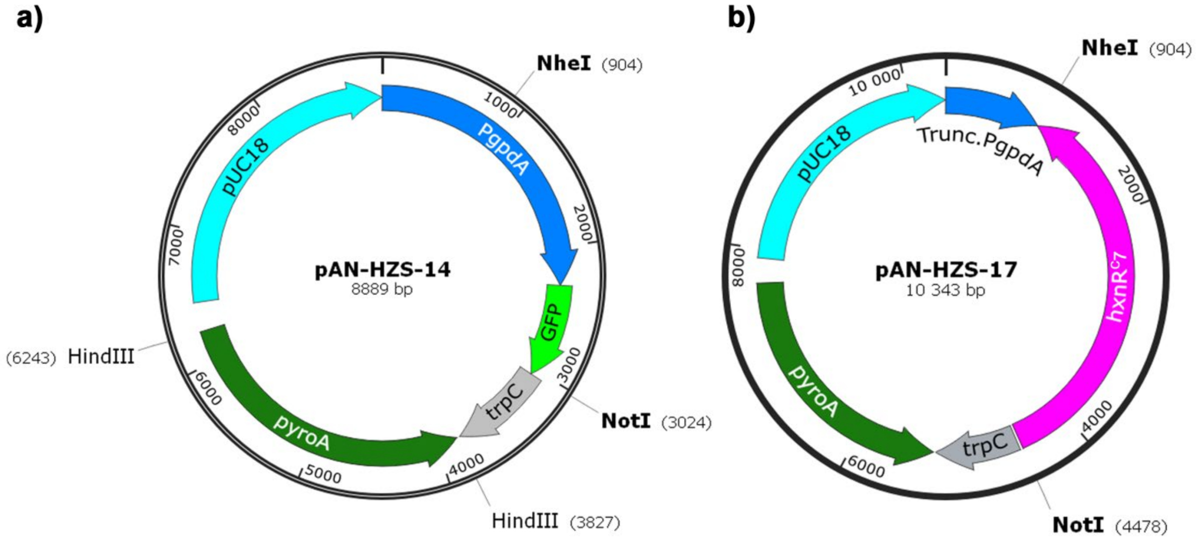

Fig. S12. Schematic presentation of the original and resulting transformation vectors used for the construction of the *hxnR^c7* expression strains. a) Original vector pAN-HZS-14 used for the construction of the transformation vector. **b)** Transformation vector pAN-HZS-17. Relevant unique restriction motifs used for cloning are shown (the numbers in parenthesis show the distance from the numbering start point in base pair units). Colored arrows show relevant components of the vectors (arrowheads indicate orientation). pUC18: standard *E. coli* vector; PgpdA: constitutive promoter of *gpdA* (glyceraldehyde-3-phosphate dehydrogenase coding gene) from *A. nidulans*; GFP: coding gene of Gfp (green fluorescence

protein); trpC: termination sequence of *trpC* gene (tryptophan biosynthesis gene) from *A. nidulans*; pyroA: wild-type *pyroA* gene (coding for pyridoxine biosynthesis gene) from *A. nidulans*, which serves as selection marker gene for transformation; Trunc.PgpdA: truncated *gpdA* promoter; hxnR^{c}7: constitutive allele of *hxnR* from *A. nidulans*. Names and sizes of the vectors are shown within the circular schemes.

Supplementary Methods 3. Construction of Gfp-HxnX expressing strains

Coding sequence of Gfp (green fluorescent protein) was fused to the 5' end of *hxnX* by Double-Joint PCR (DJ-PCR) [34]. *gfp* was amplified from a pAN-HZS-1 [33] (Supplementary Fig. 13a) template using the 138 and 139 primers. The reverse primer 139 was specific to the 3'end of *gfp* (excluding the stop codon) and carried a 24 bp long linker sequence at the 5'end that encoded 8 AAs (amino acids) (LIDTVDLD). *hxnX* was amplified from a wild-type template (HZS.145) using the 140 and 73 primers. The forward primer 140 was specific to the 5' terminus of *hxnX* (start codon included) and carried the 8 AAs linker-coding sequence at the 5' terminus. The amplified *gfp* and *hxnX* PCR products were combined into a single molecule by DJ-PCR using nested forward and nested reverse primers carrying NcoI and NotI and motifs at their 5' ends, respectively (141 and 142). The resulted *gfp-hxnX* fusion PCR product was cloned into an NcoI-NotI digested pAN-HZS-1 (Supplementary Fig. 13a) (upon cleavage, the *gfp* gene had been eliminated from the original vector). The resulting vector (named as pAN-HZS-13) expressed the *gfp-hxnX* fusion from the constitutive *gpdA* promoter and carried the *pantoB*$^{+}$ gene that served as selection marker gene for transformation (Supplementary Fig. 13b). pAN-HZS-13 was transformed into a peroxisome labeled (DsRed-SKL expressing) [24,36] *hxnXΔ* strain (HZS.534). Transformants carrying the *gfp-hxnX* transgene from 1-10 copies were isolated. Copy number of *gfp-hxnX* transgene was determined by qPCR carried out with *hxnX* specific 156 and 157 and *actA* specific 162 and 163 primer pairs. The copy number of the *gfp-hxnX* transgene varied from 1-10 in the different transformants. All strains with *gfp-hxnX* transgene showed co-localization of green fluorescence with peroxisome-specific red fluorescence. Since the intensity of Gfp fluorescence was very low in strains with only 1 transgene compared to the intensity of DsRed fluorescence, we selected the strain with 7 copies of transgene (HZS.579) for fluorescence microscopy.

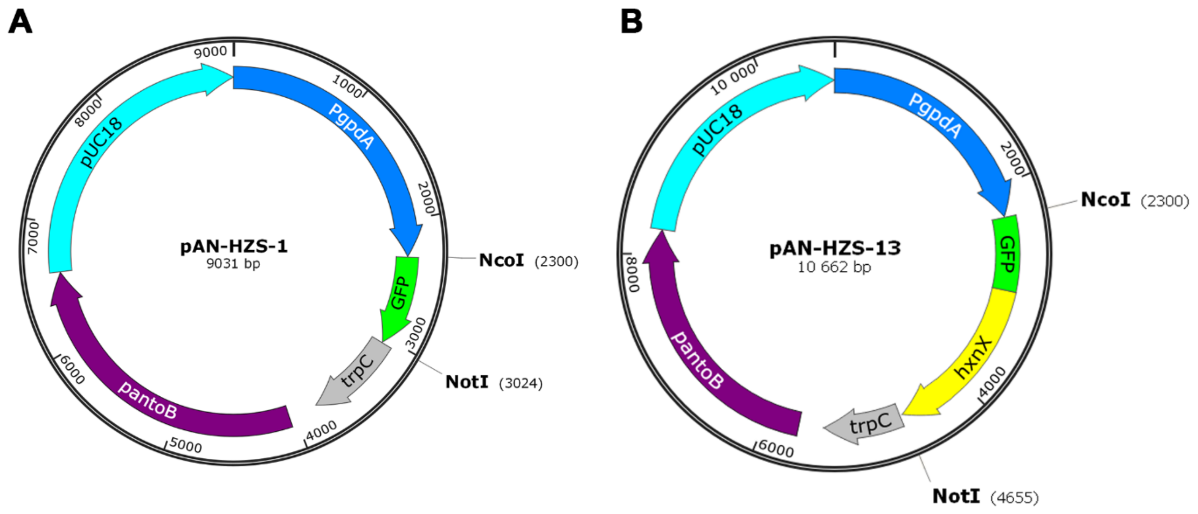

Supplementary Fig. 13. Schematic representation of the original and resulting transformation vectors used for the construction of the *gfp-hxnX* expression strains. a) Original vector pAN-HZS-1 used for the construction of the transformation vector [33]. **b)** Transformation vector pAN-HZS-13. Relevant unique restriction motifs used for cloning are shown (the numbers in parenthesis show the distance from the numbering start point in base pair units). Colored arrows show relevant components of the vectors (arrowheads indicate orientation). pUC18: standard *E. coli* vector; PgpdA: constitutive promoter of *gpdA* (glyceraldehyde-3-phosphate dehydrogenase encoding gene) from *A. nidulans*; GFP: coding gene of Gfp (green fluorescence protein); trpC: termination sequence of *trpC* gene (tryptophan biosynthesis gene) from *A. nidulans*; pantoB: wild-type *pantoB* gene (coding for a pantothenic acid biosynthesis gene) from *A. nidulans* serving as a selection marker for transformation; gfp-hxnX: *gfp* fused *hxnX* gene carrying a 8 AA coding linker sequence between *gfp* and *hxnX*. Names and sizes of the vectors are shown within the circular schemes.

SUPPLEMENTARY REFERENCES

1 Kelley, L. A., Mezulis, S., Yates, C. M., Wass, M. N. & Sternberg, M. J. The Phyre2 web portal for protein modeling, prediction and analysis. *Nat Protoc* **10**, 845-858, doi:10.1038/nprot.2015.053 (2015).

2 Llorente, B. & Dujon, B. Transcriptional regulation of the *Saccharomyces cerevisiae DAL5* gene family and identification of the high affinity nicotinic acid permease *TNA1* (*YGR260w*). *FEBS letters* **475**, 237-241, doi:10.1016/s0014-5793(00)01698-7 (2000).

3 Amon, J. *et al.* A eukaryotic nicotinate-inducible gene cluster: convergent evolution in fungi and bacteria. *Open Biol* **7**, 170199, doi:10.1098/rsob.170199 (2017).

4 Sibthorp, C. *et al.* Transcriptome analysis of the filamentous fungus *Aspergillus nidulans* directed to the global identification of promoters. *BMC Genomics* **14**, 847, doi:10.1186/1471-2164-14-847 (2013).

5 Cerqueira, G. C. *et al.* The *Aspergillus* Genome Database: multispecies curation and incorporation of RNA-Seq data to improve structural gene annotations. *Nucleic acids research* **42**, D705-710, doi:10.1093/nar/gkt1029 (2014).

6 Larionov, A., Krause, A. & Miller, W. A standard curve based method for relative real time PCR data processing. *BMC Bioinformatics* **6**, 62, doi:10.1186/1471-2105-6-62 (2005).

7 Nakano, H. *et al.* Purification, characterization and gene cloning of 6-hydroxynicotinate 3-monooxygenase from *Pseudomonas fluorescens* TN5. *Eur J Biochem* **260**, 120-126, doi:10.1046/j.1432-1327.1999.00124.x (1999).

8 Scazzocchio, C. The genetic control of molybdoflavoproteins in *Aspergillus nidulans*. II. Use of NADH dehydrogenase activity associated with xanthine dehydrogenase to investigate substrate and product inductions. *Mol Gen Genet* **125**, 147-155, doi:10.1007/BF00268868 (1973).

9 Sealy-Lewis, H. M., Lycan, D. & Scazzocchio, C. Product induction of purine hydroxylase II in *Asperigillus nidulans*. *Mol Gen Genet* **174**, 105-106 (1979).

10 Enroth, C. High-resolution structure of phenol hydroxylase and correction of sequence errors. *Acta Crystallogr D Biol Crystallogr* **59**, 1597-1602, doi:10.1107/s0907444903014902 (2003).

11 Enroth, C., Neujahr, H., Schneider, G. & Lindqvist, Y. The crystal structure of phenol hydroxylase in complex with FAD and phenol provides evidence for a concerted conformational change in the enzyme and its cofactor during catalysis. *Structure* **6**, 605-617, doi:10.1016/s0969-2126(98)00062-8 (1998).

12 Pompeu, Y. A., Sullivan, B., Walton, A. Z. & D., S. J. Structural and Catalytic Characterization of *Pichia stipitis* OYE2.6, a Useful Biocatalyst for Asymmetric Alkene Reductions. *Adv Synth Catal* **354**, 1949-1960 (2012).

13 Stott, K., Saito, K., Thiele, D. J. & Massey, V. Old Yellow Enzyme. The discovery of multiple isozymes and a family of related proteins. *The Journal of biological chemistry* **268**, 6097-6106 (1993).

14 Fox, K. M. & Karplus, P. A. Old yellow enzyme at 2 A resolution: overall structure, ligand binding, and comparison with related flavoproteins. *Structure* **2**, 1089-1105, doi:10.1016/S0969-2126(94)00111-1 (1994).

15 Liu, X., Yuan, Z., Adam Yuan, Y., Lin, J. & Wei, D. Biochemical and structural analysis of Gox2181, a new member of the SDR superfamily from *Gluconobacter oxydans*. *Biochem Biophys Res Commun* **415**, 410-415, doi:10.1016/j.bbrc.2011.10.083 (2011).

16 Li, W., Zhang, T. & Ding, J. Molecular basis for the substrate specificity and catalytic mechanism of thymine-7-hydroxylase in fungi. *Nucleic acids research* **43**, 10026-10038, doi:10.1093/nar/gkv979 (2015).
17 Jimenez, J. I. *et al.* Deciphering the genetic determinants for aerobic nicotinic acid degradation: the *nic* cluster from *Pseudomonas putida* KT2440. *Proceedings of the National Academy of Sciences of the United States of America* **105**, 11329-11334, doi:10.1073/pnas.0802273105 (2008).
18 Shaik, M. M., Cendron, L., Percudani, R. & Zanotti, G. The structure of *Helicobacter pylori* HP0310 reveals an atypical peptidoglycan deacetylase. *PLoS One* **6**, e19207, doi:10.1371/journal.pone.0019207 (2011).
19 Bhattacharjee, N., Feliks, M., Shaik, M. M. & Field, M. J. Catalytic Mechanism of Peptidoglycan Deacetylase: A Computational Study. *J Phys Chem B* **121**, 89-99, doi:10.1021/acs.jpcb.6b10625 (2017).
20 Shin, S. *et al.* Structure of malonamidase E2 reveals a novel Ser-cisSer-Lys catalytic triad in a new serine hydrolase fold that is prevalent in nature. *The EMBO journal* **21**, 2509-2516, doi:10.1093/emboj/21.11.2509 (2002).
21 Mileni, M. *et al.* Structure-guided inhibitor design for human FAAH by interspecies active site conversion. *Proceedings of the National Academy of Sciences of the United States of America* **105**, 12820-12824, doi:10.1073/pnas.0806121105 (2008).
22 Kafer, E. Origins of translocations in *Aspergillus nidulans*. *Genetics* **52**, 217-232 (1965).
23 Nayak, T. *et al.* A versatile and efficient gene-targeting system for *Aspergillus nidulans*. *Genetics* **172**, 1557-1566, doi:10.1534/genetics.105.052563 (2006).
24 Flipphi, M., Oestreicher, N., Nicolas, V., Guitton, A. & Velot, C. The *Aspergillus nidulans acuL* gene encodes a mitochondrial carrier required for the utilization of carbon sources that are metabolized via the TCA cycle. *Fungal genetics and biology : FG & B* **68**, 9-22, doi:10.1016/j.fgb.2014.04.012 (2014).
25 Hamari, Z. *et al.* Convergent evolution and orphan genes in the Fur4p-like family and characterization of a general nucleoside transporter in *Aspergillus nidulans*. *Molecular microbiology* **73**, 43-57, doi:10.1111/j.1365-2958.2009.06738.x (2009).
26 Sinha, U. Genetic control of the uptake of amino acids in *Aspergillus nidulans*. *Genetics* **62**, 495-505, doi:10.1093/genetics/62.3.495 (1969).
27 Clutterbuck, A. J. Absence of laccase from yellow-spored mutants of *Aspergillus nidulans*. *J Gen Microbiol* **70**, 423-435, doi:10.1099/00221287-70-3-423 (1972).
28 Buxton, F. P. & Radford, A. Cloning of the structural gene for orotidine 5'-phosphate carboxylase of *Neurospora crassa* by expression in *Escherichia coli*. *Mol Gen Genet* **190**, 403-405, doi:10.1007/BF00331067 (1983).
29 Hicks, K. A. *et al.* Structural and Biochemical Characterization of 6-Hydroxynicotinic Acid 3-Monooxygenase, A Novel Decarboxylative Hydroxylase Involved in Aerobic Nicotinate Degradation. *Biochemistry* **55**, 3432-3446, doi:10.1021/acs.biochem.6b00105 (2016).
30 Hiromoto, T., Fujiwara, S., Hosokawa, K. & Yamaguchi, H. Crystal structure of 3-hydroxybenzoate hydroxylase from *Comamonas testosteroni* has a large tunnel for substrate and oxygen access to the active site. *Journal of molecular biology* **364**, 878-896, doi:10.1016/j.jmb.2006.09.031 (2006).
31 Zhang, Y. I-TASSER server for protein 3D structure prediction. *BMC Bioinformatics* **9**, 40, doi:10.1186/1471-2105-9-40 (2008).
32 Xu, D. & Zhang, Y. Improving the physical realism and structural accuracy of protein models by a two-step atomic-level energy minimization. *Biophys J* **101**, 2525-2534, doi:10.1016/j.bpj.2011.10.024 (2011).

33 Karacsony, Z., Gacser, A., Vagvolgyi, C., Scazzocchio, C. & Hamari, Z. A dually located multi-HMG-box protein of *Aspergillus nidulans* has a crucial role in conidial and ascospore germination. *Molecular microbiology* **94**, 383-402, doi:10.1111/mmi.12772 (2014).
34 Yu, J. H. *et al.* Double-joint PCR: a PCR-based molecular tool for gene manipulations in filamentous fungi. *Fungal genetics and biology : FG & B* **41**, 973-981, doi:10.1016/j.fgb.2004.08.001 (2004).
35 Punt, P. J., Oliver, R. P., Dingemanse, M. A., Pouwels, P. H. & van den Hondel, C. A. Transformation of *Aspergillus* based on the hygromycin B resistance marker from *Escherichia coli*. *Gene* **56**, 117-124 (1987).
36 Magliano, P., Flipphi, M., Arpat, B. A., Delessert, S. & Poirier, Y. Contributions of the peroxisome and beta-oxidation cycle to biotin synthesis in fungi. *The Journal of biological chemistry* **286**, 42133-42140, doi:10.1074/jbc.M111.279687 (2011).
