# Supplementary material for: A complete nicotinate degradation pathway in the microbial eukaryote Aspergillus nidulans
Source: Commun Biol. 2022 Jul 21;5:723. doi: 10.1038/s42003-022-03684-3 (PMC9304392; doi:10.1038/s42003-022-03684-3)
Supplement: Supplementary file 2 — Reporting Summary [file 42003_2022_3684_MOESM2_ESM.pdf]

nature portfolio

Corresponding author(s): Zsuzsanna Hamari

Last updated by author(s): May 24, 2022

Reporting Summary

Nature Portfolio wishes to improve the reproducibility of the work that we publish. This form provides structure for consistency and transparency in reporting. For further information on Nature Portfolio policies, see our Editorial Policies and the Editorial Policy Checklist.

Statistics

For all statistical analyses, confirm that the following items are present in the figure legend, table legend, main text, or Methods section.

n/a	Confirmed	
☐	☒	The exact sample size (*n*) for each experimental group/condition, given as a discrete number and unit of measurement
☐	☒	A statement on whether measurements were taken from distinct samples or whether the same sample was measured repeatedly
☐	☒	The statistical test(s) used AND whether they are one- or two-sided<br>*Only common tests should be described solely by name; describe more complex techniques in the Methods section.*
☒	☐	A description of all covariates tested
☒	☐	A description of any assumptions or corrections, such as tests of normality and adjustment for multiple comparisons
☒	☐	A full description of the statistical parameters including central tendency (e.g. means) or other basic estimates (e.g. regression coefficient) AND variation (e.g. standard deviation) or associated estimates of uncertainty (e.g. confidence intervals)
☒	☐	For null hypothesis testing, the test statistic (e.g. *F*, *t*, *r*) with confidence intervals, effect sizes, degrees of freedom and *P* value noted<br>*Give P values as exact values whenever suitable.*
☒	☐	For Bayesian analysis, information on the choice of priors and Markov chain Monte Carlo settings
☒	☐	For hierarchical and complex designs, identification of the appropriate level for tests and full reporting of outcomes
☒	☐	Estimates of effect sizes (e.g. Cohen's *d*, Pearson's *r*), indicating how they were calculated

Our web collection on statistics for biologists contains articles on many of the points above.

Software and code

Policy information about availability of computer code

Data collection	No software was used for data collection.
Data analysis	Softwares used for data analysis are cited appropriately.

For manuscripts utilizing custom algorithms or software that are central to the research but not yet described in published literature, software must be made available to editors and reviewers. We strongly encourage code deposition in a community repository (e.g. GitHub). See the Nature Portfolio guidelines for submitting code & software for further information.

Data

Policy information about availability of data

All manuscripts must include a data availability statement. This statement should provide the following information, where applicable:

- Accession codes, unique identifiers, or web links for publicly available datasets
- A description of any restrictions on data availability
- For clinical datasets or third party data, please ensure that the statement adheres to our policy

All experimental data are shown in either the main text or in the Supplementary files (Supplementary Figures, Supplementary Tables and Supplementary Methods).

Human research participants

Policy information about studies involving human research participants and Sex and Gender in Research.

Reporting on sex and gender	This is not relevant to our study. The research was conducted on a fungal model organism, Aspergillus nidulans.
Population characteristics	This is not relevant to our study. The research was conducted on a fungal model organism, Aspergillus nidulans.
Recruitment	This is not relevant to our study. The research was conducted on a fungal model organism, Aspergillus nidulans.
Ethics oversight	This is not relevant to our study. The research was conducted on a fungal model organism, Aspergillus nidulans.

Note that full information on the approval of the study protocol must also be provided in the manuscript.

Field-specific reporting

Please select the one below that is the best fit for your research. If you are not sure, read the appropriate sections before making your selection.

☒ Life sciences ☐ Behavioural & social sciences ☐ Ecological, evolutionary & environmental sciences

For a reference copy of the document with all sections, see nature.com/documents/nr-reporting-summary-flat.pdf

Life sciences study design

All studies must disclose on these points even when the disclosure is negative.

Sample size	The UHPLC-HRMS measurements were carried out at least in three biological replicates for each mutant. For single deletion mutants the measurements were carried out in six biological replicates. The coefficient of variation was less than 10% in each case. Some multi-deletion strains, can be consider as further biological replicates of relevant single deletion mutants, as multiple these deletion strains accumulate the same metabolite as the most upstream single deletion mutant. This can be easily seen on the heat map presented in Figure 4.
Data exclusions	No data were excluded from the reported analyses.
Replication	The experimental findings are reproducible. The phenotypes of the various single- and multi-deletion mutants are stable. A substantial number of strains carrying a given deletion were constructed and included in the experiments, which made the experimental setup redundant. Multi-deletion mutants carried deletions of eight pathway genes in various combinations. They facilitated the establishment of the order of the enzymes in the pathway and reinforced the conclusions derived from the growth phenotypes and metabolite profiling of the single deletion strains.
Randomization	Randomization was not applicable in the presented research.
Blinding	The samples used for in vitro metabolite profiling analysis were blinded for the analytic measurements. The identities of the blinded samples were only disclosed after the metabolite analysis was completed.

Reporting for specific materials, systems and methods

We require information from authors about some types of materials, experimental systems and methods used in many studies. Here, indicate whether each material, system or method listed is relevant to your study. If you are not sure if a list item applies to your research, read the appropriate section before selecting a response.

Materials & experimental systems

n/a	Involved in the study	
☒	☐	Antibodies
☒	☐	Eukaryotic cell lines
☒	☐	Palaeontology and archaeology
☒	☐	Animals and other organisms
☒	☐	Clinical data
☒	☐	Dual use research of concern

Methods

n/a	Involved in the study	
☒	☐	ChIP-seq
☒	☐	Flow cytometry
☒	☐	MRI-based neuroimaging
